# Comparative analysis of the association between 35 frailty scores and cardiovascular events, cancer, and total mortality in an elderly general population in England: An observational study

**DOI:** 10.1371/journal.pmed.1002543

**Published:** 2018-03-27

**Authors:** Gloria A. Aguayo, Michel T. Vaillant, Anne-Françoise Donneau, Anna Schritz, Saverio Stranges, Laurent Malisoux, Anna Chioti, Michèle Guillaume, Majon Muller, Daniel R. Witte

**Affiliations:** 1 Population Health Department, Luxembourg Institute of Health, Strassen, Luxembourg; 2 Competence Center for Methodology and Statistics, Luxembourg Institute of Health, Strassen, Luxembourg; 3 Department of Public Health, University of Liège, Liège, Belgium; 4 Department of Epidemiology & Biostatistics, Schulich School of Medicine & Dentistry, University of Western Ontario, London, Canada; 5 Section of Geriatrics, Department of Internal Medicine, VU University Medical Center, Amsterdam, the Netherlands; 6 Department of Public Health, Aarhus University, Aarhus, Denmark; 7 Danish Diabetes Academy, Odense, Denmark; University of Oxford, UNITED KINGDOM

## Abstract

**Background:**

Frail elderly people experience elevated mortality. However, no consensus exists on the definition of frailty, and many frailty scores have been developed. The main aim of this study was to compare the association between 35 frailty scores and incident cardiovascular disease (CVD), incident cancer, and all-cause mortality. Also, we aimed to assess whether frailty scores added predictive value to basic and adjusted models for these outcomes.

**Methods and findings:**

Through a structured literature search, we identified 35 frailty scores that could be calculated at wave 2 of the English Longitudinal Study of Ageing (ELSA), an observational cohort study. We analysed data from 5,294 participants, 44.9% men, aged 60 years and over. We studied the association between each of the scores and the incidence of CVD, cancer, and all-cause mortality during a 7-year follow-up using Cox proportional hazard models at progressive levels of adjustment. We also examined the added predictive performance of each score on top of basic models using Harrell’s C statistic. Using age of the participant as a timescale, in sex-adjusted models, hazard ratios (HRs) (95% confidence intervals) for all-cause mortality ranged from 2.4 (95% CI: 1.7–3.3) to 26.2 (95% CI: 15.4–44.5). In further adjusted models including smoking status and alcohol consumption, HR ranged from 2.3 (95% CI: 1.6–3.1) to 20.2 (95% CI: 11.8–34.5). In fully adjusted models including lifestyle and comorbidity, HR ranged from 0.9 (95% CI: 0.5–1.7) to 8.4 (95% CI: 4.9–14.4). HRs for CVD and cancer incidence in sex-adjusted models ranged from 1.2 (95% CI: 0.5–3.2) to 16.5 (95% CI: 7.8–35.0) and from 0.7 (95% CI: 0.4–1.2) to 2.4 (95% CI: 1.0–5.7), respectively. In sex- and age-adjusted models, all frailty scores showed significant added predictive performance for all-cause mortality, increasing the C statistic by up to 3%. None of the scores significantly improved basic prediction models for CVD or cancer. A source of bias could be the differences in mortality follow-up time compared to CVD/cancer, because the existence of informative censoring cannot be excluded.

**Conclusion:**

There is high variability in the strength of the association between frailty scores and 7-year all-cause mortality, incident CVD, and cancer. With regard to all-cause mortality, some scores give a modest improvement to the predictive ability. Our results show that certain scores clearly outperform others with regard to three important health outcomes in later life. Finally, we think that despite their limitations, the use of frailty scores to identify the elderly population at risk is still a useful measure, and the choice of a frailty score should balance feasibility with performance.

## Abstract 2

Using data from the English Longitudinal Study of Ageing, Gloria Aguayo and colleagues compare the association between 35 frailty scores and cardiovascular events, cancer, and total mortality.

## Author summary

**Why was this study done?:**

- Frailty has been associated with poor outcomes in the elderly, and the need for valid instruments for its assessment is generally recognised.
- Many frailty scores have been developed based on different theoretical concepts; however, none of them can be considered the gold standard.
- The predictive capacity of frailty scores has mainly been studied for mortality, and evidence on their ability to predict other important outcomes in the elderly population, such as cardiovascular or cancer events, is limited (cardiovascular) or nonexistent (cancer) at the present time.

**What did the researchers do and find?:**

- We performed secondary analyses of the most comprehensive list of frailty scores in 5,294 participants of the English Longitudinal Study of Ageing, and we demonstrated that all frailty scores were associated with future mortality and that some of them were also associated with later cardiovascular events. However, no relationship with cancer was observed.
- In addition, the results of this study showed that multidimensional frailty scores may have a stronger and more stable association with mortality and incidence of cardiovascular events.
- Beside the results evidenced that despite significant associations of frailty scores with mortality, the added discriminative ability of frailty scores to chronological age may be limited.

**What do these findings mean?:**

- The comparative evaluation of the strength of associations between different frailty scores and major health outcomes in older adults provides a solid evidence base for researchers, clinicians, and health professionals working with elderly populations to choose the most appropriate instrument for their needs.
- Furthermore, the findings highlight the vast heterogeneity in composition and performance of existing frailty scores.

## Introduction

Although chronological age is the strongest determinant of disease occurrence and mortality, it is increasingly recognised that the process of ageing is heterogeneous [1] due to a combination of differences in lifetime cumulative exposure to determinants of chronic disease and differences in individual susceptibility. The concept of frailty was introduced as a way of identifying individuals who, at a given age, have a particularly fragile health balance and are therefore more vulnerable to rapid health deterioration and early mortality [2]. However, the operationalization of the concept of frailty has been fraught with difficulties, as different groups of researchers and clinicians have expressed diverging views on which characteristics make up frailty and on how these should be assessed individually and in unison.

Considering the type and composition of variables of frailty scores, four main approaches to frailty can be distinguished. First, the “phenotype of frailty” approach describes frailty as a physiological syndrome of diminished resistance to stressors associated with poor health outcomes [3]. Second, the “multidimensional” approach defines frailty as a dynamic process of loss of function in one or more domains, making the individual vulnerable [4]. Third, the “accumulation of deficit” approach counts the number of health problems or deficits to classify the individual as frail [5]. Fourth, we propose a "disability" approach, as frailty scores were created primarily with variables representing a degree of disability. We have included this classification even without a theoretical basis/reference, as these scores are used as frailty scores, although disability is considered by many authors more as a result of frailty or an overlap condition than as an equivalent of frailty [6].

There is no gold standard to measure frailty and many different frailty scores have been created, even within each of the four main approaches [7]. We have previously shown that there is only limited agreement in which individuals will be classified as frail, according to different scores, and that, in consequence, it is impossible to compare the prevalence of frailty or associations with relevant outcomes between studies using different frailty scores directly [8].

To fully assess and compare the performance of different frailty scores, it is also necessary to consider their prospective association and predictive ability for the main conditions that cause the loss of healthy life years and quality of life in an ageing population [9]. Prospective associations were used in this study to investigate frailty scores as risk factors of important outcomes in the elderly population: death or cardiovascular or cancer events [10]. Predictive value was used in this study to determine the ability of frailty scores to discriminate or separate participants who will from those who will not develop an event [11].

Many scores have shown strong associations with all-cause mortality, risk of hospitalization, and disability [7], but the knowledge concerning their association with other major causes of ill-health and loss of quality of life, such as cardiovascular disease (CVD) events and cancer, is very limited. In a longitudinal study, Klein et al. found a significant association between frailty and CVD (odds ratio [OR] in men = 1.33 [1.06–1.67]; in women = 1.43 [1.13–1.82]) and a slightly high, although not significant, association between frailty and cancer (ORin men = 1.17 [0.89–1.55]; in women = 1.21 [0.95–1.54]) [12]. Another study shows associations between variables that take part of some frailty instruments and cancer incidence [13], but no direct large-scale comparison studies are available.

This comparative analysis is important beyond the fact that this has not been done. Researchers need more information on what frailty scores actually measure and how they can compare or pool results of studies using different frailty scores. Clinicians need more information on the performance of the scores and on the most appropriate instruments in clinical settings. Policy makers need more information on the usefulness of measuring frailty at a population level and how to achieve it with the best instruments.

Therefore, the objective of this study was to carry out a comparative external validation of a comprehensive list of frailty scores with regard to three important health outcomes in later life: CVD, cancer, and all-cause mortality, by direct comparison of the strength of associations and of added predictive value, using prospective data from a population-based study in the elderly. Some of the scales included are composite scales for physical activity or function, grouped as frailty scores for this paper.

Our hypothesis was that the marked heterogeneity in approach, type, and composition of frailty scores would translate into heterogeneity in associations and predictive ability, with important health outcomes.

## Methods

### Participants, inclusion criteria, and study design

#### Participants

Data on participants from the English Longitudinal Study of Ageing (ELSA) were used under data-sharing project number 82538. ELSA is an ongoing longitudinal cohort study based on a representative sample of middle-aged and elderly general population 50 years and over living in England [14]. ELSA has extensive subjective and objective information collected in biennial surveys (waves). All waves gathered information concerning physical, cognitive, and psychological health, disability, lifestyle factors, comorbidities, social participation, and social support. Also, even-numbered waves have objective measures: physical functioning assessment and biological sampling [15]. Ethical approval was obtained from the Multicentre Research and Ethics Committee and all participants provided written informed consent [16].

#### Inclusion criteria

Participants aged 60 or over (because not all frailty-related variables were measured in participants younger than 60 years) who gave permission to link their data with a national mortality register and had a nurse visit in wave 2 were included. The outcomes were measured up to 2012, when mortality data were assessed.

### Study design

This is a longitudinal secondary data analysis of ELSA and no formal written analysis plan exists. The analysis was planned in November 2015 during meetings with coauthors. We used the second wave (2004–2005) as baseline because this was the first wave with a clinical examination and laboratory samples. The exposure was the frailty state measured with 35 different frailty scores at baseline, and the follow-up time was from 2004–2005 to 2012.

### Frailty scores

A structured search was performed to identify all published original frailty scores. The search strategy has previously been described in detail [8].

The original scores that could be calculated with the ELSA wave 2 data (i.e., those for which at least 80% of the necessary variables were measured) were selected. Multiple imputation was used to deal with missing data in the underlying measured study variables necessary to calculate the frailty scores. In order to obtain optimally plausible values for the scores, imputation was applied to the original underlying variables, and frailty scores were calculated a posteriori using imputed values.

For preparing an analysis in one single continuous scale, frailty scores were rescaled from 0 (non-frail) to 1 (maximum frail) by dividing the output of each frailty score by the maximum possible value. If the frailty score was defined with a score that gave different weight to some variables, the output was accorded this weight and then rescaled. In addition, some frailty scores had to be inverted to convert the result, according the definition of 0 as non-frail and 1 as maximum frail.

Scores were classified into 4 groups depending on their underlying frailty approach: phenotype of frailty (mainly physical functioning variables), multidimensional (at least 2 different dimensions and less than 30 variables), accumulation of deficits (at least 30 variables), and disability (mainly disability variables).

A total of 67 original frailty scores were found in the literature search and 35 had at least 80% of variables possible to calculate with the data of ELSA wave 2, and in consequence, they were selected (Table 1). Out of them, 19 had binary cutoffs identifying frail and non-frail individuals, and 10 had categorical cutoffs, additionally identifying an intermediate pre-frail group [8].

**Table 1:** Frailty scores calculated in participants of ELSA wave 2 (2004–2005).

First Author, Year (Reference)	Score Name	Abbreviation
**Phenotype of frailty approach**		
Klein, 2003 [17]	Beaver Dam Eye Study Index	BDE
Cesari, 2014 [18]	Frail Non-Disabled (FiND) Questionnaire	FiND
van Kan, 2008 [19]	Frail Scale	FS
Rothman, 2008 [20]	Modified Phenotype of Frailty	MPHF
Gill, 2002 [21]	Physical Frailty Index	PFI
Fried, 2001 [3]	Phenotype of Frailty	PHF
Ensrud, 2007 [22]	Study of Osteoporotic Fractures	SOF
Guralnik, 1994 [23]	Short Physical Performance Battery	SPPB
Chin, 1999 [24]	ZutPhen Elderly Study (Physical Activity and Low BMI)	ZED1
Chin, 1999 [24]	ZutPhen Elderly Study (Physical Activity and Weight Loss)	ZED2
Chin, 1999 [24]	ZutPhen Elderly Study (Physical Activity and Low Energy)	ZED3
**Multidimensional approach**		
Freiheit, 2010 [25]	Brief Frailty Index	BFI
Balducci, 2000 [26]	Comprehensive Geriatric Assessment Screening Tests	CGAST
Ravaglia, 2008 [27]	Conselice Study of Brain Aging Score	CSBA
Rolfson, 2006 [28]	Edmonton Frail Scale	EFS
Cacciatore, 2005 [29]	Frailty Staging System	FSS
Bellera, 2012 [30]	G-8 Geriatric Screening Tool	G8
Steverink, 2001 [31]	Groningen Frailty Indicator	GFI
Brody, 1997 [32]	Health Status Form	HSF
Di Bari, 2014 [33]	Inter-Frail Questionnaire	IFQ
Hubbard, 2009 [34]	Modified Frailty Score	MFS
Puts, 2005 [35]	Static/Dynamic Frailty Index	SDFI
Maly, 1997 [36]	Screening Instrument	SI
Hébert, 1996 [37]	Sherbrooke Postal Questionnaire	SPQ
Gobbens, 2010 [38]	Tilburg Frailty Indicator	TFI
**Accumulation of deficits approach**		
Jones, 2004 [39]	Comprehensive Geriatric Assessment	CGA
de Vries, 2013 [40]	Evaluative Frailty Index for Physical Activity	EFIP
Searle, 2008 [41]	40-item Frailty Index	FI40
Theou, 2013 [42]	70-item Frailty Index Survey of Health, Ageing and Retirement in Europe	FI70
Fang, 2012 [43]	Frailty Index Beijing Longitudinal Study of Ageing	FIBLSA
Kulminski, 2007 [44]	Long Term Care Survey Frailty Index	NLTCS
**Disability approach**		
Morris, 1984 [45]	Hebrew Rehabilitation Center for Aged Vulnerability Index	HRCA
Rockwood, 2005 [46]	Canadian Study of Health and Aging Clinical Frailty Scale	SHCFS
Saliba, 2001 [47]	Vulnerable Elders Survey	VES13
Dayhoff, 1998 [48]	World Health Organization Assessment of Functional Capacity and self-reported health	WHRH

**Abbreviation:** ELSA, English Longitudinal Study of Ageing.

### Missing data

Missing data of some needed variables to calculate frailty scores were observed in 1 (<1.0%) to 3,037 (57.4%) participants. The mechanism of missing data was assumed to be missing at random because the underlying values necessary to calculate frailty scores that were missing for some individuals are likely to depend on observed data in the ELSA data. In other words, missing data did not depend on any unobserved data, but only upon observed data.

Each variable was defined as being of numerical, binary, or categorical type, which defined the appropriate method for imputation. The chained equations approach was chosen because it is a very effective, flexible, and straightforward method to impute data. This method is based on a set of models adapted to the type of missing value; the values are filled first with random sampling, based only on the observed data, and then also based on already imputed data [49,50].

The imputation model was built by selecting the best missing data predictors among the available variables. The imputation model incorporated strong predictors of missing data (cognition, disability) and confounders (age, sex, education, physical activity). Moreover, outcomes were included in the imputation model (mortality, cancer, CVD), but they were not imputed. To optimise the imputed values, the data were ordered from lower to higher percentage of missing data before running the imputation, and a seed was set to allow reproducibility.

We performed 30 imputations to create 30 different data sets. Then, we ran 20 iterations by each of these 30 imputations, sufficient to achieve convergence of the Gibbs sampler. The imputations were assessed by hand (plausible values for imputed data compared to completed data) and by using graphical methods.

### Outcomes

We assessed 3 main outcomes: all-cause mortality, CVD, and cancer events. Mortality data linked to ELSA participants was provided by the National Health Service’s Central Registry, Southport, UK. For 68 participants, mortality was obtained from other sources (found during ELSA fieldwork or from participants’ relatives). Main causes of death were registered as CVD, cancer, diseases of the respiratory system, and other causes. CVD or cancer events were defined by self-report in waves 3–5. A CVD event could be myocardial infarction, heart failure, stroke, or CVD death. A cancer event could be cancer of any type, including cancer death. For each outcome separately, participants’ exposure time was calculated from the participant’s age at entry (wave 2 clinical examination: 2004–2005) to participant’s age at first event or final censoring (date of mortality assessment: February 2012). Participants lost to follow-up were right-censored at the midpoint between their last visit and the next one. For analysis of CVD and cancer incidence, respective prevalent cases at baseline were excluded.

### Definition of covariates/potential confounders

Smoker status was defined as never, previous, or current smoker. The maximum alcohol consumption per day was defined as 0, 1, 2, and >2 units/day. Body mass index (BMI) was defined as a continuous variable calculated as weight (kg)/height (m)^2^. Self-reported physical activity was defined as time spent in vigorous, moderate, low, and sedentary activity. Diabetes was defined through self-reported medical diagnosis or fasting glucose ≥7.0 mmol/L or glycated haemoglobin ≥6.5% [51]. Hypertension was defined from systolic or diastolic blood pressure ≥140 or ≥90 mm Hg, respectively, or self-reported high blood pressure medication [52]. Anaemia was defined as a measured haemoglobin level <13 g/dL (men) and <12 g/dL (women) [53]. Arthritis was self-reported diagnosis. Neuropsychiatric problems were self-reported diagnoses of: Alzheimer or Parkinson disease, dementia, or psychiatric problems. Cognition was evaluated with a total continuous cognitive index (memory and executive functions) [54]. Self-rated health was defined as excellent, very good, good, fair, or poor. Quality of life was evaluated with the 19-item scale control, autonomy, pleasure, and self-realization (CASP-19) questionnaire [55]. Depression symptoms were assessed with the 8-item Centre for Epidemiologic Study Depression Scale, with cutoff ≥4 points [56].

### Statistical analysis

We performed two parallel statistical analyses. The first was a continuous analysis with frailty scores rescaled to the range 0 (no frailty) to 1 (frailty). The second was a categorical analysis of frailty scores using cutoffs when they were defined.

All data analyses were carried out in R version 3.3.0 using packages ‘Mice’, ‘lattice’, ‘Survival’, mitml’, and ‘survC1’. A *p*-value of less than 0.05 was considered statistically significant.

#### Survival analysis

Cox proportional hazards models were fitted for each outcome and independently for each frailty score as a continuous variable. Where a published cutoff level to define frailty was available, an additional model was run on the binary or categorical frailty classification.

For each outcome (all-cause mortality, CVD, and cancer events), 4 models were fitted with progressive levels of adjustment (0–3): model 0: frailty score; model 1: model 0 + sex; model 2: model 1 + smoking status and alcohol consumption; and model 3: model 2 + physical activity, BMI, diabetes, hypertension, CVD, cancer, anaemia, chronic obstructive pulmonary disease (COPD), arthritis, neuropsychiatric problems, depression, cognition, and self-rated health and quality of life. The covariates in each model were chosen because all of them could potentially be confounders, affecting the outcome and/or the exposure. To avoid collinearity issues, the covariates of model 3 were tailored to each frailty score, excluding covariates that were an underlying variable of the score or a highly correlated variable. For CVD and cancer models, CVD and cancer were excluded as covariates (see S1 Table).

The proportional hazards assumption was checked by adding a time–covariate interaction in the model. The interaction term was retained in the model if significant [57]. The Cox models were fitted in 30 imputed data sets and the results, including calculated 95% confidence intervals, were pooled according to Rubin’s rules [58].

The discrimination ability was assessed with Harrell’s C statistic [9] using a calendar time to event scale. Three basic adjusted models: model 1 = age and sex; model 2 = model 1 + age, sex, smoking status, and alcohol; model 3 = model 2 + physical activity, BMI, diabetes, hypertension, CVD, cancer, anaemia, COPD, arthritis, neuropsychiatric problems, depression, cognition, and self-rated health and quality of life were calculated for each outcome. Each frailty score was added to each of these models and improvement of the predictive ability was assessed by evaluating whether the C statistic of the model with the score was significantly higher than in the respective base model. Results are expressed as the difference in C statistics (delta C with 95% confidence intervals) of each model, including a score and its respective base model.

#### Sensitivity analysis

We performed a sensitivity analysis by excluding all events that occurred during the first year of follow-up with the objective of assessing if pre-existing disease near the date of enrolling could bias the results. For all-cause mortality, all analyses were also performed stratified by sex and age (>70/≤70 years).

This study is reported as per the Strengthening the Reporting of Observational Studies in Epidemiology (STROBE) guidelines (S1 Text).

## Results

Table 2 shows the baseline characteristics of the participants included in the analysis.

**Table 2:** Baseline summary characteristics of 5,294 participants in ELSA wave 2 (2004–2005).

Mean (SD), age (years)	71.2 (8.0)
No. (%) men	2,377 (44.9%)
Mean (SD), BMI, (kg/m^2^)^1^	27.8 (4.8)
No. (%) by weight (underweight/normal/overweight/obesity)^1^^,^^2^	148 (2.8%)/1,341 (25.3%)/2,276 (43.0%)/1,529 (28.9%)
No. (%) by smoking status (current/former/never)	650 (12.3%)/2,738 (51.7%)/1,906 (36.0%)
No. (%) by physical activity (sedentary/low/moderate/vigorous)^1^^,^^3^	388 (7.3%)/1,440 (27.2%)/2,624 (49.6%)/842 (15.9%)
Mean (SD), blood glucose level (mmol/L)^1^	5.3 (1.5)
Mean (SD), blood glycated haemoglobin level (%)^1^	5.7 (0.8)
No. (%) with diabetes^1^^,^^4^	688 (13.0%)
Mean (SD), systolic/diastolic blood pressure (mm Hg)^1^	137.4 (19.2)/73.9 (11.2)
No (%) with hypertension^1^^,^^5^	2,733 (51.6%)
Mean (SD), total cholesterol (mmol/L)^1^	5.8 (1.2)
Mean (SD), LDL cholesterol (mmol/L)^1^	3.5 (1.0)
Mean (SD), HDL cholesterol (mmol/L)^1^	1.5 (0.4)
Mean (SD), triglyceride (mmol/L)^1^	1.8 (1.1)
No. (%) of dyslipidemia^1^^,^^6^	2,171 (41.0%)
No. (%) of CVD^7^	726 (13.7%)
No. (%) of cancer	490 (9.3%)
No. (%) of anaemia^1^^,^^8^	390 (7.4%)
No. (%) of lung disease	1,000 (18.9%)
No. (%) of arthritis	2,276 (43%)
No. (%) with depression symptoms^1^^,^^9^	1,694 (32%)
No. (%) by self-rated health (poor/fair/good/very good/excellent)^1^	401(7.6%)/1,167(22.0%)/1,756 (33.2%)/1,384(26.1%)/586 (11.1%)
Mean (SD), cognitive index (pp)^1^^,^^10^	27.1 (6.6)

^1^Imputed data: when data were imputed, SDs were calculated according to Rubin's rules.

^2^Underweight: BMI < 20; normal weight: BMI ≥ 20 and < 25; overweight: BMI ≥ 25 and < 30; obesity = BMI > 30 kg/m^2^.

^3^Self-reported frequency of at least once a week of mild/moderate/vigorous activity.

^4^Diabetes defined as self-reported, or fasting glucose ≥7.0 mmol/L, or glycated haemoglobin ≥6.5%.

^5^Hypertension defined as systolic ≥140 or diastolic blood pressure ≥90 mm Hg or taking antihypertensive medication.

^6^Dyslipidemia defined as total cholesterol >6.2 mmol or taking medication.

^7^CVD defined as self-reported myocardial infarction, stroke, or congestive heart disease.

^8^Haemoglobin lower than 13 g/dL in men and 12 g/dL in women.

^9^Depression defined with ≥4 out of the 8-item version of the Center for Epidemiological Studies-Depression Scale.

^10^Sum of memory and executive indices; values range from 0 (worst) to 50 (best).

**Abbreviations:** CVD, cardiovascular disease; ELSA, English Longitudinal Study of Ageing; HDL, high-density lipoprotein; LDL, low-density lipoprotein; No., number; pp, per point.

From 9,432 participants in wave 2 of ELSA, 5,294 (44.9% men) fulfilled the inclusion criteria. Mean age was 71.2 (SD: 8.0) years. The prevalence of CVD and cancer at baseline were 13.7% and 9.3%, respectively. Data from 4,554 participants free of CVD and 4,792 participants free of cancer at baseline were analysed in the respective incidence analyses.

The median follow-up times (Interquartile range) for mortality, CVD, and cancer outcomes were 7.25 (7.00–7.42), 5.83 (5.33–6.08), and 5.83 (5.17–6.08) years, respectively. The numbers of events were 1,144 deaths, 373 incident CVD events, and 425 incident cancer events, translating into a crude mortality rate of 326/10,000 person-years and an incidence rate of 167/10,000 and 184/10,000 person-years for CVD and cancer incidence, respectively. Main causes of death were registered as cancer (32.5%), CVD (35.1%), respiratory (14.8%), and other (17.6%).

For the majority of cases, the proportion hazard assumption was not proved. Therefore, all figures and tables show hazard ratios (HRs) at the median follow-up time (3.5 years for mortality and 2.5 years for CVD and cancer events).

### All-cause mortality events

**Fig 1:**
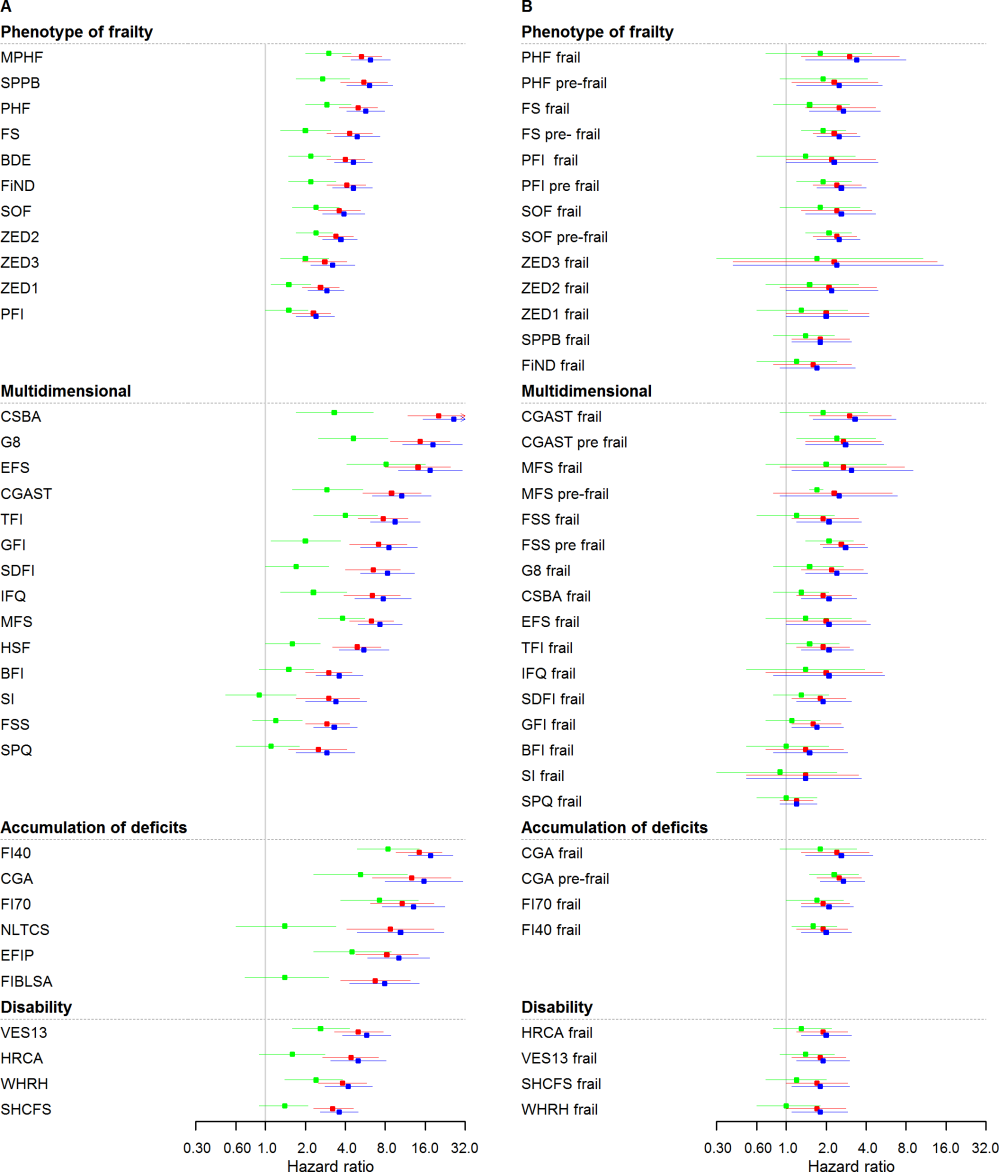
Mortality HRs of frailty scores (*n* = 5,294): Continuous and cutoff analysis. (A) Left panel: continuous anaylsis; (B) right panel: categorical analysis. Models were fitted using age as timescale, with time 0 = age at entry of study and time 1 = age at event or censoring date. Model 1 in blue: adjusted by sex. Model 2 in red: Model 1 + smoking status, alcohol, and alcohol consumption. Model 3 in green: Model 2 + physical activity, BMI, diabetes, hypertension, cardiovascular, cancer, anemia, COPD, arthritis, neuropsychiatric problems, depression, cognition, and self-rated health and quality of life. HRs were at 3.5 years (median follow-up for mortality). BDE, Beaver Dam Eye Study Index; BFI, Brief Frailty Index; BMI, body mass index; CGA, Comprehensive Geriatric Assessment; CGAST, Comprehensive Geriatric Assessment Screening Tests; COPD, chronic obstructive pulmonary disease; CSBA, Conselice Study of Brain Aging Score; EFIP, Evaluative Frailty Index for Physical Activity; EFS, Edmonton Frail Scale; FI40, 40-item Frailty Index; FI70, 70-item Frailty Index (SHARE); FIBLSA, Frailty Index Beijing Longitudinal Study of Ageing; FiND, Frail Non-Disabled Questionnaire; FS, Frail Scale; FSS, Frailty Staging System; G8, G-8 Geriatric Screening Tool; GFI, Groningen Frailty Indicator; HR, hazard ratio; HRCA, Hebrew Rehabilitation Center for Aged Vulnerability Index; HSF, Health Status Form; IFQ, Inter-Frail Questionnaire; MFS, Modified Frailty Score; MPHF, Modified Phenotype of Frailty; NLTCS, Long Term Care Survey Frailty Index; PFI, Physical Frailty Index; PHF, Phenotype of Frailty; SDFI, Static/Dynamic Frailty Index; SHCFS, Canadian Study of Health and Aging Clinical Frailty Scale; SI, Screening Instrument; SOF, Study of Osteoporotic Fractures; SPPB, Short Physical Performance Battery; SPQ, Sherbrooke Postal Questionnaire; TFI, Tilburg Frailty Indicator; VES13, Vulnerable Elders Survey; WHRH, WHOAFC and self-reported health; ZED1, ZutPhen Elderly Study (Physical Activity and Low Energy); ZED2, ZutPhen Elderly Study (Physical Activity and Weight Loss); ZED3, ZutPhen Elderly Study (Physical Activity and Low BMI).

Fig 1A and Table 3 show all-cause mortality HRs for frailty scores calculated at median time follow-up (3.5 years) and analysed as continuous variables at different levels of adjustment. The strength of the association between frailty scores and mortality ranged from an HR of 2.4 (95% CI: 1.7–3.3) to 26.2 (95% CI: 15.4–44.5) for those with the highest possible frailty state (rescaled to 1) to the lowest possible frailty state (rescaled to 0), with adjustment for sex. Adjustments in model 2 slightly attenuated associations for all scores, while retaining statistical significance in all cases. HRs for model 2 ranged from 2.3 (95% CI: 1.6–3.1) to 20.2 (95% CI: 11.8–34.5). Adjustments in model 3 attenuated associations for all scores, retaining statistical significance in 27 out of 35 cases. HRs for model 3 ranged from 0.9 (95% CI: 0.5–1.7) to 8.4 (95% CI: 4.9–14.4).

**Table 3:** Mortality HRs of frailty scores (*n* = 5,294) calculated at median time follow-up (3.5 years).

**Continuous analysis**					**Cutoff analysis**				
	HR (95% CI)	HR (95% CI)	HR (95% CI)	HR (95% CI)		HR (95% CI)	HR (95% CI)	HR (95% CI)	HR (95% CI)
Frailty Score	Model 0^1^	Model 1^2^	Model 2^3^	Model 3^4^	Frailty Score	Model 0^1^	Model 1^2^	Model 2^3^	Model 3^4^
**Phenotype of frailty approach**									
MPHF	5.4 (3.9–7.6)	6.2 (4.4–8.7)	5.3 (3.8–7.5)	3.0 (2.0–4.4)	PHF frail	3.0 (1.3–7.2)	3.4 (1.4–8.0)	3.0 (1.3–7.1)	1.8 (0.7–4.4)
SPPB	5.0 (3.3–7.4)	6.1 (4.1–9.1)	5.5 (3.7–8.3)	2.7 (1.7–4.3)	PHF pre-frail	2.3 (1.1–5.0)	2.5 (1.2–5.3)	2.3 (1.1–4.9)	1.9 (0.9–4.1)
PHF	5.1 (3.6–7.1)	5.7 (4.1–7.9)	5.0 (3.6–7.0)	2.9 (2.0–4.4)	FS frail	2.5 (1.4–4.7)	2.7 (1.5–5.1)	2.5 (1.4–4.7)	1.5 (0.8–3.0)
FS	4.2 (2.8–6.2)	4.9 (3.3–7.3)	4.3 (2.9–6.4)	2.0 (1.3–3.1)	FS pre-frail	2.3 (1.6–3.4)	2.5 (1.7–3.6)	2.3 (1.6–3.4)	1.9 (1.3–2.8)
BDE	1.9 (1.4–2.7)	4.6 (3.3–6.4)	4.0 (2.9–5.6)	2.2 (1.5–3.1)	PFI frail	2.0 (0.9–4.5)	2.3 (1.0–4.9)	2.2 (1.0–4.7)	1.4 (0.6–3.3)
FiND	3.9 (2.8–5.5)	4.6 (3.2–6.4)	4.1 (2.9–5.7)	2.2 (1.5–3.4)	PFI pre-frail	2.4 (1.6–3.7)	2.6 (1.7–4.0)	2.4 (1.6–3.7)	1.9 (1.2–3.1)
SOF	3.5 (2.4–5.0)	3.9 (2.7–5.6)	3.6 (2.5–5.2)	2.4 (1.6–3.6)	SOF frail	2.4 (1.3–4.3)	2.6 (1.4–4.7)	2.4 (1.3–4.4)	1.8 (0.9–3.6)
ZED2	3.3 (2.5–4.5)	3.7 (2.7–4.9)	3.4 (2.5–4.6)	2.4 (1.7–3.2)	SOF pre-frail	2.3 (1.6–3.4)	2.5 (1.7–3.6)	2.4 (1.6–3.4)	2.1 (1.4–3.1)
ZED3	2.6 (1.8–3.9)	3.2 (2.2–4.7)	2.8 (1.9–4.1)	2.0 (1.3–3.0)	ZED3 frail	2.3 (0.3–14.8)	2.4 (0.4–15.2)	2.3 (0.4–13.7)	1.7 (0.3–10.7)
ZED1	2.5 (1.9–3.4)	2.9 (2.1–3.9)	2.6 (1.9–3.6)	1.5 (1.1–2.2)	ZED2 frail	2.1 (0.9–4.6)	2.2 (1.0–4.9)	2.1 (0.9–4.8)	1.5 (0.7–3.5)
PFI	2.2 (1.6–3.0)	2.4 (1.7–3.3)	2.3 (1.6–3.1)	1.5 (1.0–2.1)	ZED1 frail	1.9 (0.9–3.9)	2.0 (1.0–4.2)	2.0 (1.0–4.2)	1.3 (0.6–2.9)
					SPPB frail	1.8 (1.1–3.0)	1.8 (1.1–3.1)	1.8 (1.1–3.0)	1.4 (0.8–2.3)
					FiND frail	1.6 (0.8–3.1)	1.7 (0.9–3.3)	1.6 (0.8–3.1)	1.2 (0.6–2.4)
**Multidimensional approach**									
CSBA	33.4 (20.0–55.8)	26.2 (15.4–44.5)	20.2 (11.8–34.5)	3.3 (1.7–6.5)	CGAST frail	3.0 (1.5–6.2)	3.3 (1.6–6.7)	3.0 (1.5–6.2)	1.9 (0.9–4.1)
G8	13.5 (8.1–22.6)	18.2 (10.8–30.4)	14.6 (8.7–24.6)	4.6 (2.5–8.4)	CGAST pre-frail	2.7 (1.4–5.2)	2.8 (1.4–5.4)	2.7 (1.4–5.2)	2.4 (1.2–4.7)
EFS	13.5 (7.7–23.5)	17.4 (10.0–30.3)	14.1 (8.0–24.8)	8.1 (4.1–16.0)	MFS frail	1.6 (0.6–4.7)	3.1 (1.1–9.0)	2.7 (0.9–7.8)	2.0 (0.7–5.7)
CGAST	8.3 (5.0–13.8)	10.6 (6.4–17.6)	8.9 (5.4–14.9)	2.9 (1.6–5.4)	MFS pre-frail	1.4 (0.5–3.8)	2.5 (0.9–6.9)	2.3 (0.8–6.3)	1.7 (1.5–1.9)
TFI	6.7 (4.4–10.2)	9.5 (6.2–14.6)	7.7 (5.0–11.8)	4.0 (2.3–7.0)	FSS frail	2.0 (1.1–3.5)	2.1 (1.2–3.7)	1.9 (1.1–3.5)	1.2 (0.6–2.3)
GFI	6.7 (4.1–10.9)	8.5 (5.2–13.9)	7.1 (4.3–11.6)	2.0 (1.1–3.7)	FSS pre-frail	2.7 (1.8–4.0)	2.8 (1.9–4.1)	2.6 (1.8–3.9)	2.1 (1.4–3.2)
SDFI	4.7 (3.0–7.4)	8.3 (5.2–13.2)	6.5 (4.0–10.4)	1.7 (1.0–3.0)	G8 frail	2.3 (1.3–3.8)	2.4 (1.4–4.1)	2.2 (1.3–3.8)	1.5 (0.8–2.7)
IFQ	5.8 (3.6–9.4)	7.7 (4.7–12.5)	6.4 (3.9–10.4)	2.3 (1.3–4.1)	CSBA frail	2.3 (1.5–3.7)	2.1 (1.3–3.4)	1.9 (1.2–3.1)	1.3 (0.8–2.1)
MFS	6.5 (4.5–9.5)	7.3 (5.0–10.7)	6.3 (4.3–9.2)	3.8 (2.5–5.6)	EFS frail	1.9 (0.9–4.0)	2.1 (1.0–4.3)	2.0 (1.0–4.0)	1.4 (0.7–3.1)
HSF	5.1 (3.3–7.8)	5.5 (3.6–8.5)	4.9 (3.2–7.4)	1.6 (1.0–2.6)	TFI frail	1.9 (1.2–3.0)	2.1 (1.3–3.2)	1.9 (1.2–3.0)	1.5 (1.0–2.5)
BFI	2.6 (1.7–3.9)	3.6 (2.4–5.4)	3.0 (2.0–4.5)	1.5 (0.9–2.3)	IFQ frail	1.9 (0.7–5.1)	2.1 (0.8–5.5)	2.0 (0.7–5.3)	1.4 (0.5–3.9)
SI	2.6 (1.5–4.5)	3.4 (2.0–5.8)	3.0 (1.7–5.1)	0.9 (0.5–1.7)	SDFI frail	1.7 (1.1–2.7)	1.9 (1.2–3.1)	1.8 (1.1–2.8)	1.3 (0.8–2.1)
FSS	3.0 (2.1–4.3)	3.3 (2.3–4.9)	2.9 (2.0–4.3)	1.2 (0.8–1.9)	GFI frail	1.6 (1.0–2.5)	1.7 (1.1–2.7)	1.6 (1.1–2.6)	1.1 (0.7–1.8)
SPQ	2.2 (1.4–3.7)	2.9 (1.7–4.7)	2.5 (1.5–4.1)	1.1 (0.6–1.8)	BFI frail	1.3 (0.7–2.6)	1.5 (0.8–2.9)	1.4 (0.7–2.7)	1 (0.5–2.1)
					SI frail	1.3 (0.5–3.4)	1.4 (0.5–3.7)	1.4 (0.5–3.5)	0.9 (0.3–2.4)
					SPQ frail	1.2 (0.8–2.0)	1.2 (0.9–1.7)	1.2 (1.0–1.4)	1.0 (0.6–1.7)
**Accumulation of deficits approach**									
FI40	10.6 (6.1–18.3)	17.5 (11.9–25.8)	14.4 (9.6–21.4)	8.4 (4.9–14.4)	CGA frail	2.2 (1.3–3.9)	2.6 (1.4–4.5)	2.4 (1.3–4.2)	1.8 (0.9–3.4)
CGA	9.7 (5.0–19.0)	15.6 (8.0–30.5)	12.6 (6.4–24.9)	5.2 (2.3–11.7)	CGA pre-frail	2.4 (1.6–3.6)	2.7 (1.8–3.9)	2.5 (1.7–3.7)	2.3 (1.5–3.5)
FI70	8.7 (5.1–14.8)	13.0 (7.6–22.4)	10.7 (6.2–18.5)	7.2 (3.7–14.2)	FI70 frail	1.9 (1.2–2.9)	2.1 (1.3–3.2)	1.9 (1.3–3.0)	1.7 (1.0–2.7)
NLTCS	9.0 (4.2–19.0)	10.4 (4.9–22.1)	8.7 (4.1–18.6)	1.4 (0.6–3.4)	FI40 frail	1.8 (1.2–2.8)	2.0 (1.3–3.1)	1.9 (1.2–2.9)	1.6 (1.1–2.4)
EFIP	7.7 (4.5–13.2)	10.1 (5.9–17.3)	8.2 (4.8–14.2)	4.5 (2.3–8.9)					
FIBLSA	6.2 (3.4–11.4)	7.9 (4.3–14.4)	6.7 (3.7–12.3)	1.4 (0.7–3.0)					
**Disability approach**									
VES13	4.6 (3.0–7.0)	5.8 (3.8–8.8)	5.0 (3.3–7.7)	2.6 (1.6–4.3)	HRCA frail	1.7 (1.1–2.7)	2.0 (1.3–3.1)	1.9 (1.2–2.9)	1.3 (0.8–2.2)
HRCA	3.9 (2.4–6.4)	5.0 (3.1–8.1)	4.4 (2.7–7.1)	1.6 (0.9–2.8)	VES13 frail	1.7 (1.1–2.7)	1.9 (1.2–3.0)	1.8 (1.1–2.8)	1.4 (0.9–2.3)
WHRH	3.5 (2.3–5.4)	4.2 (2.8–6.4)	3.8 (2.5–5.8)	2.4 (1.4–3.8)	SHCFS frail	1.7 (1.0–2.8)	1.8 (1.1–3.0)	1.7 (1.0–2.9)	1.2 (0.7–2.0)
SHCFS	3.2 (2.3–4.5)	3.6 (2.6–5.0)	3.2 (2.3–4.6)	1.4 (0.9–2.1)	WHRH frail	1.7 (1.0–2.7)	1.8 (1.1–2.9)	1.7 (1.0–2.8)	1.0 (0.6–1.8)

^1^Model 0 = Crude models.

^2^Model 1 = HR adjusted by sex.

^3^Model 2 = Model 1 + smoking status and alcohol consumption.

^4^Model 3 = Model 2 + physical activity, BMI, diabetes, hypertension, cardiovascular, cancer, anemia, COPD, arthritis, neuropsychiatric problems, depression, cognition, and self-rated health and quality of life. Models were fitted using age as timescale, with time 0 = age at entry of study and time 1 = age at event or censoring date.

**Abbreviations:** BDE, Beaver Dam Eye Study Index; BFI, Brief Frailty Index; BMI, body mass index; CGA, Comprehensive Geriatric Assessment; CGAST, Comprehensive Geriatric Assessment Screening Tests; COPD, chronic obstructive pulmonary disease; CSBA, Conselice Study of Brain Aging Score; EFIP, Evaluative Frailty Index for Physical Activity; EFS, Edmonton Frail Scale; FI40, 40-item Frailty Index; FI70, 70-item Frailty Index (SHARE); FIBLSA, Frailty Index Beijing Longitudinal Study of Ageing; FiND, Frail Non-Disabled Questionnaire; FS, Frail Scale; FSS, Frailty Staging System; G8, G-8 Geriatric Screening Tool; GFI, Groningen Frailty Indicator; HR, hazard ratio; HRCA, Hebrew Rehabilitation Center for Aged Vulnerability Index; HSF, Health Status Form; IFQ, Inter-Frail Questionnaire; MFS, Modified Frailty Score; MPHF, Modified Phenotype of Frailty; NLTCS, Long Term Care Survey Frailty Index; PFI, Physical Frailty Index; PHF, Phenotype of Frailty; SDFI, Static/Dynamic Frailty Index; SHCFS, Canadian Study of Health and Aging Clinical Frailty Scale; SI, Screening Instrument; SOF, Study of Osteoporotic Fractures; SPPB, Short Physical Performance Battery; SPQ, Sherbrooke Postal Questionnaire; TFI, Tilburg Frailty Indicator; VES13, Vulnerable Elders Survey; WHRH, WHOAFC and self-reported health; ZED1, ZutPhen Elderly Study (Physical Activity and Low Energy); ZED2, ZutPhen Elderly Study (Physical Activity and Weight Loss); ZED3, ZutPhen Elderly Study (Physical Activity and Low BMI).

Fig 1B and Table 3 illustrate the same analysis using categorical variables (frailty status). In sex-adjusted models, HRs ranged from 1.2 (95% CI: 0.9–1.7) to 3.4 (95% CI: 1.4–8.0), with 30 out of 37 cases showing a statistically significant association. Adjustments in model 2 attenuated associations, while retaining statistical significance in 28 out of 37 cases. HRs for model 2 ranged from 1.2 (95% CI: 1.0–1.4) to 3.0 (95% CI: 1.5–6.2). Adjustments in model 3 attenuated associations for all scores, retaining statistical significance in 10 out of 37 cases. HRs for model 3 ranged from 0.9 (95% CI: 0.3–2.4) to 2.4 (95% CI: 1.2–4.7). S2 and S3 Tables show HRs for total mortality assessed in yearly intervals, with continuous and categorical analysis, respectively.

### Cardiovascular events

**Fig 2:**
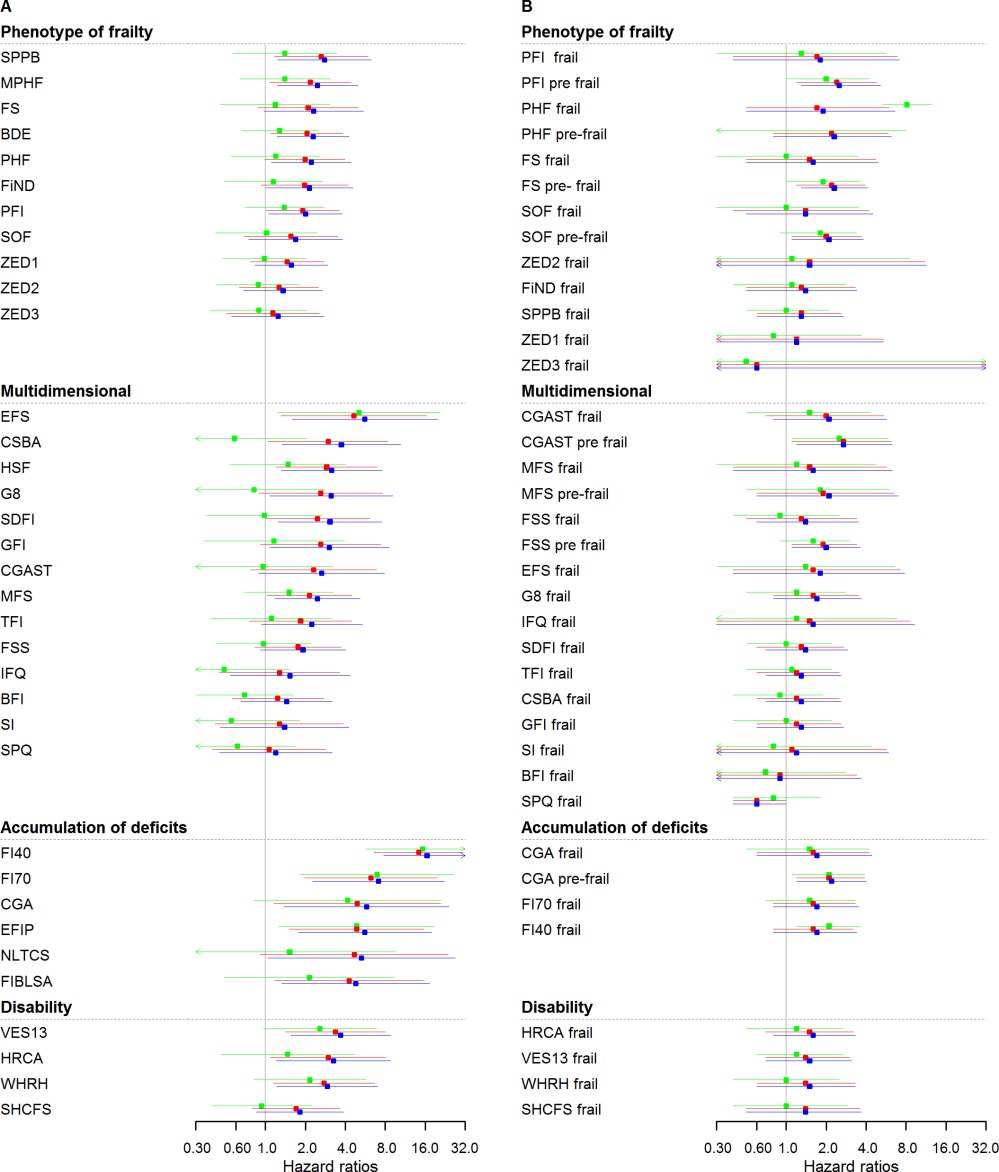
Cardiovascular HRs of frailty scores (*n* = 4,554): Continuous and cutoff analysis. (A) Left panel: continuous anaylsis; (B) right panel: categorical analysis. Models were fitted using age as timescale, with time 0 = age at entry of study and time 1 = age at event or censoring date. Model 1 in blue: adjusted by sex. Model 2 in red: Model 1 + smoking status, alcohol, and alcohol consumption. Model 3 in green: Model 2 + physical activity, BMI, diabetes, hypertension, cancer, anemia, COPD, arthritis, neuropsychiatric problems, depression, cognition, and self-rated health and quality of life. HRs were at 2.5 years (median follow-up for CVD events). BDE, Beaver Dam Eye Study Index; BFI, Brief Frailty Index; BMI, body mass index; CGA, Comprehensive Geriatric Assessment; CGAST, Comprehensive Geriatric Assessment Screening Tests; COPD, chronic obstructive pulmonary disease; CSBA, Conselice Study of Brain Aging Score; CVD, cardiovascular disease; EFIP, Evaluative Frailty Index for Physical Activity; EFS, Edmonton Frail Scale; FI40, 40-item Frailty Index; FI70, 70-item Frailty Index (SHARE); FIBLSA, Frailty Index Beijing Longitudinal Study of Ageing; FiND, Frail Non-Disabled Questionnaire; FS, Frail Scale; FSS, Frailty Staging System; G8, G-8 Geriatric Screening Tool; GFI, Groningen Frailty Indicator; HR, hazard ratio; HRCA, Hebrew Rehabilitation Center for Aged Vulnerability Index; HSF, Health Status Form; IFQ, Inter-Frail Questionnaire; MFS, Modified Frailty Score; MPHF, Modified Phenotype of Frailty; NLTCS, Long Term Care Survey Frailty Index, PFI, Physical Frailty Index; PHF, Phenotype of Frailty; SDFI, Static/Dynamic Frailty Index; SHCFS, Canadian Study of Health and Aging Clinical Frailty Scale; SI, Screening Instrument; SOF, Study of Osteoporotic Fractures; SPPB, Short Physical Performance Battery; SPQ, Sherbrooke Postal Questionnaire; TFI, Tilburg Frailty Indicator; VES13, Vulnerable Elders Survey; WHRH, WHOAFC and self-reported health; ZED1, ZutPhen Elderly Study (Physical Activity and Low Energy); ZED2, ZutPhen Elderly Study (Physical Activity and Weight Loss); ZED3, ZutPhen Elderly Study (Physical Activity and Low BMI).

Fig 2A and S4 Table show HRs for incident CVD for frailty scores analysed as continuous variables. Twenty-three out of thirty-five scores showed a statistically significant association in sex-adjusted models (model 1), ranging from 1.2 (95% CI: 0.5–3.2) to 16.5 (95% CI: 7.8–35.0). Adjustments in model 2 attenuated associations for all scores, retaining statistical significance in 18 out of 35 cases. Further adjustment with model 3 further attenuated associations for all scores, retaining statistical significance in 5 out of 35 cases. The strongest and more stable associations after adjustment with CVD events were seen for scores from the “accumulation of deficits approach” group.

Fig 2B and S4 Table show the analysis performed for incident CVD based on the categorical frailty definitions. Only 6 out of 37 HRs were statistically significant and ranged from 0.6 (95% CI: 0.4–1.0) to 2.7 (1.2–6.3) in sex-adjusted models. The effect of adjustment was a slight attenuation of the associations. S5 and S6 Tables show HR for cardiovascular events assessed in yearly intervals with continuous and categorical analysis, respectively.

### Cancer events

**Fig 3:**
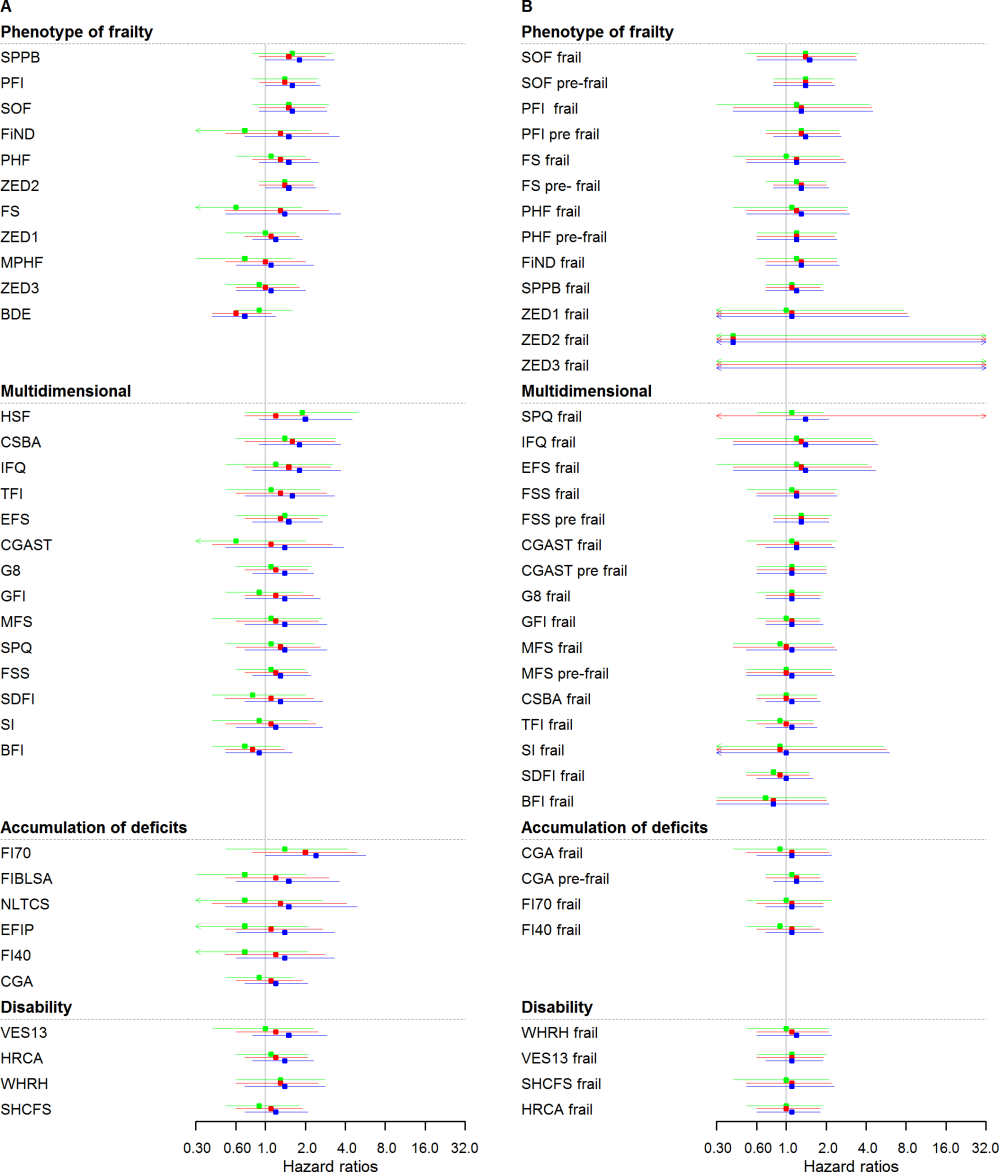
Cancer HRs of frailty scores (*n* = 4,792): Continuous and cutoff analysis. (A) Left panel: continuous analysis; (B) right panel: categorical analysis. Models were fitted using age as timescale, with time 0 = age at entry of study and time 1 = age at event or censoring date. Model 1 in blue: adjusted by sex. Model 2 in red: Model 1 + smoking status, alcohol, and alcohol consumption. Model 3 in green: Model 2 + physical activity, BMI, diabetes, hypertension, cardiovascular, anaemia, COPD, arthritis, neuropsychiatric problems, depression, cognition, and self-rated health and quality of life. HRs were at 2.5 years (median follow-up for cancer events). BDE, Beaver Dam Eye Study Index; BFI, Brief Frailty Index; BMI, body mass index; CGA, Comprehensive Geriatric Assessment; CGAST, Comprehensive Geriatric Assessment Screening Tests; COPD, chronic obstructive pulmonary disease; CSBA, Conselice Study of Brain Aging Score; CVD, cardiovascular disease; EFIP, Evaluative Frailty Index for Physical Activity; EFS, Edmonton Frail Scale; FI40, 40-item Frailty Index; FI70, 70-item Frailty Index (SHARE); FIBLSA, Frailty Index Beijing Longitudinal Study of Ageing; FiND, Frail Non-Disabled Questionnaire; FS, Frail Scale; FSS, Frailty Staging System; G8, G-8 Geriatric Screening Tool; GFI, Groningen Frailty Indicator; HR, hazard ratio; HRCA, Hebrew Rehabilitation Center for Aged Vulnerability Index; HSF, Health Status Form; IFQ, Inter-Frail Questionnaire; MFS, Modified Frailty Score; MPHF, Modified Phenotype of Frailty; NLTCS, Long Term Care Survey Frailty Index; PFI, Physical Frailty Index; PHF, Phenotype of Frailty; SDFI, Static/Dynamic Frailty Index; SHCFS, Canadian Study of Health and Aging Clinical Frailty Scale; SI, Screening Instrument; SOF, Study of Osteoporotic Fractures; SPPB, Short Physical Performance Battery; SPQ, Sherbrooke Postal Questionnaire; TFI, Tilburg Frailty Indicator; VES13, Vulnerable Elders Survey; WHRH, WHOAFC and self-reported health; ZED1, ZutPhen Elderly Study (Physical Activity and Low Energy); ZED2, ZutPhen Elderly Study (Physical Activity and Weight Loss); ZED3, ZutPhen Elderly Study (Physical Activity and Low BMI).

Fig 3 and S7 Table show HRs for incident cancer. Analyses based on continuous scores (Fig 3A) yielded HRs for cancer ranging between 0.7 (95% CI: 0.4–1.2) and 2.4 (95% CI: 1.0–5.7), while most associations (31 out of 35) did not reach statistical significance in sex-adjusted models. Further adjustment (models 2 and 3) attenuated associations for all scores, not retaining any statistical significance. Fig 3B and S7 Table show the results based on categorical frailty classifications, for which most associations did not reach statistical significance; also, with further adjustment (models 2 and 3), no score retained any statistical significance. S8 and S9 Tables show HRs for cancer events assessed in yearly intervals, with continuous and categorical analysis, respectively.

### Evaluation of discriminative ability

Table 4 shows the discriminative ability of frailty scores for all-cause mortality using Harrell’s C statistic. The improvement in prediction for each frailty score analysed as a continuous variable on top of a basic model consisting of age and sex ranged from 0.6% (95% CI: 0.2–0.9) to 3.1% (95% CI: 2.3–3.9) and was statistically significant for all scores. With model 2, improvement was significant in all cases and ranged from 0.4% (95% CI: 0.1–0.7) to 2.5% (95% CI: 1.7–3.2). With further adjusted model 3, improvement was significant in 33 out of 35 cases and ranged from 0.0 (95% CI: −0.4–0.3) to 0.9 (95% CI: 0.5–1.3).

**Table 4:** Discriminative assessment of mortality models using Harrell's C statistic (*n* = 5,294).

Continuous analysis				Cut-off analysis			
Frailty Score	Delta (*100) LCI; UCI with 95%CI^1^	Delta (*100) LCI; UCI with 95%CI^1^	Delta (*100) LCI; UCI with 95%CI^1^	Frailty Score	Delta (*100) LCI; UCI with 95%CI^1^	Delta (*100) LCI; UCI with 95%CI^1^	Delta (*100) LCI; UCI with 95%CI^1^
	Model 1	Model 2	Model 3		Model 1	Model 2	Model 3
**Basic models**	**74.3 (72.6–76.0)**^**2**^	**75.3 (73.8–76.9)**^**2**^	**79.1 (77.8–80.4)**	**Basic models**	**74.3 (72.6–76.0)**^**2**^	**75.3 (73.8–76.9)**^**2**^	**79.1 (77.8–80.4)**
**Phenotype of frailty approach**								
PHF	2.8 (2.0–3.7)	2.3 (1.6–2.9)	0.6 (0.3–0.9)	PHF frail	1.6 (1.1–2.2)	1.4 (0.9–1.9)	0.3 (0.1–0.5)
MPHF	2.8 (2.0–3.5)	2.2 (1.6–2.8)	0.5 (0.2–0.8)	PHF pre-frail	0.6 (0.3–1.0)	0.6 (0.2–1.0)	0.2 (0.0–0.3)
FiND	2.4 (1.7–3.1)	1.8 (1.2–2.4)	0.2 (0.0–0.4)	SOF frail	1.1 (0.6–1.6)	1.0 (0.6–1.4)	0.2 (0.0–0.3)
ZED2	2.3 (1.7–2.9)	1.9 (1.3–2.4)	0.6 (0.3–1.0)	SOF pre-frail	0.3 (0.1–0.6)	0.2 (0.0–0.4)	0.0 (−0.1–0.1)
FS	2.1 (1.4–2.7)	1.5 (1.0–2.1)	0.1 (0.0–0.3)	FS frail	1.0 (0.5–1.4)	0.8 (0.4–1.2)	0.0 (−0.1–0.1)
BDE	2.0 (1.2–2.8)	1.6 (1.0–2.1)	0.3 (0.0–0.6)	FS pre-frail	0.3 (0.1–0.6)	0.2 (0.0–0.4)	0.0 (0.0–0.1)
SOF	2.0 (1.4–2.6)	1.6 (1.1–2.1)	0.3 (0.1–0.6)	ZED1 frail	0.7 (0.4–1.1)	0.6 (0.2–1.0)	0.1 (0.0–0.1)
SPPB	2.0 (1.3–2.7)	1.6 (1.0–2.2)	0.3 (0.0–0.5)	PFI frail	0.4 (0.2–0.6)	0.4 (0.1–0.6)	0.0 (0.0–0.1)
ZED1	1.6 (1.0–2.2)	1.3 (0.8–1.7)	0.1 (0.0–0.2)	PFI pre-frail	0.7 (0.3–1.2)	0.5 (0.2–0.9)	0.1 (0.0–0.2)
PFI	1.5 (0.9–2.0)	1.1 (0.6–1.6)	0.2 (0.0–0.3)	SPPB frail	0.7 (0.3–1.2)	0.5 (0.2–0.9)	0.0 (−0.1–0.2)
ZED3	1.3 (0.8–1.8)	1.0 (0.5–1.4)	0.3 (0.1–0.5)	ZED2 frail	0.5 (0.2–0.9)	0.5 (0.2–0.8)	0.1 (0.0–0.2)
				FiND frail	0.4 (0.1–0.7)	0.3 (0.0–0.5)	0.0 (0.0–0.1)
				ZED3 frail	0.2 (0.0–0.5)	0.2 (0.0–0.4)	0.0 (0.0–0.1)
**Multidimensional approach**							
EFS	3.1 (2.3–3.9)	2.5 (1.7–3.2)	0.9 (0.5–1.3)	TFI frail	1.9 (1.3–2.6)	1.4 (0.9–2.0)	0.3 (0.1–0.6)
G8	2.9 (2.0–3.8)	2.3 (1.6–2.9)	0.4 (0.1–0.8)	CGAST frail	1.9 (1.1–2.6)	1.5 (0.9–2.0)	0.2 (0.0–0.3)
CGAST	2.7 (1.8–3.6)	2.1 (1.5–2.7)	0.3 (0.1–0.5)	G8 frail	1.6 (1.0–2.3)	1.2 (0.6–1.7)	0.0 (−0.3–0.4)
CSBA	2.5 (1.7–3.4)	2.0 (1.3–2.7)	0.1 (0.0–0.3)	CSBA frail	1.6 (1.0–2.2)	1.1 (0.6–1.6)	0.0 (−0.1–0.2)
TFI	2.4 (1.6–3.1)	1.8 (1.1–2.4)	0.4 (0.1–0.6)	SDFI frail	1.4 (0.8–1.9)	0.9 (0.4–1.3)	0.1 (−0.1–0.2)
MFS	2.3 (1.6–3.0)	1.8 (1.2–2.5)	0.6 (0.3–0.9)	MFS frail	1.3 (0.7–1.9)	1.0 (0.5–1.6)	0.3 (0.1–0.5)
HSF	2.1 (1.5–2.7)	1.7 (1.1–2.3)	0.1 (0.0–0.2)	MFS pre-frail	0.8 (0.4–1.2)	0.7 (0.3–1.0)	0.2 (0.0–0.4)
GFI	2.1 (1.4–2.7)	1.6 (1.0–2.1)	0.1 (0.0–0.2)	GFI frail	1.3 (0.7–1.8)	0.9 (0.5–1.3)	0.0 (−0.1–0.1)
SDFI	2.0 (1.3–2.6)	1.4 (0.9–1.9)	0.1 (0.0–0.2)	EFS frail	0.8 (0.3–1.3)	0.6 (0.3–1.0)	0.1 (0.0–0.3)
IFQ	1.8 (1.2–2.5)	1.4 (0.8–1.9)	0.2 (0.0–0.4)	CGAST pre-frail	0.6 (0.2–1.0)	0.5 (0.1–0.8)	0.0 (0.0–0.1)
FSS	1.3 (0.7–1.9)	1.0 (0.6–1.5)	0.0 (0.0–0.1)	SPQ frail	0.4 (0.1–0.8)	0.3 (0.0–0.5)	0.0 (0.0–0.1)
BFI	1.2 (0.6–1.7)	0.8 (0.5–1.2)	0.0 (0.0–0.1)	FSS frail	0.4 (0.1–0.7)	0.3 (0.1–0.6)	0.0 (−0.1–0.1)
SI	0.9 (0.5–1.4)	0.7 (0.3–1.1)	0.0 (0.0–0.0)	FSS pre-frail	0.4 (0.1–0.7)	0.2 (0.0–0.5)	0.1 (0.0–0.3)
SPQ	0.6 (0.2–0.9)	0.4 (0.1–0.7)	0.0 (0.0–0.1)	BFI frail	0.3 (0.1–0.6)	0.2 (0.0–0.4)	0.0 (0.0–0.0)
				IFQ frail	0.3 (0.0–0.5)	0.2 (0.0–0.4)	0.0 (0.0–0.1)
				SI frail	0.1 (0.0–0.3)	0.1 (0.0–0.3)	0.0 (−0.1–0.1)
**Accumulation of deficits approach**							
FI40	2.6 (1.8–3.5)	2.1 (1.4–2.7)	0.7 (0.4–1.0)	FI70 frail	2.1 (1.5–2.6)	1.6 (1.0–2.1)	0.6 (0.2–0.9)
FI70	2.5 (1.8–3.1)	1.9 (1.4–2.4)	0.7 (0.3–1.0)	FI40 frail	1.9 (1.3–2.4)	1.4 (0.8–2.0)	0.5 (0.2–0.8)
EFIP	2.0 (1.4–2.6)	1.5 (1.0–2.1)	0.4 (0.1–0.6)	CGA frail	1.2 (0.7–1.6)	0.9 (0.4–1.3)	0.2 (0.0–0.3)
CGA	1.9 (1.3–2.6)	1.5 (0.9–2.1)	0.4 (0.1–0.6)	CGA pre-frail	0.1 (0.0–0.2)	0.0 (-0.1–0.1)	0.0 (-0.1–0.1)
FIBLSA	1.6 (1.0–2.2)	1.2 (0.7–1.7)	0.0 (0.0–0.1)				
NLTCS	1.4 (0.9–2.0)	1.2 (0.7–1.6)	0.2 (0.0–0.4)				
**Disability approach**							
VES13	2.2 (1.5–2.9)	1.7 (1.2–2.3)	0.4 (0.1–0.6)		HRCA frail	1.7 (1.1–2.3)	1.3 (0.8–1.8)	0.1 (−0.1–0.3)
WHRH	1.8 (1.2–2.3)	1.4 (0.9–1.9)	0.0 (−0.4–0.3)		VES13 frail	1.5 (0.8–2.1)	1.1 (0.6–1.6)	0.2 (0.0–0.4)
SHCFS	1.8 (1.2–2.3)	1.4 (0.9–2.0)	0.3 (0.1–0.6)		SHCFS frail	1.1 (0.6–1.6)	0.9 (0.5–1.3)	0.2 (0.0–0.3)
HRCA	1.6 (1.2–2.1)	1.2 (0.8–1.7)	0.0 (−0.1–0.1)		WHRH frail	1.1 (0.5–1.7)	0.9 (0.4–1.3)	−0.3 (−0.6–0.0)

Model 1 = age and sex. Model 2 = model 1 + smoking status and maximum alcohol consumption. Model 3 = Model 2 + physical activity, BMI, diabetes, hypertension, cardiovascular, cancer, anaemia, COPD, arthritis, neuropsychiatric problems, depression, cognition, and self-rated health and quality of life.

^1^Delta = percent of improvement adding the frailty score to model.

^2^Harrel's C statistic of each model (lower confidence interval; upper confidence interval)*100.

**Abbreviations:** BDE, Beaver Dam Eye Study Index; BFI, Brief Frailty Index; BMI, body mass index; CGA, Comprehensive Geriatric Assessment; CGAST, Comprehensive Geriatric Assessment Screening Tests; COPD, chronic obstructive pulmonary disease; CSBA, Conselice Study of Brain Aging Score; EFIP, Evaluative Frailty Index for Physical Activity; EFS, Edmonton Frail Scale; FI40, 40-item Frailty Index; FI70, 70-item Frailty Index (SHARE); FIBLSA, Frailty Index Beijing Longitudinal Study of Ageing; FiND, Frail Non-Disabled Questionnaire; FS, Frail Scale; FSS, Frailty Staging System; G8, G-8 Geriatric Screening Tool; GFI, Groningen Frailty Indicator; HRCA, Hebrew Rehabilitation Center for Aged Vulnerability Index; HSF, Health Status Form; IFQ, Inter-Frail Questionnaire; LCI, lower confidence interval; MFS, Modified Frailty Score; MPHF, Modified Phenotype of Frailty; NLTCS, Long Term Care Survey Frailty Index; PFI, Physical Frailty Index; PHF, Phenotype of Frailty; SDFI, Static/Dynamic Frailty Index; SHCFS, Canadian Study of Health and Aging Clinical Frailty Scale; SI, Screening Instrument; SOF, Study of Osteoporotic Fractures; SPPB, Short Physical Performance Battery; SPQ, Sherbrooke Postal Questionnaire; TFI, Tilburg Frailty Indicator; UCI, upper confidence interval; VES13, Vulnerable Elders Survey; WHRH, WHOAFC & self-reported health; ZED1, ZutPhen Elderly Study (Physical Activity and Low Energy); ZED2, ZutPhen Elderly Study (Physical Activity and Weight Loss); ZED3, ZutPhen Elderly Study (Physical Activity and Low BMI).

Analyses adding frailty categories to the age and sex basic model gave improvements ranging from 0.1% (95% CI: 0.0–0.2) to 2.1% (95% CI: 1.5–2.6), with all scores showing statistically significant improvement. In most cases, when the predictive value of the different scores was assessed over and above basic models 2, the improvement was attenuated; in most cases, it was also statistically significant.

The C statistic of the basic model for CVD events based only on age and sex was 70.1 (95% CI: 65.7–74.4). None of the continuous scores added predictive performance to this model at a statistically significant level. In analyses of frailty categories, only the G-8 Geriatric Screening Tool (G8) score added statistically significant predictive value (delta C: 1.6 [95% CI: 0.4–2.8]) (S10 Table).

For cancer events, the C statistic of all three basic models was below 60, and all deltas were nonsignificant both in continuous and categorical analyses (S11 Table).

### Sensitivity analysis

In sensitivity analyses excluding all events occurring the first year, we observed very similar results compared to those obtained with the total sample, although the strength of the associations was slightly diminished (S12 Table).

In sex-stratified analyses for all-cause mortality, men had slightly higher HRs than women. The strongest associations in both sexes were obtained with the “multidimensional approach” (S13 and S14 Tables).

In age-stratified analyses (>70/≤70 years), HRs for all-cause mortality were much higher in younger participants. However, the pattern of results was similar, with scores from the “multidimensional approach” showing the strongest associations with all-cause mortality in both age strata (S15 and S16 Tables).

## Discussion

Our direct comparison of the association between 35 published frailty scores and three major health outcomes in later life demonstrates that there is great variability in the strength of the prospective association with CVD, cancer, and total mortality. Moreover, the strength of the association also differed between each of the three outcomes. While most scores added predictive ability to both simple and more complex underlying models for total mortality, this was not the case for CVD or cancer.

Our finding of large heterogeneity in the magnitude of the association between different frailty scores and all-cause mortality may be due to the number and selection of variables that make up each score, along with the weight attached to each component variable in the score calculation. This is expected because these scores measure different dimensions of health, are underpinned by significantly different conceptualizations of frailty, and have different objectives of application. Therefore, the choice of a frailty score should also take into account these other aspects such as the target population (patients or general population) and the final objective of frailty assessment (clinical evaluation, research, or public health recommendations).

Interestingly, we observed that for many frailty scores, the proportional hazard assumption was not proved and the association was significantly nonuniform during follow-up time. In most of these cases, HRs for all-cause mortality were lowest directly after baseline and increased subsequently, but in some cases (40-item Frailty Index [FI40]), the opposite pattern was seen, with HRs that decreased over time. While the former set may capture information regarding underlying determinants of longer-term poor health and thus be more interesting in prognostic settings, the latter set can be hypothesized to collect information about existing health problems.

To avoid overadjustment, the most adjusted models were fitted excluding variables that were underlying variables of frailty scores. We specifically chose these models to investigate whether the score retained an association over and above a comprehensive set of clinical indicators. Our observation of heterogeneity, not only in the strength of associations but also in the degree of attenuation upon the same sets of adjustments, confirms our earlier observation that different frailty scores cannot be assumed to be interchangeable.

Our finding of a difference between analyses based on continuous scores and categorical classifications of frailty and pre-frailty indicates that the analysis with cutoffs may lead to a loss of information. This observation reflects the well-known loss of information caused by categorization of continuous variables, which assumes that the risk level is uniformly low for all below the given threshold and high for all above the threshold. Although the wish to provide users with a score with clear categories is understandable from a clinical point of view, it should be considered with caution due to the disadvantages. We have previously shown that many individuals are categorised differently by different scores [8]. Moreover, cutoff levels derived from one population may not be applicable in another.

A recent meta-analysis of 24 prospective studies, including 25 different scores, assessed the performance of frailty scores on mortality prediction and found a pooled relative risk (RR) of 1.83 (95% 1.68–1.98) for all-cause mortality based on binary/categorical frailty classifications in elderly populations (≥65 years) [7]. The result of the meta-analysis is similar to our results in the older subgroup and in our analyses based on categorical classifications. The authors found high heterogeneity OR(I^2^ statistics heterogeneity index = 95%, *p* < 0.001) and HR/RR (I^2^ statistics heterogeneity index = 98%, *p* < 0.001). They attribute this to the different populations, monitoring periods, and concepts of frailty that were included in the meta-analysis. Our study is likely to have less heterogeneous results because it is an analysis in a single data set.

We also found an association between different frailty scores and incident CVD. This was not directly expected, as frailty scores have not been designed for CVD events prediction. Our finding may be explained by the fact that component variables included in the frailty scores are also CVD events. Also, some variables are CVD symptoms and risk factors that could capture pre-existing presentations of CVD. Another explanation is that physicians are possibly less likely to treat CVD risk factors as aggressively in frail patients. In addition, frailty and CVD may share etiological pathways such as chronic low-grade inflammation [59].

There are few prospective studies of the association between frailty scores and incident CVD. Our results expand upon the evidence summarised in a review by Chen [60], which showed a significant cross-sectional association between a binary frailty classification and prevalent CVD in several previous studies [12,26,61]. White et al. reported a statistically significant association (HR: 1.8 [95% CI: 1.4–2.3]) during 30 months of follow-up in a study analysing the Phenotype of Frailty (PHF) score only [62]. Finally, Afilalo et al. demonstrated that to add frailty and disability improves the discrimination of prediction models of mortality in cardiovascular patients [63].

Frailty scores were not associated with incident cancer. As with CVD, frailty scores were not designed for the prediction of cancer. A further possible explanation is that the triggering of a cancer is a process too slow or too heterogeneous to be captured by frailty scores.

We found that almost all frailty scores improved the predictive ability of a simple age- and sex-adjusted base model for all-cause mortality. The scores that showed statistically significant added predictive value over and above the most complete base model collect information about weight loss and assess physical functioning, important prognostic determinants, and they are based on relatively few variables, which makes them easily applicable in clinical settings. However, the magnitude of the added predictive value was modest (up to 3%) and might not be clinically relevant. This could be explained in part because the basic model (age-sex) already had a good predictive ability.

Our results showed that frailty scores add predictive ability to chronological age and sex only when the outcome is mortality and are not for the prediction of incident CVD or cancer events. Ensrud et al. compared the mortality predictive ability of 2 scores, the Study of Osteoporotic Fractures (SOF) score and the PHF score, and did not find important differences in the values of the area under the curve (AUC), which were somewhat similar to those obtained by this study [64]. Also, Sourial et al. observed a modest improvement in the mortality predictive ability of age-sex models, adding models including several combinations of frailty scores [65].

Our results also show that frailty scores from the accumulation of deficit and multidimensional families have stronger associations with mortality compared with the phenotype of frailty and disability families. In their meta-analysis, Vermeiren et al. did not report differences in the magnitude of the associations using different frailty approaches [7]. Our study has the clear advantage of making a direct comparison of the predictive performance of the different scores in the same population.

### Strengths and limitations

Our study has several strengths. The large set of scores included allows for the comparison between families of scores as well as between individual scores.

We performed state-of-the-art multiple imputation to deal with missing data, thereby making optimal use of the available events and follow-up time. We decided to impute underlying variables into their more basic form, which means that we imputed binary, categorical, and continuous variables with different models. Continuous variables were not categorised. The goal was to obtain the most plausible values of frailty scores without losing information. We are convinced that frailty scores with underlying imputed variables give less biased results and increase statistical power and accuracy. With frailty scores that have missing values for some underlying variables, it is likely that a lot of information will be lost. In addition, when some variables have missing data, we cannot rule out a missing at random mechanism. For example, a missing physical examination may be observed more frequently in a frail participant, because he could reject the test for fear of falling. There is strong evidence of the need to impute missing data, especially when the missing mechanism is not totally at random [66].

In addition, our results fill a gap especially concerning the scarce information about the relationship between frailty scores and incident CVD and cancer. The results of this study are directly applicable to the general elderly English population and are probably also generalizable to similar populations in other European countries.

A limitation of our analysis was that we had to tailor some variables to calculate certain frailty scores. We based this adaptation on published studies when possible. Another important limitation was the different follow-up duration for total mortality compared to CVD and cancer. Almost 100% of ELSA participants were followed for all-cause mortality based on reliable and objective mortality registries. In contrast, more participants were lost to follow-up with regard to CVD and cancer end points. This could be a source of bias if loss to follow-up was associated both with frailty and with the two outcomes, because participants who were lost to follow-up could be precisely those who experienced a cardiovascular or cancer event. Also, the ascertainment of CVD and cancer was based on self-reports, possibly leading to misclassification due to differential recall. However, in both cases, the most likely impact of these sources of selection would be an underestimation of a true effect rather than identification of a spurious association. Finally, while the ELSA study is a rich source of data and well suited to the study of frailty, we performed a secondary data analysis, which meant that we had to adapt our data analysis to the existing data.

The best performing scores for all-cause mortality using the continuous analysis were multidimensional and accumulation of deficit approach. The multidimensional scores can have few variables, and in consequence, they are easy to apply in a clinical setting. These scores are tailored to capture features related to ill-health in later life over and above the obvious things we can obtain from a simple clinical history, such as polymedication, weight loss, depression symptoms, cognition, and self-reported health. Based on our data, we think that the isolated presence of comorbidity and/or polypharmacy is not enough to evaluate the presence of frailty, which means it is also necessary to measure physical and/or cognitive function.

### Conclusions

It seems that while some scores can be regarded as a simple summary indicator for known risk factors, other scores capture other important information, such as self-reported health, medications, cognition, and disability. In our analysis of frailty categories, the best performing scores included physical functioning assessment. Overall, we found that multidimensional frailty scores have the strongest association and largest additional predictive performance for mortality outcomes.

Frailty scores could have been considered clinically useful tools for identifying patients at higher risk of imminent death. However, the observed additional predictive ability for all-cause mortality is low, which reduces their clinical value for separating individuals who will experience from those who will not experience the outcome.

There are marked differences between scores with regard to their complexity as well as strength and stability of association, with all-cause mortality probably due to a great heterogeneity in the conception of different scores. This means that users of frailty scores should carefully balance the feasibility of measurement with a score’s performance. Our results provide evidence to guide clinicians, researchers, and public health practitioners in striking this balance.

We think that future research should focus on the study of the trajectories of frailty scores. Frailty should be assessed with the most adapted instrument for this purpose. This approach could help identify individuals or characteristics of frailty early in time to establish useful interventions in patients and/or the general population.

## Supporting information

S1 Text STROBE checklist. (DOCX)

S1 Table Adjustment covariates for model 3. (DOCX)

S2 Table Mortality hazard ratios of frailty scores assessed in intervals from 1 to 7 years: Age-adjusted model and continuous analysis. (DOCX)

S3 Table Mortality hazard ratios of frailty scores assessed in intervals from 1 to 7 years: Age-adjusted model and categorical analysis. (DOCX)

S4 Table Cardiovascular events hazard ratios of frailty scores (*n* = 4,554) calculated at median time follow-up (2.5 years). (DOCX)

S5 Table Cardiovascular hazard ratios of frailty scores assessed in intervals from 1 to 7 years: Age-adjusted model and continuous analysis. (DOCX)

S6 Table Cardiovascular hazard ratios of frailty scores assessed in intervals from 1 to 7 years: Age-adjusted model and categorical analysis. (DOCX)

S7 Table Cancer hazard ratios of frailty scores (*n* = 4,792) calculated at median time follow-up (2.5 years). (DOCX)

S8 Table Cancer hazard ratios of frailty scores assessed in intervals from 1 to 7 years: Age-adjusted model and continuous analysis. (DOCX)

S9 Table Cancer hazard ratios of frailty scores assessed in intervals from 1 to 7 years: Age-adjusted model and categorical analysis. (DOCX)

S10 Table Discriminative assessment of cardiovascular models using Harrell's C statistic (*n* = 4,554). (DOCX)

S11 Table Discriminative assessment of cancer models using Harrell's C statistic (*n* = 4,792). (DOCX)

S12 Table Sensitivity analysis: Mortality hazard ratios of frailty scores (*n* = 5,253). (DOCX)

S13 Table Mortality hazard ratios of frailty scores in men (*n* = 2,377) calculated at median time follow-up (3.5 years). (DOCX)

S14 Table Mortality hazard ratios of frailty scores in women (*n* = 2,917) calculated at median time follow-up (3.5 years). (DOCX)

S15 Table Mortality hazard ratios of frailty scores in participants older than 70 years (*n* = 2,536) calculated at median time follow-up (3.5 years). (DOCX)

S16 Table Mortality hazard ratios of frailty scores in participants of 70 years and younger (*n* = 2,758) calculated at median time follow-up (3.5 years). (DOCX)

## Abbreviations

AUC: area under the curve
BDE: Beaver Dam Eye Study Index
BFI: Brief Frailty Index
BMI: body mass index
CASP-19: 19-item scale control, autonomy, pleasure, and self-realization
CGA: Comprehensive Geriatric Assessment
CGAST: Comprehensive Geriatric Assessment Screening Tests
COPD: chronic obstructive pulmonary disease
CSBA: Conselice Study of Brain Aging Score
CVD: cardiovascular disease
EFIP: Evaluative Frailty Index for Physical Activity
EFS: Edmonton Frail Scale
ELSA: English Longitudinal Study of Ageing
FI40: 40-item Frailty Index
FI70: 70-item Frailty Index (SHARE)
FIBLSA: Frailty Index Beijing Longitudinal Study of Ageing
FiND: Frail Non-Disabled Questionnaire
FS: Frail Scale
FSS: Frailty Staging System
G8: G-8 Geriatric Screening Tool
GFI: Groningen Frailty Indicator
HR: hazard ratio
HRCA: Hebrew Rehabilitation Center for Aged Vulnerability Index
HSF: Health Status Form
IFQ: Inter-Frail Questionnaire
MFS: Modified Frailty Score
MPHF: Modified Phenotype of Frailty
NLTCS: Long Term Care Survey Frailty Index
OR: Odds ratio
PFI: Physical Frailty Index
PHF: Phenotype of Frailty
RR: relative risk
SDFI: Static/Dynamic Frailty Index
SHARE: Survey of Health, Ageing and Retirement in Europe
SHCFS: Canadian Study of Health and Aging Clinical Frailty Scale
SI: Screening Instrument
SOF: Study of Osteoporotic Fractures
SPPB: Short Physical Performance Battery
SPQ: Sherbrooke Postal Questionnaire
TFI: Tilburg Frailty Indicator
VES13: Vulnerable Elders Survey
WHOAFC: World Health Organization Assessment of Functional Capacity
WHRH: WHOAFC and self-reported health
ZED1: ZutPhen Elderly Study (Physical Activity and Low Energy)
ZED2: ZutPhen Elderly Study (Physical Activity and Weight Loss)
ZED3: ZutPhen Elderly Study (Physical Activity and Low BMI)
