# Supplementary material for: Comparative analysis of the association between 35 frailty scores and cardiovascular events, cancer, and total mortality in an elderly general population in England: An observational study
Source: PLoS Med. 2018 Mar 27;15(3):e1002543. doi: 10.1371/journal.pmed.1002543 (PMC5870943; doi:10.1371/journal.pmed.1002543)
Supplement: S1 Table — (DOCX) [file pmed.1002543.s002.docx]

| **S1 Table.** Adjustment covariates for model 3^1^   \| Score \| sex \| Smoking status \| Alcohol \| Physical activity \| BMI \| Diabetes \| HTA \| CVD \| cancer \| anaemia \| COPD \| Arthritis \| Neuropsy \| Depression \| Cognition \| self-rated health \| quality of life \| \| --- \| --- \| --- \| --- \| --- \| --- \| --- \| --- \| --- \| --- \| --- \| --- \| --- \| --- \| --- \| --- \| --- \| --- \| \| BDE \| 1 \| 1 \| 1 \| 0 \| 1 \| 1 \| 1 \| 1 \| 1 \| 1 \| 0 \| 1 \| 1 \| 1 \| 1 \| 1 \| 1 \| \| BFI \| 1 \| 1 \| 1 \| 1 \| 0 \| 1 \| 1 \| 1 \| 1 \| 1 \| 1 \| 1 \| 1 \| 0 \| 0 \| 1 \| 1 \| \| CGA \| 1 \| 1 \| 1 \| 0 \| 0 \| 0 \| 0 \| 0 \| 0 \| 1 \| 0 \| 0 \| 1 \| 0 \| 0 \| 0 \| 1 \| \| CGAST \| 1 \| 1 \| 1 \| 1 \| 0 \| 0 \| 0 \| 1 \| 1 \| 1 \| 0 \| 1 \| 1 \| 0 \| 0 \| 1 \| 1 \| \| CSBA \| 1 \| 1 \| 1 \| 1 \| 0 \| 0 \| 0 \| 1 \| 1 \| 1 \| 0 \| 1 \| 1 \| 0 \| 0 \| 1 \| 1 \| \| EFIP \| 1 \| 1 \| 1 \| 0 \| 1 \| 1 \| 0 \| 0 \| 1 \| 1 \| 0 \| 0 \| 1 \| 0 \| 0 \| 0 \| 1 \| \| EFS \| 1 \| 1 \| 1 \| 0 \| 0 \| 0 \| 0 \| 0 \| 1 \| 1 \| 0 \| 0 \| 1 \| 0 \| 0 \| 0 \| 1 \| \| FI40 \| 1 \| 1 \| 1 \| 0 \| 0 \| 0 \| 0 \| 0 \| 0 \| 1 \| 0 \| 0 \| 1 \| 0 \| 0 \| 0 \| 1 \| \| FI70 \| 1 \| 1 \| 1 \| 0 \| 1 \| 0 \| 0 \| 0 \| 0 \| 1 \| 0 \| 0 \| 0 \| 0 \| 0 \| 0 \| 1 \| \| FIBLSA \| 1 \| 1 \| 1 \| 0 \| 1 \| 1 \| 0 \| 0 \| 1 \| 1 \| 1 \| 0 \| 1 \| 0 \| 0 \| 1 \| 1 \| \| FiND \| 1 \| 1 \| 1 \| 0 \| 0 \| 1 \| 1 \| 1 \| 1 \| 1 \| 1 \| 1 \| 1 \| 1 \| 1 \| 1 \| 1 \| \| FS \| 1 \| 1 \| 1 \| 0 \| 0 \| 0 \| 0 \| 0 \| 1 \| 1 \| 0 \| 0 \| 0 \| 0 \| 1 \| 1 \| 1 \| \| FSS \| 1 \| 1 \| 1 \| 0 \| 1 \| 1 \| 1 \| 1 \| 1 \| 1 \| 1 \| 1 \| 1 \| 1 \| 0 \| 1 \| 1 \| \| G8 \| 1 \| 1 \| 1 \| 1 \| 0 \| 0 \| 0 \| 0 \| 1 \| 1 \| 1 \| 1 \| 0 \| 1 \| 1 \| 0 \| 1 \| \| GFI \| 1 \| 1 \| 1 \| 0 \| 0 \| 0 \| 0 \| 0 \| 1 \| 1 \| 1 \| 1 \| 1 \| 0 \| 0 \| 1 \| 1 \| \| HRCA \| 1 \| 1 \| 1 \| 0 \| 1 \| 1 \| 1 \| 1 \| 1 \| 1 \| 1 \| 1 \| 1 \| 1 \| 1 \| 1 \| 1 \| \| HSF \| 1 \| 1 \| 1 \| 1 \| 1 \| 0 \| 0 \| 0 \| 1 \| 1 \| 1 \| 1 \| 1 \| 1 \| 1 \| 1 \| 1 \| \| IFQ \| 1 \| 1 \| 1 \| 0 \| 0 \| 0 \| 0 \| 0 \| 1 \| 1 \| 1 \| 1 \| 1 \| 1 \| 0 \| 1 \| 1 \| \| MFS \| 1 \| 1 \| 1 \| 0 \| 0 \| 1 \| 1 \| 1 \| 1 \| 1 \| 0 \| 1 \| 1 \| 1 \| 0 \| 1 \| 1 \| \| MPHF \| 1 \| 1 \| 1 \| 0 \| 0 \| 1 \| 1 \| 1 \| 1 \| 1 \| 1 \| 1 \| 1 \| 0 \| 0 \| 1 \| 1 \| \| NLTCS \| 1 \| 1 \| 1 \| 0 \| 1 \| 0 \| 0 \| 0 \| 1 \| 1 \| 1 \| 0 \| 0 \| 1 \| 1 \| 0 \| 1 \| \| PFI \| 1 \| 1 \| 1 \| 0 \| 1 \| 1 \| 1 \| 1 \| 1 \| 1 \| 1 \| 1 \| 1 \| 1 \| 1 \| 1 \| 1 \| \| PHF \| 1 \| 1 \| 1 \| 0 \| 0 \| 1 \| 1 \| 1 \| 1 \| 1 \| 1 \| 1 \| 1 \| 1 \| 1 \| 1 \| 1 \| \| SDFI \| 1 \| 1 \| 1 \| 1 \| 0 \| 1 \| 1 \| 1 \| 1 \| 1 \| 0 \| 1 \| 1 \| 0 \| 0 \| 1 \| 0 \| \| SHCFS \| 1 \| 1 \| 1 \| 0 \| 1 \| 1 \| 1 \| 1 \| 1 \| 1 \| 1 \| 1 \| 1 \| 0 \| 1 \| 0 \| 1 \| \| SI \| 1 \| 1 \| 1 \| 1 \| 1 \| 1 \| 1 \| 1 \| 1 \| 1 \| 1 \| 1 \| 1 \| 0 \| 1 \| 1 \| 1 \| \| SOF \| 1 \| 1 \| 1 \| 0 \| 0 \| 1 \| 1 \| 1 \| 1 \| 1 \| 1 \| 1 \| 1 \| 1 \| 1 \| 1 \| 1 \| \| SPPB \| 1 \| 1 \| 1 \| 0 \| 1 \| 1 \| 1 \| 1 \| 1 \| 1 \| 1 \| 1 \| 1 \| 1 \| 1 \| 1 \| 1 \| \| SPQ \| 1 \| 1 \| 1 \| 0 \| 1 \| 0 \| 0 \| 0 \| 1 \| 1 \| 1 \| 1 \| 1 \| 1 \| 0 \| 1 \| 1 \| \| TFI \| 1 \| 1 \| 1 \| 0 \| 0 \| 1 \| 1 \| 1 \| 1 \| 1 \| 1 \| 1 \| 1 \| 0 \| 0 \| 0 \| 1 \| \| VES13 \| 1 \| 1 \| 1 \| 0 \| 1 \| 1 \| 1 \| 1 \| 1 \| 1 \| 1 \| 1 \| 1 \| 1 \| 1 \| 0 \| 1 \| \| WHRH \| 1 \| 1 \| 1 \| 0 \| 1 \| 1 \| 1 \| 1 \| 1 \| 1 \| 1 \| 1 \| 1 \| 1 \| 1 \| 0 \| 1 \| \| ZED1 \| 1 \| 1 \| 1 \| 0 \| 1 \| 1 \| 1 \| 1 \| 1 \| 1 \| 1 \| 1 \| 1 \| 1 \| 1 \| 1 \| 1 \| \| ZED2 \| 1 \| 1 \| 1 \| 0 \| 0 \| 1 \| 1 \| 1 \| 1 \| 1 \| 1 \| 1 \| 1 \| 1 \| 1 \| 1 \| 1 \| \| ZED3 \| 1 \| 1 \| 1 \| 0 \| 0 \| 1 \| 1 \| 1 \| 1 \| 1 \| 1 \| 1 \| 1 \| 1 \| 1 \| 1 \| 1 \| |
| --- | --- | --- | --- | --- | --- | --- | --- | --- | --- | --- | --- | --- | --- | --- | --- | --- | --- | --- | --- | --- | --- | --- | --- | --- | --- | --- | --- | --- | --- | --- | --- | --- | --- | --- | --- | --- | --- | --- | --- | --- | --- | --- | --- | --- | --- | --- | --- | --- | --- | --- | --- | --- | --- | --- | --- | --- | --- | --- | --- | --- | --- | --- | --- | --- | --- | --- | --- | --- | --- | --- | --- | --- | --- | --- | --- | --- | --- | --- | --- | --- | --- | --- | --- | --- | --- | --- | --- | --- | --- | --- | --- | --- | --- | --- | --- | --- | --- | --- | --- | --- | --- | --- | --- | --- | --- | --- | --- | --- | --- | --- | --- | --- | --- | --- | --- | --- | --- | --- | --- | --- | --- | --- | --- | --- | --- | --- | --- | --- | --- | --- | --- | --- | --- | --- | --- | --- | --- | --- | --- | --- | --- | --- | --- | --- | --- | --- | --- | --- | --- | --- | --- | --- | --- | --- | --- | --- | --- | --- | --- | --- | --- | --- | --- | --- | --- | --- | --- | --- | --- | --- | --- | --- | --- | --- | --- | --- | --- | --- | --- | --- | --- | --- | --- | --- | --- | --- | --- | --- | --- | --- | --- | --- | --- | --- | --- | --- | --- | --- | --- | --- | --- | --- | --- | --- | --- | --- | --- | --- | --- | --- | --- | --- | --- | --- | --- | --- | --- | --- | --- | --- | --- | --- | --- | --- | --- | --- | --- | --- | --- | --- | --- | --- | --- | --- | --- | --- | --- | --- | --- | --- | --- | --- | --- | --- | --- | --- | --- | --- | --- | --- | --- | --- | --- | --- | --- | --- | --- | --- | --- | --- | --- | --- | --- | --- | --- | --- | --- | --- | --- | --- | --- | --- | --- | --- | --- | --- | --- | --- | --- | --- | --- | --- | --- | --- | --- | --- | --- | --- | --- | --- | --- | --- | --- | --- | --- | --- | --- | --- | --- | --- | --- | --- | --- | --- | --- | --- | --- | --- | --- | --- | --- | --- | --- | --- | --- | --- | --- | --- | --- | --- | --- | --- | --- | --- | --- | --- | --- | --- | --- | --- | --- | --- | --- | --- | --- | --- | --- | --- | --- | --- | --- | --- | --- | --- | --- | --- | --- | --- | --- | --- | --- | --- | --- | --- | --- | --- | --- | --- | --- | --- | --- | --- | --- | --- | --- | --- | --- | --- | --- | --- | --- | --- | --- | --- | --- | --- | --- | --- | --- | --- | --- | --- | --- | --- | --- | --- | --- | --- | --- | --- | --- | --- | --- | --- | --- | --- | --- | --- | --- | --- | --- | --- | --- | --- | --- | --- | --- | --- | --- | --- | --- | --- | --- | --- | --- | --- | --- | --- | --- | --- | --- | --- | --- | --- | --- | --- | --- | --- | --- | --- | --- | --- | --- | --- | --- | --- | --- | --- | --- | --- | --- | --- | --- | --- | --- | --- | --- | --- | --- | --- | --- | --- | --- | --- | --- | --- | --- | --- | --- | --- | --- | --- | --- | --- | --- | --- | --- | --- | --- | --- | --- | --- | --- | --- | --- | --- | --- | --- | --- | --- | --- | --- | --- | --- | --- | --- | --- | --- | --- | --- | --- | --- | --- | --- | --- | --- | --- | --- | --- | --- | --- | --- | --- | --- | --- | --- | --- | --- | --- | --- | --- | --- | --- | --- | --- | --- | --- | --- | --- | --- | --- | --- | --- | --- | --- | --- | --- | --- | --- | --- | --- | --- | --- | --- | --- | --- | --- | --- | --- | --- | --- | --- | --- | --- | --- | --- | --- | --- | --- | --- | --- | --- | --- | --- | --- | --- | --- | --- | --- | --- | --- | --- | --- | --- | --- | --- | --- | --- | --- | --- | --- | --- | --- | --- | --- | --- | --- | --- | --- | --- | --- | --- | --- | --- | --- | --- | --- | --- | --- | --- | --- | --- | --- | --- | --- | --- | --- | --- | --- | --- | --- | --- | --- | --- | --- | --- | --- | --- | --- | --- | --- | --- | --- | --- | --- | --- | --- | --- | --- | --- | --- | --- | --- | --- | --- | --- | --- | --- | --- | --- | --- | --- | --- | --- | --- | --- | --- | --- | --- | --- | --- | --- | --- | --- | --- | --- | --- | --- |

^1^Included in model 3=1/excluded in model 3=0.

Abbreviations: Alcohol=alcohol consumption; HTA= hypertension; CVD= cardiovascular disease; COPD=chronic obstructive pulmonary disease; Neuropsy=neuropsychiatric problems.

Abbreviations frailty scores: BDE= Beaver Dam Eye Study Index. BFI= Brief Frailty Index. CGA= Comprehensive Geriatric Assessment. CGAST= Comprehensive Geriatric Assessment Screening Tests. CSBA= Conselice Study of Brain Aging Score. EFIP= Evaluative Frailty Index for Physical Activity. EFS= Edmonton Frail Scale. FI40= 40-item Frailty Index. FI70= 70-item Frailty Index (SHARE). FIBLSA= Frailty Index Beijing Longitudinal Study of Ageing. FiND= Frail Non-Disabled Questionnaire. FS= Frail Scale. FSS= Frailty Staging System. G8= G-8 Geriatric Screening Tool. GFI= Groningen Frailty Indicator. HRCA= Hebrew Rehabilitation Center for Aged Vulnerability Index. HSF= Health Status Form. IFQ= Inter-Frail Questionnaire. MFS= Modified Frailty Score. MPHF= Modified Phenotype of Frailty. NLTCS= Long Term Care Survey Frailty Index. PFI= Physical Frailty Index. PHF= Phenotype of Frailty. SDFI=, Static/Dynamic Frailty Index. SHCFS= Canadian Study of Health and Aging Clinical Frailty Scale·. SI= Screening Instrument. SOF= Study of Osteoporotic Fractures. SPPB= Short Physical Performance Battery. SPQ= Sherbrooke Postal Questionnaire. TFI= Tilburg Frailty Indicator. VES13= Vulnerable Elders Survey. WHRH= WHOAFC & self-reported health. ZED1= ZutPhen Elderly Study (Physical Activity & Low Energy). ZED2= ZutPhen Elderly Study (Physical Activity & Weight Loss). ZED3= ZutPhen Elderly Study (Physical Activity & Low BMI).
