# Supplementary material for: Comparative analysis of the association between 35 frailty scores and cardiovascular events, cancer, and total mortality in an elderly general population in England: An observational study
Source: PLoS Med. 2018 Mar 27;15(3):e1002543. doi: 10.1371/journal.pmed.1002543 (PMC5870943; doi:10.1371/journal.pmed.1002543)
Supplement: S2 Table — (DOCX) [file pmed.1002543.s003.docx]

**S2 Table.** Mortality hazard ratios of frailty scores assessed in intervals from1 to 7 years^1^: age-adjusted model and continuous analysis

| Scores | HR 1 (LCI; UCI) | HR 2 (LCI; UCI) | HR 3 (LCI; UCI) | HR 3.5 (LCI; UCI) | HR 4 (LCI; UCI) | HR 5 (LCI; UCI) | HR 6 (LCI; UCI) | HR 7 (LCI; UCI) |
| --- | --- | --- | --- | --- | --- | --- | --- | --- |
| BDE | 1.6 (1.2; 2.2) | 2.9 (2.1; 4.0) | 4.0 (2.9; 5.6) | 4.6 (3.3; 6.4) | 5.2 (3.7; 7.1) | 6.2 (4.5; 8.6) | 7.3 (5.2; 10.1) | 8.3 (6.0; 11.5) |
| BFI | 1.0 (0.7; 1.5) | 2.0 (1.4; 3.0) | 3.1 (2.1; 4.6) | 3.6 (2.4; 5.4) | 4.1 (2.8; 6.2) | 5.2 (3.5; 7.8) | 6.3 (4.1; 9.4) | 7.3 (4.9; 11.0) |
| CGA | 1.9 (1.0; 3.6) | 6.0 (3.1; 11.8) | 12.0 (6.1; 23.5) | 15.6 (8.0; 30.5) | 19.5 (10.0; 38.2) | 28.5 (14.6; 55.8) | 38.9 (19.5; 76.1) | 50.5 (25.8; 98.8) |
| CGAST | 2.4 (1.4; 3.9) | 5.4 (3.3; 9.0) | 8.8 (5.3; 14.6) | 10.6 (6.4; 17.6) | 12.4 (7.5; 20.6) | 16.2 (9.7; 26.9) | 20.1 (12.4; 33.5) | 24.2 (14.6; 40.2) |
| CSBA | 5.8 (3.4; 9.9) | 13.4 (7.9; 22.8) | 21.8 (12.8; 37.0) | 26.2 (15.4; 44.5) | 30.7 (18.1; 52.2) | 40.1 (23.6; 68.2) | 49.9 (30.7; 84.8) | 60.0 (35.3; 102.0) |
| EFIP | 1.8 (1.1; 3.1) | 4.7 (2.7; 8.1) | 8.2 (4.8; 14.0) | 10.1 (5.9; 17.3) | 12.1 (7.1; 20.8) | 16.5 (9.6; 28.2) | 21.1 (12.1; 36.2) | 26.1 (15.2; 44.7) |
| EFS | 3.3 (1.9; 5.8) | 8.3 (4.7; 14.4) | 14.2 (8.1; 24.7) | 17.4 (10.0; 30.3) | 20.7 (11.9; 36.1) | 27.8 (16.0; 48.5) | 35.4 (20.7; 61.8) | 43.5 (24.9; 75.8) |
| FI40 | 22.7 (15.4; 33.5) | 19.7 (13.3; 29.0) | 18.1 (12.3; 26.6) | 17.5 (11.9; 25.8) | 17.0 (11.5; 25.1) | 16.2 (11.0; 23.9) | 15.6 (17.0; 23.0) | 15.1 (10.3; 22.3) |
| FI70 | 2.4 (1.4; 4.1) | 6.1 (3.6; 10.5) | 10.6 (6.2; 18.1) | 13.0 (7.6; 22.4) | 15.6 (9.1; 26.8) | 21.1 (12.3; 36.2) | 27.1 (15.6; 46.4) | 33.3 (19.4; 57.2) |
| FIBLSA | 1.1 (0.6; 2.0) | 3.2 (1.8; 5.9) | 6.2 (3.4; 11.3) | 7.9 (4.3; 14.4) | 9.7 (5.3; 17.8) | 13.9 (7.6; 25.4) | 18.5 (9.7; 33.8) | 23.6 (12.9; 43.2) |
| FiND | 1.7 (1.2; 2.4) | 2.9 (2.1; 4.1) | 4.0 (2.9; 5.6) | 4.6 (3.2; 6.4) | 5.1 (3.6; 7.1) | 6.0 (4.3; 8.5) | 7.0 (5.1; 9.8) | 7.9 (5.6; 11.1) |
| FS | 1.6 (1.1; 2.4) | 3.0 (2.0; 4.5) | 4.3 (2.9; 6.4) | 4.9 (3.3; 7.3) | 5.5 (3.7; 8.2) | 6.6 (4.5; 9.9) | 7.8 (5.5; 11.6) | 8.9 (6.0; 13.3) |
| FSS | 1.0 (0.7; 1.4) | 1.9 (1.3; 2.8) | 2.9 (2.0; 4.2) | 3.3 (2.3; 4.9) | 3.8 (2.6; 5.6) | 4.7 (3.3; 6.9) | 5.7 (3.8; 8.2) | 6.6 (4.5; 9.6) |
| G8 | 3.3 (2.0; 5.5) | 8.5 (5.1; 14.2) | 14.7 (8.8; 24.6) | 18.2 (10.8; 30.4) | 21.8 (13.0; 36.5) | 29.5 (17.6; 49.4) | 37.8 (21.8; 63.4) | 46.7 (27.9; 78.2) |
| GFI | 1.9 (1.2; 3.1) | 4.4 (2.7; 7.1) | 7.1 (4.3; 11.5) | 8.5 (5.2; 13.9) | 10.0 (6.1; 16.2) | 13.0 (8.0; 21.2) | 16.1 (10.0; 26.3) | 19.4 (11.9; 31.5) |
| HRCA | 1.1 (0.7; 1.8) | 2.6 (1.6; 4.2) | 4.2 (2.6; 6.8) | 5.0 (3.1; 8.1) | 5.9 (3.7; 9.5) | 7.7 (4.8; 12.4) | 9.6 (5.9; 15.5) | 11.5 (7.2; 18.6) |
| HSF | 1.3 (0.8; 1.9) | 2.9 (1.9; 4.4) | 4.6 (3.0; 7.1) | 5.5 (3.6; 8.5) | 6.5 (4.3; 9.9) | 8.5 (5.5; 12.9) | 10.5 (6.5; 16.0) | 12.6 (8.3; 19.3) |
| IFQ | 1.6 (1.0; 2.6) | 3.8 (2.3; 6.2) | 6.3 (3.9; 10.3) | 7.7 (4.7; 12.5) | 9.0 (5.5; 14.8) | 11.9 (7.3; 19.5) | 15.0 (9.0; 24.4) | 18.1 (11.1; 29.6) |
| MFS | 2.3 (1.6; 3.3) | 4.3 (3.0; 6.3) | 6.3 (4.3; 9.2) | 7.3 (5.0; 10.7) | 8.3 (5.7; 12.1) | 10.2 (7.0; 14.9) | 12.1 (8.3; 17.6) | 14.0 (9.6; 20.4) |
| MPHF | 2.0 (1.4; 2.8) | 3.7 (2.7; 5.3) | 5.4 (3.8; 7.6) | 6.2 (4.4; 8.7) | 7.0 (5.0; 9.8) | 8.5 (6.0; 11.9) | 10.0 (7.0; 14.1) | 11.5 (8.2; 16.1) |
| NLTCS | 1.0 (0.5; 2.0) | 3.6 (1.7; 7.6) | 7.8 (3.7; 16.5) | 10.4 (4.9; 22.1) | 13.4 (6.3; 28.5) | 20.6 (9.7; 43.6) | 29.1 (13.4; 61.7) | 39.0 (18.4; 82.7) |
| PFI | 1.1 (0.8; 1.5) | 1.7 (1.2; 2.3) | 2.2 (1.6; 3.0) | 2.4 (1.7; 3.3) | 2.6 (1.9; 3.6) | 3.0 (2.2; 4.2) | 3.4 (2.6; 4.7) | 3.8 (2.7; 5.2) |
| PHF | 1.9 (1.4; 2.7) | 3.5 (2.5; 4.9) | 5.0 (3.6; 6.9) | 5.7 (4.1; 7.9) | 6.4 (4.6; 8.9) | 7.7 (5.5; 10.8) | 9.0 (6.4; 12.6) | 10.3 (7.4; 14.4) |
| SDFI | 2.1 (1.3; 3.4) | 4.5 (2.8; 7.2) | 7.0 (4.4; 11.1) | 8.3 (5.2; 13.2) | 9.6 (6.0; 15.2) | 12.2 (7.7; 19.4) | 14.9 (9.6; 23.7) | 17.6 (11.1; 28.0) |
| SHCFS | 1.3 (1.0; 1.9) | 2.3 (1.6; 3.2) | 3.2 (2.3; 4.5) | 3.6 (2.6; 5.0) | 4.0 (2.8; 5.6) | 4.7 (3.4; 6.7) | 5.5 (4.0; 7.7) | 6.2 (4.4; 8.7) |
| SI | 0.6 (0.4; 1.1) | 1.6 (0.9; 2.7) | 2.8 (1.6; 4.7) | 3.4 (2.0; 5.8) | 4.1 (2.4; 6.9) | 5.5 (3.2; 9.4) | 7.0 (4.1; 12.0) | 8.6 (5.0; 14.7) |
| SOF | 1.6 (1.1; 2.2) | 2.6 (1.8; 3.7) | 3.5 (2.4; 5.0) | 3.9 (2.7; 5.6) | 4.3 (3.0; 6.2) | 5.1 (3.5; 7.3) | 5.8 (4.3; 8.3) | 6.5 (4.5; 9.3) |
| SPPB | 1.7 (1.2; 2.6) | 3.5 (2.3; 5.2) | 5.2 (3.5; 7.8) | 6.1 (4.1; 9.1) | 7.0 (4.7; 10.4) | 8.7 (5.8; 13.0) | 10.4 (7.0; 15.6) | 12.2 (8.2; 18.1) |
| SPQ | 0.6 (0.3; 0.9) | 1.4 (0.8; 2.3) | 2.3 (1.4; 3.9) | 2.9 (1.7; 4.7) | 3.4 (2.1; 5.6) | 4.5 (2.7; 7.5) | 5.7 (3.4; 9.4) | 7.0 (4.2; 11.5) |
| TFI | 2.6 (1.7; 4.0) | 5.3 (3.5; 8.2) | 8.1 (5.3; 12.4) | 9.5 (6.2; 14.6) | 10.9 (7.1; 16.8) | 13.8 (9.0; 21.1) | 16.6 (10.9; 25.5) | 19.5 (12.7; 30.0) |
| VES13 | 1.4 (0.9; 2.1) | 3.1 (2.0; 4.7) | 4.9 (3.2; 7.4) | 5.8 (3.8; 8.8) | 6.8 (4.5; 10.3) | 8.7 (5.7; 13.2) | 10.7 (6.8; 16.3) | 12.8 (8.4; 19.4) |
| WHRH | 1.3 (0.9; 2.0) | 2.5 (1.7; 3.8) | 3.7 (2.4; 5.5) | 4.2 (2.8; 6.4) | 4.8 (3.2; 7.2) | 5.9 (3.9; 8.9) | 7.0 (4.8; 10.6) | 8.1 (5.4; 12.2) |
| ZED1 | 1.2 (0.9; 1.6) | 1.9 (1.4; 2.6) | 2.6 (1.9; 3.5) | 2.9 (2.1; 3.9) | 3.2 (2.4; 4.3) | 3.7 (2.8; 5.1) | 4.3 (3.2; 5.8) | 4.8 (3.6; 6.5) |
| ZED2 | 1.7 (1.2; 2.3) | 2.6 (1.9; 3.5) | 3.3 (2.5; 4.5) | 3.7 (2.7; 4.9) | 4.0 (3.0; 5.4) | 4.6 (3.4; 6.2) | 5.2 (4.0; 6.9) | 5.7 (4.2; 7.6) |
| ZED3 | 1.0 (0.7; 1.5) | 1.9 (1.3; 2.9) | 2.8 (1.9; 4.1) | 3.2 (2.2; 4.7) | 3.6 (2.4; 5.3) | 4.4 (3.0; 6.4) | 5.1 (3.6; 7.5) | 5.9 (4.0; 8.7) |

^1^Hazard ratios calculated from age at baseline to age at the end of the interval.

BDE= Beaver Dam Eye Study Index. BFI= Brief Frailty Index. CGA= Comprehensive Geriatric Assessment. CGAST= Comprehensive Geriatric Assessment Screening Tests. CSBA= Conselice Study of Brain Aging Score. EFIP= Evaluative Frailty Index for Physical Activity. EFS= Edmonton Frail Scale. FI40= 40-item Frailty Index. FI70= 70-item Frailty Index. FIBLSA= Frailty Index Beijing Longitudinal Study of Ageing. FIND= Frail Non-Disabled Questionnaire. FS= Frail Scale. FSS= Frailty Staging System. G8= G-8 Geriatric Screening Tool. GFI= Groningen Frailty Indicator. HRCA= Hebrew Rehabilitation Center for Aged Vulnerability Index. HSF= Health Status Form. IFQ= Inter-Frail Questionnaire. MFS= Modified Frailty Score. MPHF= Modified Phenotype of Frailty. NLTCS= Long Term Care Survey Frailty Index. PFI= Physical Frailty Index. PHF= Phenotype of Frailty. SDF=, Static/Dynamic Frailty Index. SHCFS= Canadian Study of Health and Aging Clinical Frailty Scale·. SI= Screening Instrument. SOF= Study of Osteoporotic Fractures. SPPB= Short Physical Performance Battery. SPQ= Sherbrooke Postal Questionnaire. TFI= Tilburg Frailty Indicator. VES13= Vulnerable Elders Survey. WHRH= WHOAFC & self-reported health. ZED1= ZutPhen Elderly Study (Physical Activity & Low Energy). ZED2= ZutPhen Elderly Study (Physical Activity & Weight Loss). ZED3= ZutPhen Elderly Study (Physical Activity & Low BMI).
