# Supplementary material for: Comparative analysis of the association between 35 frailty scores and cardiovascular events, cancer, and total mortality in an elderly general population in England: An observational study
Source: PLoS Med. 2018 Mar 27;15(3):e1002543. doi: 10.1371/journal.pmed.1002543 (PMC5870943; doi:10.1371/journal.pmed.1002543)
Supplement: S3 Table — (DOCX) [file pmed.1002543.s004.docx]

**S3 Table.** Mortality hazard ratios of frailty scores assessed in intervals from1 to 7 years^1^: age-adjusted model and categorical analysis

| Scores | HR1 (LCI; UCI) | HR2 (LCI; UCI) | HR3 (LCI; UCI) | HR3.5 (LCI; UCI) | HR4 (LCI; UCI) | HR5 (LCI; UCI) | HR6 (LCI; UCI) | HR7 (LCI; UCI) |
| --- | --- | --- | --- | --- | --- | --- | --- | --- |
| BFI frail | 0.7 (0.5; 0.9) | 1.1 (0.6; 1.8) | 1.4 (0.7; 2.5) | 1.5 (0.8; 2.9) | 1.6 (1.8; 3.3) | 1.8 (0.9; 4.0) | 2.1 (0.9; 4.7) | 2.3 (0.9; 5.4) |
| CGA frail | 1.1 (0.9; 1.5) | 1.8 (1.2; 2.7) | 2.3 (1.4; 3.9) | 2.6 (1.4; 4.5) | 2.8 (2.7; 5.1) | 3.2 (1.4; 6.2) | 3.6 (1.8; 7.3) | 4.0 (1.5; 8.4) |
| CGA pre-frail | 1.2 (1.0; 1.4) | 1.9 (1.4; 2.5) | 2.4 (1.7; 3.5) | 2.7 (1.8; 3.9) | 2.9 (2.5; 4.4) | 3.3 (1.7; 5.3) | 3.8 (1.3; 6.1) | 4.1 (1.4; 7.0) |
| CGAST frail | 1.7 (1.2; 2.3) | 2.4 (1.4; 4.2) | 3.1 (1.6; 5.9) | 3.3 (1.6; 6.7) | 3.6 (4.2; 7.6) | 4.0 (1.6; 9.2) | 4.5 (1.9; 10.7) | 4.9 (2.3; 12.3) |
| CGAST pre frail | 1.4 (1.0; 1.9) | 2.0 (1.3; 3.4) | 2.6 (1.4; 4.7) | 2.8 (1.4; 5.4) | 3.0 (3.4; 6.0) | 3.4 (1.4; 7.2) | 3.7 (1.7; 8.4) | 4.1 (1.9; 9.6) |
| CSBA frail | 1.1 (0.9; 1.3) | 1.6 (1.1; 2.2) | 1.9 (1.3; 3.0) | 2.1 (1.3; 3.4) | 2.3 (2.2; 3.7) | 2.6 (1.3; 4.4) | 2.8 (1.6; 5.0) | 3.0 (1.3; 5.6) |
| EFS frail | 1.6 (1.2; 2.2) | 1.9 (1.1; 3.2) | 2.0 (1.0; 4.0) | 2.1 (1.0; 4.3) | 2.1 (3.2; 4.6) | 2.3 (1.0; 5.2) | 2.3 (1.0; 5.7) | 2.4 (2.2; 6.2) |
| FI40 frail | 1.1 (0.9; 1.4) | 1.6 (1.1; 2.2) | 1.9 (1.3; 2.8) | 2.0 (1.3; 3.1) | 2.2 (2.2; 3.4) | 2.4 (1.3; 3.9) | 2.6 (1.5; 4.4) | 2.8 (1.4; 4.9) |
| FI70 frail | 1.2 (1.0; 1.4) | 1.6 (1.2; 2.2) | 1.9 (1.3; 2.9) | 2.1 (1.3; 3.2) | 2.2 (2.2; 3.5) | 2.4 (1.3; 4.1) | 2.7 (1.5; 4.6) | 2.8 (1.4; 5.1) |
| FiND frail | 1.0 (0.7; 1.3) | 1.3 (0.8; 2.2) | 1.6 (0.9; 3.0) | 1.7 (0.9; 3.3) | 1.8 (2.2; 3.7) | 2.0 (0.9; 4.4) | 2.2 (1.0; 5.0) | 2.4 (1.3; 5.6) |
| FS frail | 1.3 (1.0; 1.7) | 1.9 (1.2; 3.1) | 2.5 (1.4; 4.4) | 2.7 (1.5; 5.1) | 3.0 (3.1; 5.7) | 3.4 (1.4; 7.0) | 3.8 (1.8; 8.2) | 4.2 (1.7; 9.4) |
| FS pre- frail | 1.1 (1.0; 1.4) | 1.8 (1.3; 2.3) | 2.3 (1.6; 3.2) | 2.5 (1.7; 3.6) | 2.7 (2.3; 4.0) | 3.1 (1.6; 4.8) | 3.5 (1.2; 5.5) | 3.8 (1.4; 6.2) |
| FSS frail | 0.9 (0.7; 1.1) | 1.4 (0.9; 2.2) | 1.9 (1.1; 3.2) | 2.1 (1.2; 3.7) | 2.3 (2.2; 4.3) | 2.7 (1.1; 5.3) | 3.1 (0.5; 6.2) | 3.4 (1.1; 7.2) |
| FSS pre frail | 1.2 (1.0; 1.4) | 1.9 (1.4; 2.5) | 2.5 (1.7; 3.6) | 2.8 (1.9; 4.1) | 3.0 (2.5; 4.6) | 3.5 (1.7; 5.6) | 4.0 (1.5; 6.5) | 4.5 (1.4; 7.5) |
| G8 frail | 1.3 (1.0; 1.6) | 1.8 (1.2; 2.7) | 2.2 (1.4; 3.7) | 2.4 (1.4; 4.1) | 2.6 (2.7; 4.5) | 2.9 (1.4; 5.3) | 3.2 (1.7; 6.1) | 3.4 (1.6; 6.8) |
| GFI frail | 1.0 (0.8; 1.2) | 1.3 (1.0; 1.9) | 1.6 (1.1; 2.4) | 1.7 (1.1; 2.7) | 1.9 (1.9; 3.0) | 2.1 (1.1; 3.5) | 2.3 (1.3; 3.9) | 2.4 (1.2; 4.3) |
| HRCA frail | 1.1 (0.9; 1.3) | 1.5 (1.1; 2.1) | 1.8 (1.2; 2.8) | 2.0 (1.3; 3.1) | 2.1 (2.1; 3.4) | 2.3 (1.2; 3.9) | 2.5 (1.5; 4.4) | 2.7 (1.3; 4.9) |
| IFQ frail | 1.3 (0.8; 2.0) | 1.7 (0.8; 3.5) | 1.9 (0.8; 4.9) | 2.1 (0.8; 5.5) | 2.2 (3.5; 6.2) | 2.4 (0.8; 7.4) | 2.5 (1.7; 8.6) | 2.7 (2.0; 9.7) |
| MFS frail | 1.5 (0.9; 2.4) | 2.2 (1.0; 4.9) | 2.8 (1.5; 7.6) | 3.1 (1.1; 9.0) | 3.4 (4.9; 10.3) | 3.9 (1.1; 13.1) | 4.3 (1.2; 15.9) | 4.7 (2.4; 18.7) |
| MFS pre-frail | 1.2 (0.8; 1.9) | 1.8 (0.8; 3.8) | 2.3 (0.9; 5.9) | 2.5 (0.9; 6.9) | 2.7 (3.8; 7.9) | 3.1 (0.9; 10.0) | 3.5 (1.0; 12.1) | 3.8 (1.9; 14.2) |
| PFI frail | 1.0 (0.7; 1.4) | 1.6 (0.9; 2.8) | 2.0 (1.0; 4.2) | 2.3 (1.0; 4.9) | 2.5 (2.8; 5.7) | 2.9 (1.0; 7.1) | 3.2 (1.2; 8.5) | 3.6 (1.4; 10.0) |
| PFI pre frail | 1.1 (0.9; 1.4) | 1.8 (1.3; 2.5) | 2.3 (1.6; 3.5) | 2.6 (1.7; 4.0) | 2.8 (2.5; 4.4) | 3.3 (1.6; 5.4) | 3.7 (1.2; 6.3) | 4.1 (1.4; 7.2) |
| PHF frail | 1.5 (1.0; 2.2) | 2.4 (1.2; 4.5) | 3.1 (1.4; 6.9) | 3.4 (1.4; 8.0) | 3.7 (4.5; 9.2) | 4.3 (1.4; 11.6) | 4.8 (1.7; 14.0) | 5.3 (2.2; 16.4) |
| PHF pre-frail | 1.1 (0.8; 1.5) | 1.7 (1.0; 3.1) | 2.2 (1.1; 4.6) | 2.5 (1.2; 5.3) | 2.7 (3.1; 6.1) | 3.1 (1.1; 7.6) | 3.5 (1.4; 9.1) | 3.9 (1.5; 10.6) |
| SDFI frail | 1.0 (0.8; 1.2) | 1.4 (1.0; 2.0) | 1.8 (1.2; 2.8) | 1.9 (1.2; 3.1) | 2.1 (2.0; 3.4) | 2.4 (1.2; 4.1) | 2.6 (1.5; 4.6) | 2.8 (1.2; 5.2) |
| SHCFS frail | 1.2 (0.9; 1.5) | 1.5 (1.0; 2.2) | 1.7 (1.1; 2.8) | 1.8 (1.1; 3.0) | 1.9 (2.2; 3.3) | 2.1 (1.1; 3.7) | 2.2 (1.2; 4.1) | 2.3 (1.5; 4.5) |
| SI frail | 0.7 (0.5; 1.1) | 1.0 (0.5; 2.1) | 1.3 (0.5; 3.1) | 1.4 (0.5; 3.7) | 1.5 (2.1; 4.2) | 1.7 (0.5; 5.2) | 1.9 (0.6; 6.2) | 2.1 (1.1; 7.2) |
| SOF frail | 1.3 (1.0; 1.7) | 1.9 (1.2; 3.0) | 2.4 (1.4; 4.2) | 2.6 (1.4; 4.7) | 2.8 (3.0; 5.2) | 3.1 (1.4; 6.3) | 3.4 (1.6; 7.3) | 3.7 (1.7; 8.2) |
| SOF pre-frail | 1.3 (1.1; 1.5) | 1.8 (1.4; 2.4) | 2.3 (1.6; 3.2) | 2.5 (1.7; 3.6) | 2.7 (2.4; 4.0) | 3.0 (1.6; 4.6) | 3.3 (1.1; 5.3) | 3.6 (1.5; 5.9) |
| SPPB frail | 1.0 (0.8; 1.2) | 1.4 (0.9; 2.0) | 1.7 (1.1; 2.8) | 1.8 (1.1; 3.1) | 2.0 (2.0; 3.4) | 2.2 (1.1; 4.0) | 2.4 (1.3; 4.6) | 2.6 (1.2; 5.1) |
| SPQ frail | 0.7 (0.6; 0.8) | 0.9 (0.8; 1.2) | 1.2 (0.9; 1.5) | 1.2 (0.9; 1.7) | 1.3 (1.2; 1.8) | 1.5 (0.9; 2.1) | 1.6 (0.1; 2.3) | 1.7 (0.8; 2.6) |
| TFI frail | 1.1 (0.9; 1.4) | 1.6 (1.1; 2.2) | 1.9 (1.3; 2.9) | 2.1 (1.3; 3.2) | 2.2 (2.2; 3.6) | 2.5 (1.3; 4.1) | 2.7 (1.6; 4.7) | 2.9 (1.4; 5.2) |
| VES13 frail | 1.0 (0.8; 1.2) | 1.4 (1.0; 2.0) | 1.7 (1.1; 2.7) | 1.9 (1.2; 3.0) | 2.0 (2.0; 3.3) | 2.3 (1.1; 3.8) | 2.5 (1.4; 4.3) | 2.7 (1.2; 4.8) |
| WHRH frail | 1.1 (0.9; 1.3) | 1.4 (1.0; 2.1) | 1.7 (1.1; 2.6) | 1.8 (1.1; 2.9) | 1.9 (2.1; 3.1) | 2.1 (1.1; 3.6) | 2.2 (1.2; 4.0) | 2.4 (1.3; 4.4) |
| ZED1 frail | 1.4 (1.0; 2.0) | 1.7 (1.0; 3.0) | 1.9 (1.0; 3.8) | 2.0 (1.0; 4.2) | 2.1 (3.0; 4.5) | 2.2 (1.0; 5.2) | 2.3 (1.9; 5.7) | 2.4 (2.0; 6.3) |
| ZED2 frail | 1.5 (1.1; 2.2) | 1.9 (1.0; 3.4) | 2.1 (1.0; 4.4) | 2.2 (1.0; 4.9) | 2.2 (3.4; 5.3) | 2.4 (1.0; 6.1) | 2.5 (1.9; 6.9) | 2.6 (2.2; 7.6) |
| ZED3 frail | 1.1 (0.5; 2.4) | 1.7 (0.4; 6.7) | 2.2 (0.4; 12.1) | 2.4 (0.4; 15.2) | 2.6 (6.7; 18.5) | 3.0 (0.4; 25.6) | 3.4 (1.3; 33.4) | 3.8 (2.4; 41.9) |

^1^Hazard ratios calculated from age at baseline to age at the end of the interval.

BFI= Brief Frailty Index. CGA= Comprehensive Geriatric Assessment. CGAST= Comprehensive Geriatric Assessment Screening Tests. CSBA= Conselice Study of Brain Aging Score. EFS= Edmonton Frail Scale. FI40= 40-item Frailty Index. FI70= 70-item Frailty Index. FIND= Frail Non-Disabled Questionnaire. FS= Frail Scale. FSS= Frailty Staging System. G8= G-8 Geriatric Screening Tool. GFI= Groningen Frailty Indicator. HRCA= Hebrew Rehabilitation Center for Aged Vulnerability Index. IFQ= Inter-Frail Questionnaire. MFS= Modified Frailty Score. PFI= Physical Frailty Index. PHF= Phenotype of Frailty. SDFI=, Static/Dynamic Frailty Index. SHCFS= Canadian Study of Health and Aging Clinical Frailty Scale·. SI= Screening Instrument. SOF= Study of Osteoporotic Fractures. SPPB= Short Physical Performance Battery. SPQ= Sherbrooke Postal Questionnaire. TFI= Tilburg Frailty Indicator. VES13= Vulnerable Elders Survey. WHRH= WHOAFC & self-reported health. ZED1= ZutPhen Elderly Study (Physical Activity & Low Energy). ZED2= ZutPhen Elderly Study (Physical Activity & Weight Loss). ZED3= ZutPhen Elderly Study (Physical Activity & Low BMI).
