# Supplementary material for: Comparative analysis of the association between 35 frailty scores and cardiovascular events, cancer, and total mortality in an elderly general population in England: An observational study
Source: PLoS Med. 2018 Mar 27;15(3):e1002543. doi: 10.1371/journal.pmed.1002543 (PMC5870943; doi:10.1371/journal.pmed.1002543)
Supplement: S4 Table — (DOCX) [file pmed.1002543.s005.docx]

**S4 Table.** Cardiovascular hazard ratios of frailty scores (n=4554) calculated at median time follow-up (2.5 years)

| **Continuous analysis** | | | | | **Cut-off analysis** | | | | |
| --- | --- | --- | --- | --- | --- | --- | --- | --- | --- |
|  | **HR (95% CI)** | **HR (95% CI)** | **HR (95% CI)** | **HR (95% CI)** |  | **HR (95% CI)** | **HR (95% CI)** | **HR (95% CI)** | **HR (95% CI)** |
| **Frailty Score** | **Model 0^1^** | **Model 1^2^** | **Model 2^3^** | **Model 3^4^** | **Frailty Score** | **Model 0^1^** | **Model 1^2^** | **Model 2^3^** | **Model 3^4^** |
| **Phenotype of frailty approach** | | | | | | | | | |
| SPPB | 2.5 (1.1; 5.5) | 2.8 (1.2; 6.3) | 2.6 (1.2; 5.9) | 1.4 (0.6; 3.4) | PFI frail | 1.7 (0.4; 6.7) | 1.8 (0.4; 7.1) | 1.7 (0.4; 6.9) | 1.3 (0.4; 5.7) |
| MPHF | 2.3 (1.1; 4.6) | 2.5 (1.2; 5.0) | 2.2 (1.1; 4.4) | 1.4 (0.6; 3.1) | PFI pre frail | 2.4 (1.2; 4.8) | 2.5 (1.2; 5.1) | 2.4 (1.2; 4.8) | 2.0 (1.2; 4.2) |
| FS | 2.1 (0.9; 5.1) | 2.3 (1.0; 5.5) | 2.1 (0.9; 5.0) | 1.2 (0.5; 3.1) | PHF frail | 1.8 (0.5; 6.2) | 1.9 (0.5; 6.6) | 1.7 (0.5; 6.0) | 8.1 (5.3; 12.4) |
| BDE | 2.2 (1.2; 4.1) | 2.3 (1.2; 4.2) | 2.1 (1.1; 3.8) | 1.3 (0.7; 2.5) | PHF pre-frail | 2.2 (0.8; 5.9) | 2.3 (0.8; 6.2) | 2.2 (0.8; 5.9) | 0.1 (0.0; 7.9) |
| PHF | 2.1 (1.0; 4.1) | 2.2 (1.1; 4.4) | 2.0 (1.0; 4.0) | 1.2 (0.6; 2.6) | FS frail | 1.5 (0.5; 4.8) | 1.6 (0.5; 4.9) | 1.5 (0.5; 4.7) | 1.0 (0.5; 3.5) |
| FiND | 2.0 (0.9; 4.2) | 2.1 (1.0; 4.5) | 2.0 (0.9; 4.2) | 1.1 (0.5; 2.7) | FS pre- frail | 2.2 (1.2; 4.0) | 2.3 (1.2; 4.1) | 2.2 (1.2; 3.9) | 1.9 (1.2; 3.6) |
| PFI | 1.9 (1.0; 3.5) | 2.0 (1.1; 3.8) | 1.9 (1.0; 3.6) | 1.4 (0.7; 2.8) | SOF frail | 1.4 (0.4; 4.3) | 1.4 (0.4; 4.5) | 1.4 (0.4; 4.2) | 1.0 (0.4; 3.5) |
| SOF | 1.6 (0.7; 3.6) | 1.7 (0.7; 3.8) | 1.6 (0.7; 3.5) | 1.0 (0.4; 2.5) | SOF pre-frail | 2.0 (1.1; 3.7) | 2.1 (1.1; 3.8) | 2.0 (1.1; 3.7) | 1.8 (1.1; 3.4) |
| ZED1 | 1.4 (0.8; 2.7) | 1.6 (0.8; 3.0) | 1.5 (0.8; 2.8) | 1.0 (0.5; 2.0) | ZED2 frail | 1.5 (0.2; 11.2) | 1.5 (0.2; 11.3) | 1.5 (0.2; 11.1) | 1.1 (0.2; 8.5) |
| ZED2 | 1.3 (0.7; 2.6) | 1.4 (0.7; 2.7) | 1.3 (0.6; 2.5) | 0.9 (0.4; 1.8) | FiND frail | 1.3 (0.5; 3.3) | 1.4 (0.5; 3.4) | 1.3 (0.5; 3.3) | 1.1 (0.5; 2.8) |
| ZED3 | 1.1 (0.5; 2.5) | 1.2 (0.6; 2.8) | 1.1 (0.5; 2.5) | 0.9 (0.4; 2.1) | SPPB frail | 1.3 (0.6; 2.6) | 1.3 (0.6; 2.7) | 1.3 (0.6; 2.6) | 1.0 (0.6; 2.1) |
|  |  |  |  |  | ZED1 frail | 1.2 (0.2;5.4) | 1.2 (0.3; 5.4) | 1.2 (0.2;5.4) | 0.8 (0.2; 3.7) |
|  |  |  |  |  | ZED3 frail | 0.6 (0.0; 363.5) | 0.6 (0.0; 382.6) | 0.6 (0.0; 430.5) | 0.5 (0.0; 314.9) |
| **Multidimensional approach** | | | | | | | | | |
| EFS | 4.7 (1.3; 16.5) | 5.6 (1.6; 19.6) | 4.6 (1.3; 16.4) | 5.1 (1.2; 20.7) | CGAST frail | 2.0 (0.7; 5.3) | 2.1 (0.7; 5.7) | 2.0 (0.7; 5.4) | 1.5 (0.7; 4.3) |
| CSBA | 4.3 (1.6; 11.5) | 3.7 (1.3; 10.4) | 3.0 (1.1; 8.4) | 0.6 (0.2; 2.0) | CGAST pre frail | 2.7 (1.2; 6.2) | 2.7 (1.2; 6.3) | 2.7 (1.2; 6.2) | 2.5 (1.2; 5.9) |
| HSF | 2.9 (1.2; 7.0) | 3.1 (1.3; 7.5) | 2.9 (1.2; 6.9) | 1.5 (0.5; 4.0) | MFS frail | 1.6 (0.4; 6.1) | 1.6 (0.4; 6.3) | 1.5 (0.4; 5.7) | 1.2 (0.4; 4.7) |
| G8 | 2.7 (0.9; 7.8) | 3.1 (1.1; 9.1) | 2.6 (0.9; 7.6) | 0.8 (0.2; 2.8) | MFS pre-frail | 2.0 (0.6; 6.8) | 2.1 (0.6; 7.0) | 1.9 (0.6; 6.5) | 1.8 (0.6; 6.0) |
| SDFI | 2.1 (0.9; 5.1) | 3.1 (1.3; 7.5) | 2.5 (1.0; 6.1) | 1.0 (0.4; 2.7) | FSS frail | 1.3 (0.5; 3.4) | 1.4 (0.5; 3.5) | 1.3 (0.5; 3.4) | 0.9 (0.5; 2.5) |
| GFI | 2.6 (0.9; 7.3) | 3.0 (1.1; 8.5) | 2.6 (0.9; 7.4) | 1.2 (0.3; 3.9) | FSS pre frail | 2.0 (1.1; 3.5) | 2.0 (1.1; 3.6) | 1.9 (1.1; 3.4) | 1.6 (1.1; 3.0) |
| CGAST | 2.3 (0.8; 6.7) | 2.7 (0.9; 7.9) | 2.3 (0.8; 6.9) | 1.0 (0.3; 3.2) | EFS frail | 1.7 (0.4; 7.5) | 1.8 (0.4; 7.8) | 1.6 (0.4; 7.2) | 1.4 (0.4; 6.6) |
| MFS | 2.3 (1.1; 4.9) | 2.5 (1.2; 5.1) | 2.1 (1.0; 4.5) | 1.5 (0.7; 3.3) | G8 frail | 1.6 (0.8; 3.5) | 1.7 (0.8; 3.7) | 1.6 (0.8; 3.5) | 1.2 (0.8; 2.8) |
| TFI | 1.9 (0.8; 4.4) | 2.2 (0.9; 5.4) | 1.8 (0.8; 4.5) | 1.1 (0.4; 3.1) | IFQ frail | 1.5 (0.3; 8.8) | 1.6 (0.3; 9.2) | 1.5 (0.3; 8.5) | 1.2 (0.3; 6.8) |
| FSS | 1.8 (0.8; 3.7) | 1.9 (0.9; 4.0) | 1.8 (0.8; 3.7) | 1.0 (0.4; 2.2) | SDFI frail | 1.3 (0.6; 2.6) | 1.4 (0.6; 2.9) | 1.3 (0.6; 2.7) | 1.0 (0.6; 2.2) |
| IFQ | 1.3 (0.5; 3.7) | 1.5 (0.5; 4.3) | 1.3 (0.5; 3.6) | 0.5 (0.2; 1.5) | TFI frail | 1.3 (0.6; 2.5) | 1.3 (0.6; 2.6) | 1.2 (0.6; 2.5) | 1.1 (0.6; 2.2) |
| BFI | 1.2 (0.5; 2.6) | 1.4 (0.7; 3.2) | 1.2 (0.6; 2.7) | 0.7 (0.3; 1.6) | CSBA frail | 1.4 (0.7; 2.7) | 1.3 (0.7; 2.6) | 1.2 (0.7; 2.5) | 0.9 (0.7; 1.9) |
| SI | 1.2 (0.4; 3.6) | 1.4 (0.5; 4.2) | 1.3 (0.4; 3.9) | 0.6 (0.2; 1.8) | GFI frail | 1.2 (0.6; 2.6) | 1.3 (0.6; 2.7) | 1.2 (0.6; 2.6) | 1.0 (0.6; 2.2) |
| SPQ | 1.0 (0.4; 2.8) | 1.2 (0.5; 3.2) | 1.1 (0.4; 2.8) | 0.6 (0.2; 1.7) | SI frail | 1.1 (0.2; 5.7) | 1.2 (0.2; 5.9) | 1.1 (0.2; 5.7) | 0.8 (0.2; 4.4) |
|  |  |  |  |  | BFI frail | 0.9 (0.2; 3.4) | 0.9 (0.2; 3.7) | 0.9 (0.2; 3.4) | 0.7 (0.2; 2.8) |
|  |  |  |  |  | SPQ frail | 1.0 (0.4; 2.1) | 0.6 (0.4; 1.0) | 0.6 (0.4; 1.0) | 0.8 (0.4; 1.8) |
| **Accumulation of deficits approach** | | | | | | | | | |
| FI40 | 12.7 (6.0; 26.8) | 16.5 (7.8; 35.0) | 14.3 (6.6; 30.9) | 15.2 (5.8; 40.3) | CGA frail | 1.5 (0.6; 4.0) | 1.7 (0.6; 4.4) | 1.6 (0.6; 4.2) | 1.5 (0.6; 4.2) |
| FI70 | 5.1 (1.6; 15.9) | 7.1 (2.3; 22.2) | 6.2 (2.0; 19.6) | 6.9 (1.8; 26.3) | CGA pre-frail | 2.1 (1.1; 3.7) | 2.2 (1.1; 4.0) | 2.1 (1.1; 3.9) | 2.1 (1.1; 3.9) |
| CGA | 4.1 (1.0; 17.1) | 5.8 (1.4; 24.2) | 4.9 (1.2; 20.9) | 4.2 (0.8; 20.8) | FI70 frail | 1.6 (0.8; 3.2) | 1.7 (0.8; 3.5) | 1.6 (0.8; 3.3) | 1.5 (0.8; 3.3) |
| EFIP | 4.6 (1.5; 14.6) | 5.6 (1.8; 17.8) | 4.9 (1.5; 15.6) | 4.9 (1.3; 18.6) | FI40 frail | 1.5 (0.8; 3.1) | 1.7 (0.8; 3.4) | 1.6 (0.8; 3.2) | 2.1 (0.8; 3.6) |
| NLTCS | 4.7 (0.9; 23.7) | 5.3 (1.0; 26.8) | 4.7 (0.9; 23.8) | 1.5 (0.2; 9.4) |  |  |  |  |  |
| FIBLSA | 4.0 (1.1; 14.5) | 4.8 (1.3; 17.2) | 4.3 (1.2; 15.5) | 2.1 (0.5; 9.3) |  |  |  |  |  |
| **Disability approach** | | | | | | | | | |
| VES13 | 3.1 (1.3; 7.4) | 3.7 (1.6; 8.7) | 3.4 (1.4; 8.0) | 2.6 (1.0; 6.9) | HRCA frail | 1.4 (0.7; 3.0) | 1.6 (0.7; 3.3) | 1.5 (0.7; 3.2) | 1.2 (0.7; 2.7) |
| HRCA | 2.7 (1.0; 7.3) | 3.3 (1.2; 8.8) | 3.0 (1.1; 8.1) | 1.5 (0.5; 4.7) | VES13 frail | 1.4 (0.7; 2.9) | 1.5 (0.7; 3.1) | 1.4 (0.7; 3.0) | 1.2 (0.7; 2.7) |
| WHRH | 2.5 (1.1; 6.1) | 2.9 (1.2; 7.0) | 2.8 (1.2; 6.6) | 2.2 (0.8; 5.8) | WHRH frail | 1.4 (0.6; 3.2) | 1.5 (0.6; 3.3) | 1.4 (0.6; 3.3) | 1.0 (0.6; 2.5) |
| SHCFS | 1.7 (0.8; 3.6) | 1.8 (0.9; 3.9) | 1.7 (0.8; 3.7) | 0.9 (0.4; 2.2) | SHCFS frail | 1.4 (0.5; 3.6) | 1.4 (0.5; 3.7) | 1.4 (0.5; 3.6) | 1.0 (0.5; 2.9) |

^1^Model 0= Crude models. 2Model 1= HR adjusted by sex. 3Model 2= Model 1 + smoking status and alcohol consumption. 4Model 3= Model 2 + physical activity, BMI, diabetes, hypertension, cancer, anaemia, COPD, arthritis, neuropsychiatric, depression, cognition, self-rated health & quality of life.

Abbreviations frailty scores: BDE= Beaver Dam Eye Study Index. BFI= Brief Frailty Index. CGA= Comprehensive Geriatric Assessment. CGAST= Comprehensive Geriatric Assessment Screening Tests. CSBA= Conselice Study of Brain Aging Score. EFIP= Evaluative Frailty Index for Physical Activity. EFS= Edmonton Frail Scale. FI40= 40-item Frailty Index. FI70= 70-item Frailty Index (SHARE). FIBLSA= Frailty Index Beijing Longitudinal Study of Ageing. FiND= Frail Non-Disabled Questionnaire. FS= Frail Scale. FSS= Frailty Staging System. G8= G-8 Geriatric Screening Tool. GFI= Groningen Frailty Indicator. HRCA= Hebrew Rehabilitation Center for Aged Vulnerability Index. HSF= Health Status Form. IFQ= Inter-Frail Questionnaire. MFS= Modified Frailty Score. MPHF= Modified Phenotype of Frailty. NLTCS= Long Term Care Survey Frailty Index. PFI= Physical Frailty Index. PHF= Phenotype of Frailty. SDFI=, Static/Dynamic Frailty Index. SHCFS= Canadian Study of Health and Aging Clinical Frailty Scale·. SI= Screening Instrument. SOF= Study of Osteoporotic Fractures. SPPB= Short Physical Performance Battery. SPQ= Sherbrooke Postal Questionnaire. TFI= Tilburg Frailty Indicator. VES13= Vulnerable Elders Survey. WHRH= WHOAFC & self-reported health. ZED1= ZutPhen Elderly Study (Physical Activity & Low Energy). ZED2= ZutPhen Elderly Study (Physical Activity & Weight Loss). ZED3= ZutPhen Elderly Study (Physical Activity & Low BMI).
