# Supplementary material for: Comparative analysis of the association between 35 frailty scores and cardiovascular events, cancer, and total mortality in an elderly general population in England: An observational study
Source: PLoS Med. 2018 Mar 27;15(3):e1002543. doi: 10.1371/journal.pmed.1002543 (PMC5870943; doi:10.1371/journal.pmed.1002543)
Supplement: S5 Table — (DOCX) [file pmed.1002543.s006.docx]

**S5 Table.** Cardiovascular hazard ratios of frailty scores assessed in intervals from1 to 7 years^1^: age-adjusted model and continuous analysis

| Scores | HR1 (LCI; UCI) | HR2 (LCI; UCI) | HR2.5 (LCI; UCI) | HR3 (LCI; UCI) | HR4 (LCI; UCI) | HR5 (LCI; UCI) | HR6 (LCI; UCI) | HR7 (LCI; UCI) |
| --- | --- | --- | --- | --- | --- | --- | --- | --- |
| BDE | 0.7 (0.4; 1.2) | 1.7 (0.9; 3.1) | 2.2 (1.2; 4.1) | 2.8 (1.5; 5.3) | 4.2 (2.3; 7.8) | 5.6 (3.1; 10.5) | 7.2 (3.9; 13.4) | 8.9 (4.8; 16.4) |
| BFI | 0.2 (0.1; 0.5) | 0.8 (0.4; 1.7) | 1.2 (0.5; 2.6) | 1.7 (0.8; 3.6) | 2.8 (1.3; 6.1) | 4.2 (1.7; 9.1) | 5.8 (2.7; 12.6) | 7.7 (3.5; 16.7) |
| CGA | 0.2 (0.1; 1.0) | 2.0 (0.5; 8.4) | 4.1 (1.0; 17.1) | 7.2 (1.7; 30.3) | 17.9 (4.3; 74.9) | 36.2 (8.4; 151.2) | 64.2 (15.3; 268.5) | 104.3 (24.9; 436.2) |
| CGAST | 0.2 (0.1; 0.6) | 1.3 (0.4; 3.8) | 2.3 (0.6; 6.7) | 3.6 (1.2; 10.8) | 7.7 (2.6; 22.9) | 13.8 (3.8; 40.9) | 22.1 (7.4; 65.7) | 33.0 (11.1; 98.1) |
| CSBA | 0.7 (0.3; 1.9) | 2.7 (0.0; 7.4) | 4.3 (1.9; 11.5) | 6.1 (2.3; 16.6) | 10.9 (4.0; 29.4) | 17.0 (7.4; 45.9) | 24.4 (9.0; 66.0) | 33.2 (12.3; 89.8) |
| EFIP | 0.4 (0.1; 1.3) | 2.6 (0.8; 8.1) | 4.6 (1.3; 14.6) | 7.4 (2.3; 23.4) | 15.7 (4.9; 49.5) | 28.0 (8.1; 88.5) | 45.0 (14.2; 142.3) | 67.2 (21.2; 212.6) |
| EFS | 0.3 (0.1; 1.2) | 2.5 (0.7; 8.7) | 4.7 (1.2; 16.5) | 7.9 (2.3; 27.8) | 18.0 (5.1; 63.3) | 34.1 (8.7; 119.8) | 57.5 (16.4; 201.7) | 89.3 (25.5; 313.5) |
| FI40 | 17.9 (8.5; 37.8) | 13.8 (17.5; 29.2) | 12.7 (37.8; 26.8) | 11.9 (5.6; 25.1) | 10.7 (5.0; 22.5) | 9.8 (29.2; 20.7) | 9.2 (4.3; 19.3) | 8.6 (4.1; 18.2) |
| FI70 | 0.5 (0.2; 1.5) | 2.9 (0.9; 9.0) | 5.1 (1.5; 15.9) | 8.1 (2.6; 25.2) | 16.9 (5.4; 52.4) | 29.7 (9.0; 92.5) | 47.3 (15.2; 147.1) | 70.0 (22.5; 217.7) |
| FIBLSA | 0.3 (0.1; 1.1) | 2.1 (0.6; 7.7) | 4.0 (1.1; 14.5) | 6.7 (1.9; 24.2) | 15.1 (4.2; 54.5) | 28.4 (7.7; 102.4) | 47.5 (13.2; 171.2) | 73.4 (20.3; 264.5) |
| FiND | 0.5 (0.2; 1.0) | 1.4 (0.7; 2.9) | 2.0 (1.0; 4.2) | 2.6 (1.2; 5.6) | 4.1 (2.0; 8.8) | 5.9 (2.9; 12.5) | 7.9 (3.7; 16.7) | 10.0 (4.7; 21.3) |
| FS | 0.5 (0.2; 1.2) | 1.5 (0.6; 3.5) | 2.1 (1.2; 5.1) | 2.9 (1.2; 6.8) | 4.5 (1.9; 10.7) | 6.5 (3.5; 15.4) | 8.7 (3.7; 20.6) | 11.1 (4.7; 26.3) |
| FSS | 0.4 (0.2; 0.8) | 1.2 (0.6; 2.5) | 1.8 (0.8; 3.7) | 2.4 (1.1; 5.0) | 3.9 (1.8; 8.1) | 5.6 (2.5; 11.8) | 7.6 (3.6; 16.0) | 9.9 (4.7; 20.8) |
| G8 | 0.3 (0.1; 0.8) | 1.5 (0.5; 4.5) | 2.7 (0.8; 7.8) | 4.2 (1.4; 12.2) | 8.6 (2.9; 24.9) | 14.9 (4.5; 43.3) | 23.4 (8.1; 68.1) | 34.3 (11.8; 99.8) |
| GFI | 0.3 (0.1; 0.9) | 1.6 (0.6; 4.4) | 2.6 (0.9; 7.3) | 4.0 (1.4; 11.2) | 7.8 (2.8; 21.9) | 13.1 (4.4; 36.8) | 20.1 (7.2; 56.3) | 28.8 (10.3; 80.6) |
| HRCA | 0.4 (0.2; 1.1) | 1.7 (0.6; 4.6) | 2.7 (1.1; 7.3) | 3.9 (1.5; 10.5) | 7.0 (2.6; 18.9) | 11.0 (4.6; 29.7) | 16.0 (5.9; 43.0) | 21.8 (8.1; 58.8) |
| HSF | 0.5 (0.2; 1.1) | 1.9 (0.8; 4.5) | 2.9 (1.1; 7.0) | 4.2 (1.8; 10.1) | 7.4 (3.1; 17.8) | 11.5 (4.5; 27.6) | 16.6 (6.9; 39.6) | 22.4 (9.4; 53.6) |
| IFQ | 0.2 (0.1; 0.5) | 0.8 (0.3; 2.3) | 1.3 (0.5; 3.7) | 2.0 (0.7; 5.7) | 3.9 (1.4; 10.9) | 6.4 (2.3; 18.1) | 9.8 (3.5; 27.5) | 13.8 (4.9; 39.0) |
| MFS | 0.5 (0.3; 1.1) | 1.6 (0.8; 3.4) | 2.3 (1.1; 4.9) | 3.1 (1.5; 6.5) | 5.0 (2.4; 10.4) | 7.2 (3.4; 15.0) | 9.6 (4.6; 20.2) | 12.4 (5.9; 25.9) |
| MPHF | 0.5 (0.3; 1.0) | 1.6 (0.8; 3.2) | 2.3 (1.0; 4.6) | 3.0 (1.5; 6.1) | 4.9 (2.4; 9.8) | 7.0 (3.2; 14.0) | 9.4 (4.7; 18.9) | 12.1 (6.0; 24.3) |
| NLTCS | 0.2 (0.0; 1.0) | 2.1 (0.4; 10.8) | 4.7 (1.0; 23.7) | 8.8 (1.7; 44.9) | 24.2 (4.7; 123.1) | 52.8 (10.8; 269.0) | 100.0 (19.6; 509.6) | 171.7 (33.7; 874.6) |
| PFI | 0.7 (0.4; 1.4) | 1.5 (0.8; 2.8) | 1.9 (1.4; 3.5) | 2.2 (1.2; 4.2) | 3.0 (1.6; 5.7) | 3.8 (2.8; 7.1) | 4.6 (2.4; 8.6) | 5.3 (2.8; 10.1) |
| PHF | 0.5 (0.2; 1.0) | 1.4 (0.7; 2.9) | 2.1 (1.0; 4.1) | 2.8 (1.4; 5.5) | 4.3 (2.2; 8.7) | 6.2 (2.9; 12.4) | 8.3 (4.2; 16.5) | 10.6 (5.3; 21.1) |
| SDFI | 0.4 (0.2; 1.0) | 1.4 (0.6; 3.4) | 2.1 (1.0; 5.1) | 2.9 (1.2; 7.1) | 5.0 (2.1; 12.0) | 7.5 (3.4; 18.0) | 10.4 (4.3; 25.0) | 13.7 (5.7; 33.1) |
| SHCFS | 0.4 (0.2; 0.8) | 1.2 (0.5; 2.5) | 1.7 (0.8; 3.6) | 2.3 (1.1; 5.0) | 3.8 (1.8; 8.1) | 5.6 (2.5; 11.9) | 7.6 (3.6; 16.2) | 9.9 (4.6; 21.1) |
| SI | 0.1 (0.0; 0.4) | 0.7 (0.2; 2.1) | 1.2 (0.4; 3.6) | 1.8 (0.6; 5.6) | 3.7 (1.2; 11.2) | 6.4 (2.1; 19.3) | 9.9 (3.3; 30.0) | 14.4 (4.7; 43.7) |
| SOF | 0.4 (0.2; 0.9) | 1.1 (0.5; 2.6) | 1.6 (0.9; 3.6) | 2.1 (0.9; 4.7) | 3.2 (1.4; 7.3) | 4.5 (2.6; 10.2) | 6.0 (2.7; 13.5) | 7.6 (3.4; 17.1) |
| SPPB | 0.5 (0.2; 1.2) | 1.7 (0.8; 3.8) | 2.5 (1.2; 5.5) | 3.3 (1.5; 7.5) | 5.4 (2.4; 12.1) | 7.9 (3.8; 17.7) | 10.7 (4.8; 24.0) | 13.9 (6.2; 31.1) |
| SPQ | 0.2 (0.1; 0.4) | 0.7 (0.2; 1.7) | 1.0 (0.4; 2.8) | 1.5 (0.6; 4.0) | 2.8 (1.1; 7.4) | 4.5 (1.7; 11.8) | 6.6 (2.5; 17.3) | 9.1 (3.4; 23.9) |
| TFI | 0.3 (0.1; 0.8) | 1.2 (0.5; 2.9) | 1.9 (0.8; 4.4) | 2.6 (1.1; 6.2) | 4.4 (1.9; 10.6) | 6.7 (2.9; 16.0) | 9.4 (4.0; 22.5) | 12.6 (5.3; 30.0) |
| VES13 | 0.5 (0.2; 1.2) | 2.0 (0.9; 4.8) | 3.1 (1.2; 7.4) | 4.5 (1.9; 10.6) | 7.9 (3.3; 18.6) | 12.2 (4.8; 28.7) | 17.4 (7.4; 41.0) | 23.4 (9.9; 55.4) |
| WHRH | 0.5 (0.2; 1.3) | 1.8 (0.7; 4.2) | 2.5 (1.3; 6.1) | 3.5 (1.4; 8.3) | 5.6 (2.3; 13.4) | 8.1 (4.2; 19.4) | 11.0 (4.6; 26.4) | 14.3 (6.0; 34.2) |
| ZED1 | 0.4 (0.2; 0.8) | 1.1 (0.6; 2.0) | 1.4 (0.8; 2.7) | 1.9 (1.0; 3.5) | 2.8 (1.5; 5.2) | 3.8 (2.0; 7.1) | 4.9 (2.6; 9.2) | 6.0 (3.2; 11.4) |
| ZED2 | 0.3 (0.2; 0.7) | 0.9 (0.5; 1.8) | 1.3 (0.7; 2.6) | 1.7 (0.9; 3.4) | 2.6 (1.3; 5.2) | 3.7 (1.8; 7.3) | 4.8 (2.4; 9.6) | 6.1 (3.1; 12.1) |
| ZED3 | 0.2 (0.1; 0.5) | 0.8 (0.3; 1.7) | 1.1 (0.5; 2.5) | 1.5 (0.7; 3.4) | 2.5 (1.1; 5.6) | 3.7 (1.7; 8.2) | 5.1 (2.3; 11.3) | 6.6 (3.0; 14.7) |

^1^Hazard ratios calculated from age at baseline to age at the end of the interval.

BDE= Beaver Dam Eye Study Index. BFI= Brief Frailty Index. CGA= Comprehensive Geriatric Assessment. CGAST= Comprehensive Geriatric Assessment Screening Tests. CSBA= Conselice Study of Brain Aging Score. EFIP= Evaluative Frailty Index for Physical Activity. EFS= Edmonton Frail Scale. FI40= 40-item Frailty Index. FI70= 70-item Frailty Index. FIBLSA= Frailty Index Beijing Longitudinal Study of Ageing. FIND= Frail Non-Disabled Questionnaire. FS= Frail Scale. FSS= Frailty Staging System. G8= G-8 Geriatric Screening Tool. GFI= Groningen Frailty Indicator. HRCA= Hebrew Rehabilitation Center for Aged Vulnerability Index. HSF= Health Status Form. IFQ= Inter-Frail Questionnaire. MFS= Modified Frailty Score. MPHF= Modified Phenotype of Frailty. NLTCS= Long Term Care Survey Frailty Index. PFI= Physical Frailty Index. PHF= Phenotype of Frailty. SDF=, Static/Dynamic Frailty Index. SHCFS= Canadian Study of Health and Aging Clinical Frailty Scale·. SI= Screening Instrument. SOF= Study of Osteoporotic Fractures. SPPB= Short Physical Performance Battery. SPQ= Sherbrooke Postal Questionnaire. TFI= Tilburg Frailty Indicator. VES13= Vulnerable Elders Survey. WHRH= WHOAFC & self-reported health. ZED1= ZutPhen Elderly Study (Physical Activity & Low Energy). ZED2= ZutPhen Elderly Study (Physical Activity & Weight Loss). ZED3= ZutPhen Elderly Study (Physical Activity & Low BMI).
