# Supplementary material for: Comparative analysis of the association between 35 frailty scores and cardiovascular events, cancer, and total mortality in an elderly general population in England: An observational study
Source: PLoS Med. 2018 Mar 27;15(3):e1002543. doi: 10.1371/journal.pmed.1002543 (PMC5870943; doi:10.1371/journal.pmed.1002543)
Supplement: S6 Table — (DOCX) [file pmed.1002543.s007.docx]

**S6 Table.** Cardiovascular hazard ratios of frailty scores assessed in intervals from1 to 7 years1: age-adjusted model and categorical analysis

| Scores | HR1 (LCI; UCI) | HR2 (LCI; UCI) | HR2.5 (LCI; UCI) | HR3 (LCI; UCI) | HR4 (LCI; UCI) | HR5 (LCI; UCI) | HR6 (LCI; UCI) | HR7 (LCI; UCI) |
| --- | --- | --- | --- | --- | --- | --- | --- | --- |
| BFI frail | 0.3 (0.2; 0.7) | 0.7 (0.2; 2.4) | 0.9 (0.2; 3.7) | 1.1 (0.3; 5.1) | 1.6 (0.3; 8.7) | 2.0 (0.5; 13.1) | 2.5 (0.3; 18.4) | 3.0 (0.4; 24.4) |
| CGA frail | 0.6 (0.3; 1.0) | 1.0 (0.3; 3.0) | 1.7 (0.3; 4.4) | 2.0 (0.6; 6.0) | 2.9 (0.6; 9.8) | 3.7 (0.6; 14.2) | 4.6 (0.6; 19.3) | 5.5 (0.0; 24.9) |
| CGA pre-frail | 0.8 (0.6; 1.0) | 1.0 (0.6; 2.9) | 2.2 (0.6; 4.0) | 2.7 (0.8; 5.3) | 3.8 (0.8; 8.1) | 4.9 (0.5; 11.2) | 6.1 (0.8; 14.7) | 7.3 (0.9; 18.4) |
| CGAST frail | 0.8 (0.5; 1.3) | 1.3 (0.5; 4.0) | 2.1 (0.5; 5.7) | 2.5 (0.8; 7.6) | 3.4 (0.8; 11.9) | 4.3 (0.7; 17.0) | 5.2 (0.8; 22.6) | 6.2 (0.0; 28.9) |
| CGAST pre frail | 1.0 (0.7; 1.6) | 1.6 (0.7; 4.5) | 2.7 (0.7; 6.3) | 3.3 (1.0; 8.3) | 4.5 (1.0; 12.8) | 5.7 (1.8; 17.8) | 6.9 (1.0; 23.4) | 8.1 (1.5; 29.5) |
| CSBA frail | 0.7 (0.5; 1.0) | 1.0 (0.5; 2.1) | 1.3 (0.5; 2.6) | 1.5 (0.7; 3.2) | 1.8 (0.7; 4.3) | 2.2 (0.3; 5.5) | 2.5 (0.7; 6.7) | 2.7 (0.1; 7.9) |
| EFS frail | 1.2 (0.5; 2.5) | 2.5 (0.5; 5.9) | 1.8 (0.5; 7.8) | 1.9 (1.2; 9.7) | 2.2 (1.2; 13.9) | 2.4 (1.9; 18.2) | 2.6 (1.2; 22.8) | 2.8 (1.9; 27.6) |
| FI40 frail | 0.8 (0.5; 1.1) | 1.1 (0.5; 2.6) | 1.7 (0.5; 3.4) | 1.9 (0.8; 4.2) | 2.5 (0.8; 6.0) | 3.0 (0.4; 7.9) | 3.5 (0.8; 9.9) | 3.9 (0.6; 11.9) |
| FI70 frail | 0.8 (0.6; 1.2) | 1.2 (0.6; 2.7) | 1.7 (0.6; 3.5) | 1.9 (0.8; 4.3) | 2.4 (0.8; 6.0) | 2.9 (0.4; 7.8) | 3.3 (0.8; 9.6) | 3.7 (0.7; 11.5) |
| FiND frail | 0.6 (0.4; 1.0) | 1.0 (0.4; 2.5) | 1.4 (0.4; 3.4) | 1.6 (0.6; 4.4) | 2.0 (0.6; 6.5) | 2.5 (0.4; 8.8) | 2.9 (0.6; 11.3) | 3.3 (0.5; 14.0) |
| FS frail | 0.5 (0.3; 1.0) | 1.0 (0.3; 3.3) | 1.6 (0.3; 4.9) | 2.0 (0.5; 6.8) | 2.7 (0.5; 11.3) | 3.5 (0.6; 16.6) | 4.4 (0.5; 22.9) | 5.2 (0.3; 30.0) |
| FS pre- frail | 0.8 (0.6; 1.1) | 1.1 (0.6; 3.0) | 2.3 (0.6; 4.1) | 2.8 (0.8; 5.4) | 3.9 (0.8; 8.2) | 5.1 (0.5; 11.5) | 6.3 (0.8; 15.0) | 7.5 (0.0; 18.8) |
| FSS frail | 0.6 (0.3; 0.9) | 0.9 (0.3; 2.5) | 1.4 (0.3; 3.5) | 1.7 (0.6; 4.6) | 2.3 (0.6; 7.1) | 2.8 (0.4; 10.0) | 3.4 (0.6; 13.1) | 4.0 (0.5; 16.5) |
| FSS pre frail | 0.8 (0.6; 1.1) | 1.1 (0.6; 2.7) | 2.0 (0.6; 3.6) | 2.4 (0.8; 4.5) | 3.2 (0.8; 6.6) | 4.0 (0.4; 8.9) | 4.9 (0.8; 11.4) | 5.7 (0.7; 13.9) |
| G8 frail | 0.8 (0.5; 1.2) | 1.2 (0.5; 2.8) | 1.7 (0.5; 3.7) | 2.0 (0.8; 4.6) | 2.6 (0.8; 6.7) | 3.1 (0.4; 8.8) | 3.7 (0.8; 11.1) | 4.2 (0.8; 13.4) |
| GFI frail | 0.6 (0.4; 0.9) | 0.9 (0.4; 2.1) | 1.3 (0.4; 2.7) | 1.5 (0.6; 3.4) | 1.9 (0.6; 4.8) | 2.3 (0.3; 6.3) | 2.7 (0.6; 7.8) | 3.1 (0.1; 9.5) |
| HRCA frail | 0.8 (0.5; 1.1) | 1.1 (0.5; 2.5) | 1.6 (0.5; 3.3) | 1.8 (0.8; 4.1) | 2.3 (0.8; 5.7) | 2.8 (0.4; 7.5) | 3.2 (0.8; 9.2) | 3.6 (0.5; 11.1) |
| IFQ frail | 1.0 (0.4; 2.4) | 2.4 (0.4; 6.6) | 1.6 (0.4; 9.2) | 1.8 (1.0; 12.1) | 2.1 (1.0; 18.5) | 2.4 (1.12; 25.7) | 2.6 (1.0; 33.7) | 2.9 (1.6; 42.3) |
| MFS frail | 0.6 (0.3; 1.3) | 1.3 (0.3; 4.3) | 1.6 (0.3; 6.3) | 2.0 (0.6; 8.6) | 2.7 (0.6; 14.2) | 3.3 (0.8; 20.9) | 4.0 (0.6; 28.7) | 4.7 (0.3; 37.5) |
| MFS pre-frail | 0.8 (0.4; 1.5) | 1.5 (0.4; 4.8) | 2.1 (0.4; 7.0) | 2.5 (0.8; 9.4) | 3.3 (0.8; 15.2) | 4.2 (0.9; 22.1) | 5.1 (0.8; 29.9) | 5.9 (0.8; 38.6) |
| PFI frail | 0.7 (0.3; 1.4) | 1.4 (0.3; 4.8) | 1.8 (0.3; 7.1) | 2.2 (0.7; 9.9) | 2.9 (0.7; 16.4) | 3.7 (0.9; 24.4) | 4.5 (0.7; 33.7) | 5.3 (0.8; 44.2) |
| PFI pre frail | 1.0 (0.7; 1.4) | 1.4 (0.7; 3.7) | 2.5 (0.7; 5.1) | 3.1 (1.0; 6.5) | 4.2 (1.0; 9.8) | 5.3 (1.6; 13.4) | 6.4 (1.0; 17.3) | 7.5 (1.7; 21.5) |
| PHF frail | 0.6 (0.3; 1.2) | 1.2 (0.3; 4.3) | 1.9 (0.3; 6.6) | 2.4 (0.6; 9.2) | 3.3 (0.6; 15.7) | 4.4 (0.9; 23.8) | 5.5 (0.6; 33.3) | 6.6 (0.3; 44.3) |
| PHF pre-frail | 0.8 (0.4; 1.3) | 1.3 (0.4; 4.2) | 2.3 (0.4; 6.2) | 2.8 (0.8; 8.5) | 4.0 (0.8; 13.9) | 5.3 (0.8; 20.5) | 6.6 (0.8; 28.0) | 7.9 (0.2; 36.6) |
| SDFI frail | 0.6 (0.4; 0.9) | 0.9 (0.4; 2.2) | 1.4 (0.4; 2.9) | 1.6 (0.6; 3.6) | 2.1 (0.6; 5.1) | 2.5 (0.3; 6.6) | 2.9 (0.6; 8.3) | 3.3 (0.2; 10.0) |
| SHCFS frail | 0.7 (0.4; 1.1) | 1.1 (0.4; 2.8) | 1.4 (0.4; 3.7) | 1.6 (0.7; 4.7) | 2.0 (0.7; 6.9) | 2.4 (0.4; 9.2) | 2.8 (0.7; 11.7) | 3.1 (0.8; 14.4) |
| SI frail | 0.5 (0.2; 1.3) | 1.3 (0.2; 4.1) | 1.2 (0.2; 5.9) | 1.3 (0.5; 8.0) | 1.7 (0.5; 13.0) | 2.0 (0.8; 18.8) | 2.4 (0.5; 25.5) | 2.7 (0.1; 33.0) |
| SOF frail | 0.5 (0.3; 0.9) | 0.9 (0.3; 3.1) | 1.4 (0.3; 4.5) | 1.8 (0.5; 6.1) | 2.4 (0.5; 9.9) | 3.1 (0.6; 14.5) | 3.8 (0.5; 19.7) | 4.5 (0.1; 25.6) |
| SOF pre-frail | 0.7 (0.5; 1.0) | 1.0 (0.5; 2.8) | 2.1 (0.5; 3.8) | 2.5 (0.7; 5.0) | 3.4 (0.7; 7.5) | 4.4 (0.5; 10.3) | 5.4 (0.7; 13.4) | 6.4 (0.8; 16.7) |
| SPPB frail | 0.6 (0.4; 0.9) | 0.9 (0.4; 2.1) | 1.3 (0.4; 2.7) | 1.5 (0.6; 3.3) | 1.9 (0.6; 4.6) | 2.2 (0.3; 6.0) | 2.6 (0.6; 7.4) | 2.9 (0.1; 8.8) |
| SPQ frail | 0.5 (0.4; 0.6) | 0.6 (0.4; 0.9) | 0.6 (0.4; 1.0) | 0.6 (0.5; 1.2) | 0.7 (0.5; 1.4) | 0.7 (0.1; 1.5) | 0.8 (0.5; 1.7) | 0.8 (0.9; 1.9) |
| TFI frail | 0.7 (0.5; 1.0) | 1.0 (0.5; 2.1) | 1.3 (0.5; 2.6) | 1.5 (0.7; 3.2) | 1.9 (0.7; 4.4) | 2.2 (0.3; 5.6) | 2.5 (0.7; 6.8) | 2.8 (0.1; 8.1) |
| VES13 frail | 0.7 (0.5; 1.0) | 1.0 (0.5; 2.4) | 1.5 (0.5; 3.1) | 1.7 (0.7; 3.9) | 2.2 (0.7; 5.5) | 2.7 (0.3; 7.2) | 3.1 (0.7; 9.0) | 3.5 (0.4; 10.8) |
| WHRH frail | 0.8 (0.5; 1.2) | 1.2 (0.5; 2.6) | 1.5 (0.5; 3.3) | 1.7 (0.8; 4.1) | 2.0 (0.8; 5.7) | 2.4 (0.4; 7.3) | 2.7 (0.8; 8.9) | 3.0 (0.6; 10.6) |
| ZED1 frail | 0.8 (0.4; 1.8) | 1.8 (0.4; 4.1) | 1.2 (0.4; 5.4) | 1.3 (0.8; 6.8) | 1.4 (0.8; 9.6) | 1.6 (0.6; 12.6) | 1.7 (0.8; 15.7) | 1.8 (0.1; 19.0) |
| ZED2 frail | 0.7 (0.2; 1.9) | 1.9 (0.2; 7.4) | 1.5 (0.2; 11.3) | 1.8 (0.7; 16.0) | 2.3 (0.7; 27.8) | 2.8 (0.16; 42.6) | 3.3 (0.7; 60.5) | 3.8 (0.4; 81.3) |
| ZED3 frail | 0.1 (0.0; 3.4) | 3.4 (0.0; >99.9) | 0.6 (0.0; >99.9) | 0.9 (0.1; >99.9) | 1.5 (0.1; >99.9) | 2.2 (1.0; >99.9) | 3.0 (0.1; >99.9) | 4.0 (0.1; >99.9) |

^1^Hazard ratios calculated from age at baseline to age at the end of the interval.

BFI= Brief Frailty Index. CGA= Comprehensive Geriatric Assessment. CGAST= Comprehensive Geriatric Assessment Screening Tests. CSBA= Conselice Study of Brain Aging Score. EFS= Edmonton Frail Scale. FI40= 40-item Frailty Index. FI70= 70-item Frailty Index. FIND= Frail Non-Disabled Questionnaire. FS= Frail Scale. FSS= Frailty Staging System. G8= G-8 Geriatric Screening Tool. GFI= Groningen Frailty Indicator. HRCA= Hebrew Rehabilitation Center for Aged Vulnerability Index. IFQ= Inter-Frail Questionnaire. MFS= Modified Frailty Score. PFI= Physical Frailty Index. PHF= Phenotype of Frailty. SDFI=, Static/Dynamic Frailty Index. SHCFS= Canadian Study of Health and Aging Clinical Frailty Scale·. SI= Screening Instrument. SOF= Study of Osteoporotic Fractures. SPPB= Short Physical Performance Battery. SPQ= Sherbrooke Postal Questionnaire. TFI= Tilburg Frailty Indicator. VES13= Vulnerable Elders Survey. WHRH= WHOAFC & self-reported health. ZED1= ZutPhen Elderly Study (Physical Activity & Low Energy). ZED2= ZutPhen Elderly Study (Physical Activity & Weight Loss). ZED3= ZutPhen Elderly Study (Physical Activity & Low BMI).
