# Supplementary material for: Comparative analysis of the association between 35 frailty scores and cardiovascular events, cancer, and total mortality in an elderly general population in England: An observational study
Source: PLoS Med. 2018 Mar 27;15(3):e1002543. doi: 10.1371/journal.pmed.1002543 (PMC5870943; doi:10.1371/journal.pmed.1002543)
Supplement: S7 Table — (DOCX) [file pmed.1002543.s008.docx]

**S7 Table.** Cancer hazard ratios of frailty scores (n=4792) calculated at median time follow-up (2.5 years)

| **Continuous analysis** | | | | | **Cut-off analysis** | | | | |
| --- | --- | --- | --- | --- | --- | --- | --- | --- | --- |
|  | **HR (95% CI)** | **HR (95% CI)** | **HR (95% CI)** | **HR (95% CI)** |  | **HR (95% CI)** | **HR (95% CI)** | **HR (95% CI)** | **HR (95% CI)** |
| **Frailty Score** | **Model 0^1^** | **Model 1^2^** | **Model 2^3^** | **Model 3^4^** | **Frailty Score** | **Model 0^1^** | **Model 1^2^** | **Model 2^3^** | **Model 3^4^** |
| **Phenotype of frailty approach** | | | | | | | | | |
| SPPB | 1.8 (0.8; 3.9) | 1.8 (1.0; 3.3) | 1.5 (0.8; 2.8) | 1.6 (0.8; 3.2) | SOF frail | 1.4 (0.6; 3.2) | 1.5 (0.6; 3.4) | 1.4 (0.1; 3.3) | 1.4 (0.5; 3.5) |
| SOF | 1.5 (0.9; 2.7) | 1.6 (0.9; 2.9) | 1.5 (0.9; 2.8) | 1.5 (0.8; 3.0) | SOF pre-frail | 1.3 (0.8; 2.2) | 1.4 (0.8; 2.3) | 1.3 (0.1; 2.2) | 1.4 (0.8; 2.3) |
| PHF | 1.7 (1.0; 2.9) | 1.6 (1.0; 2.6) | 1.4 (1.0; 2.4) | 1.4 (0.8; 2.5) | PFI frail | 1.2 (0.4; 4.2) | 1.3 (0.4; 4.5) | 1.2 (0.1; 4.4) | 1.2 (0.3; 4.2) |
| ZED2 | 1.5 (0.9; 2.3) | 1.5 (1.0; 2.4) | 1.4 (0.9; 2.3) | 1.4 (0.9; 2.3) | PFI pre frail | 1.3 (0.7; 2.5) | 1.4 (0.8; 2.6) | 1.3 (0.1; 2.5) | 1.3 (0.7; 2.5) |
| MPHF | 1.3 (0.8; 2.2) | 1.5 (0.9; 2.5) | 1.3 (0.8; 2.2) | 1.1 (0.6; 2.0) | FS frail | 1.2 (0.5; 2.7) | 1.2 (0.5; 2.8) | 1.2 (0.1; 2.7) | 1.0 (0.4; 2.5) |
| FS | 1.3 (0.7; 2.3) | 1.4 (0.7; 2.6) | 1.2 (0.7; 2.3) | 0.9 (0.5; 1.9) | FS pre- frail | 1.3 (0.8; 2.0) | 1.3 (0.8; 2.1) | 1.3 (0.1; 2.0) | 1.2 (0.7; 2.0) |
| FiND | 1.3 (0.8; 2.2) | 1.4 (0.8; 2.3) | 1.2 (0.8; 2.1) | 1.1 (0.6; 2.2) | PHF frail | 1.2 (0.5; 2.8) | 1.3 (0.5; 3.0) | 1.2 (0.0; 2.8) | 1.1 (0.4; 3.9) |
| PFI | 1.2 (0.7; 2.0) | 1.3 (0.8; 2.2) | 1.2 (0.7; 2.1) | 1.1 (0.6; 2.0) | PHF pre-frail | 1.2 (0.6; 2.3) | 1.2 (0.6; 2.4) | 1.2 (0.0; 2.3) | 1.2 (0.6; 2.4) |
| ZED1 | 1.1 (0.7; 1.7) | 1.2 (0.8; 1.9) | 1.1 (0.7; 1.8) | 1.0 (0.5; 1.7) | FiND frail | 1.2 (0.6; 2.4) | 1.3 (0.7; 2.5) | 1.2 (0.0; 2.4) | 1.2 (0.6; 2.4) |
| ZED3 | 1.0 (0.6; 1.7) | 1.1 (0.6; 2.0) | 1.0 (0.6; 1.8) | 0.9 (0.5; 1.7) | SPPB frail | 1.1 (0.7; 1.8) | 1.2 (0.7; 1.9) | 1.1 (0.1; 1.8) | 1.1 (0.7; 1.9) |
| BDE | 0.7 (0.4; 1.2) | 0.7 (0.4; 1.2) | 0.6 (0.4; 1.1) | 0.9 (0.6; 1.6) | ZED1 frail | 1.1 (0.1; 8.2) | 1.1 (0.1; 8.4) | 1.1 (0.1; 8.2) | 1.0 (0.1; 8.7) |
|  |  |  |  |  | ZED2 frail | 0.5 (0.2; 1.1) | 0.4 (0.0; 42.6) | 0.5 (0.2; 1.1) | 0.4 (0.0; 42.3) |
|  |  |  |  |  | ZED3 frail | 0.6 (0.0; >99.9) | 0 (0; >99.9) | 0 (0; >99.9) | 0 (0; >99.9) |
| **Multidimensional approach** | | | | | | | | | |
| EFS | 2.0 (0.8; 4.8) | 2.4 (1.0; 5.7) | 2.0 (0.8; 4.9) | 1.4 (0.5; 4.2) | SPQ frail | 1.1 (0.7; 1.9) | 1.4 (1.0; 2.1) | 1.1 (0.9; 1.4) | 1.1 (0.6; 2.9) |
| G8 | 1.9 (0.8; 4.3) | 2.0 (0.9; 4.5) | 1.2 (0.8; 2.1) | 1.9 (0.7; 5.0) | IFQ frail | 1.3 (0.4; 4.5) | 1.4 (0.4; 4.9) | 1.4 (0.8; 2.5) | 1.2 (0.3; 4.5) |
| CGAST | 1.5 (0.7; 3.2) | 1.8 (0.9; 3.7) | 1.6 (0.7; 3.3) | 1.4 (0.6; 3.4) | EFS frail | 1.3 (0.4; 4.4) | 1.4 (0.4; 4.7) | 1.3 (0.4; 4.4) | 1.2 (0.3; 4.1) |
| GFI | 1.5 (0.7; 3.2) | 1.8 (0.8; 3.7) | 1.5 (0.7; 3.1) | 1.2 (0.5; 3.2) | FSS frail | 1.2 (0.6; 2.3) | 1.2 (0.6; 2.4) | 1.2 (0.2; 2.3) | 1.1 (0.5; 2.4) |
| IFQ | 1.3 (0.6; 2.8) | 1.6 (0.7; 3.3) | 1.3 (0.6; 2.9) | 1.1 (0.5; 2.6) | FSS pre frail | 1.3 (0.8; 2.1) | 1.3 (0.8; 2.1) | 1.3 (0.2; 2.1) | 1.3 (0.8; 2.2) |
| TFI | 1.2 (0.6; 2.4) | 1.5 (0.8; 2.9) | 1.2 (0.6; 2.5) | 1.0 (0.4; 2.3) | CGAST frail | 1.2 (0.6; 2.2) | 1.2 (0.7; 2.3) | 1.2 (0.0; 2.2) | 1.1 (0.5; 2.4) |
| CSBA | 2.4 (1.1; 5.2) | 1.4 (0.6; 3.3) | 1.1 (1.1; 2.7) | 0.7 (0.2; 2.1) | CGAST pre frail | 1.1 (0.6; 2.0) | 1.1 (0.6; 2.0) | 1.1 (0.0; 2.0) | 1.1 (0.6; 2.0) |
| SPQ | 1.2 (0.6; 2.4) | 1.4 (0.7; 2.9) | 1.3 (0.6; 2.6) | 1.1 (0.5; 2.3) | G8 frail | 1.1 (0.7; 1.7) | 1.1 (0.7; 1.8) | 1.1 (0.1; 1.8) | 1.1 (0.6; 1.9) |
| FSS | 1.3 (0.7; 2.2) | 1.4 (0.8; 2.3) | 1.2 (0.7; 2.1) | 1.1 (0.6; 2.1) | GFI frail | 1.1 (0.7; 1.8) | 1.1 (0.7; 1.9) | 1.1 (0.5; 1.8) | 1.0 (0.6; 1.8) |
| SDFI | 0.9 (0.5; 1.8) | 1.3 (0.7; 2.7) | 1.1 (0.5; 2.3) | 0.8 (0.4; 2.0) | MFS frail | 0.8 (0.3; 1.9) | 1.1 (0.5; 2.4) | 0.8 (0.0; 2.3) | 0.9 (0.4; 2.2) |
| SI | 1.0 (0.5; 2.1) | 1.2 (0.6; 2.7) | 1.1 (0.5; 2.4) | 0.9 (0.4; 2.1) | MFS pre-frail | 0.9 (0.4; 1.9) | 1.1 (0.5; 2.3) | 0.9 (0.0; 2.2) | 1.0 (0.5; 2.2) |
| MFS | 1.0 (0.6; 2.1) | 1.2 (0.7; 2.1) | 1.1 (0.6; 1.9) | 0.9 (0.5; 1.6) | CSBA frail | 1.2 (0.8; 2.0) | 1.1 (0.7; 1.8) | 1.0 (0.6; 1.7) | 1.0 (0.6; 1.7) |
| HSF | 1.1 (0.5; 2.1) | 1.1 (0.6; 2.3) | 1.0 (0.5; 2.0) | 0.7 (0.3; 1.6) | TFI frail | 1.0 (0.6; 1.6) | 1.1 (0.7; 1.7) | 1.0 (0.8; 1.2) | 0.9 (0.5; 1.6) |
| BFI | 0.8 (0.5; 1.4) | 0.9 (0.5; 1.6) | 0.8 (0.5; 1.4) | 0.7 (0.4; 1.3) | SI frail | 0.9 (0.1; 5.6) | 1.0 (0.2; 6.0) | 0.9 (0.2; 5.7) | 0.9 (0.1; 6.4) |
|  |  |  |  |  | SDFI frail | 0.9 (0.5; 1.5) | 1.0 (0.6; 1.6) | 0.9 (0.0; 1.5) | 0.8 (0.5; 1.5) |
|  |  |  |  |  | BFI frail | 0.7 (0.3; 2.0) | 0.8 (0.3; 2.1) | 0.7 (0.2; 2.0) | 0.7 (0.3; 2.0) |
| **Accumulation of deficits approach** | | | | | | | | | |
| FI70 | 1.2 (0.5; 2.7) | 1.5 (0.7; 3.6) | 1.3 (0.5; 3.0) | 0.7 (0.2; 2.2) | CGA frail | 1.0 (0.5; 2.0) | 1.1 (0.6; 2.2) | 1.0 (0.2; 2.1) | 0.9 (0.4; 2.0) |
| NLTCS | 1.4 (0.4; 4.4) | 1.5 (0.5; 4.9) | 1.3 (0.4; 4.1) | 0.7 (0.2; 2.7) | CGA pre-frail | 1.1 (0.7; 1.8) | 1.2 (0.8; 1.9) | 1.1 (0.2; 1.8) | 1.1 (0.7; 1.8) |
| FI40 | 1.2 (0.5; 2.8) | 1.5 (0.6; 3.6) | 1.2 (0.5; 3.0) | 0.7 (0.3; 2.0) | FI70 frail | 1.1 (0.6; 1.8) | 1.1 (0.7; 1.9) | 1.1 (0.2; 1.9) | 1.0 (0.5; 1.2) |
| EFIP | 1.2 (0.5; 2.8) | 1.4 (0.6; 3.3) | 1.2 (0.5; 2.8) | 0.7 (0.2; 2.1) | FI40 frail | 1.1 (0.6; 1.7) | 1.1 (0.7; 1.9) | 1.1 (0.2; 1.8) | 0.9 (0.5; 1.6) |
| FIBLSA | 1.2 (0.4; 3.1) | 1.4 (0.5; 3.7) | 1.3 (0.4; 3.0) | 0.6 (0.2; 1.9) |  |  |  |  |  |
| CGA | 1.0 (0.4; 2.9) | 1.4 (0.5; 3.9) | 1.1 (0.4; 3.2) | 0.6 (0.2; 2.0) |  |  |  |  |  |
| **Disability approach** | | | | | | | | | |
| WHRH | 1.3 (0.7; 2.4) | 1.5 (0.8; 2.7) | 1.3 (0.7; 2.5) | 1.4 (0.6; 2.9) | WHRH frail | 1.1 (0.6; 2.0) | 1.2 (0.6; 2.2) | 1.1 (0.6; 2.1) | 1.0 (0.5; 2.1) |
| VES13 | 1.2 (0.6; 2.3) | 1.4 (0.7; 2.8) | 1.3 (0.6; 2.5) | 1.3 (0.6; 2.8) | VES13 frail | 1.1 (0.6; 1.8) | 1.1 (0.7; 1.9) | 1.1 (0.1; 1.9) | 1.1 (0.6; 1.0) |
| HRCA | 1.2 (0.6; 2.4) | 1.4 (0.7; 2.9) | 1.2 (0.6; 2.5) | 1.1 (0.4; 2.7) | SHCFS frail | 1.1 (0.5; 2.2) | 1.1 (0.5; 2.3) | 1.0 (0.5; 2.2) | 1.0 (0.4; 2.1) |
| SHCFS | 1.2 (0.7; 2.0) | 1.2 (0.7; 2.1) | 1.1 (0.7; 1.9) | 0.9 (0.5; 1.8) | HRCA frail | 1.0 (0.6; 1.7) | 1.1 (0.6; 1.8) | 1.0 (0.2; 1.8) | 1.0 (0.5; 1.9) |

^1^Model 0= Crude models. ^2^Model 1= HR adjusted by sex. ^3^Model 2= Model 1 + smoking status and alcohol consumption. ^4^Model 3= Model 2 + physical activity, BMI, diabetes, hypertension, cardiovascular, anemia, COPD, arthritis, neuropsychiatric, depression, cognition, self-rated health & quality of life. Models were fitted using age as time scale, with time 0 = age at entry of study and time 1 =age at event or censoring date.

Abbreviations frailty scores: BDE= Beaver Dam Eye Study Index. BFI= Brief Frailty Index. CGA= Comprehensive Geriatric Assessment. CGAST= Comprehensive Geriatric Assessment Screening Tests. CSBA= Conselice Study of Brain Aging Score. EFIP= Evaluative Frailty Index for Physical Activity. EFS= Edmonton Frail Scale. FI40= 40-item Frailty Index. FI70= 70-item Frailty Index (SHARE). FIBLSA= Frailty Index Beijing Longitudinal Study of Ageing. FiND= Frail Non-Disabled Questionnaire. FS= Frail Scale. FSS= Frailty Staging System. G8= G-8 Geriatric Screening Tool. GFI= Groningen Frailty Indicator. HRCA= Hebrew Rehabilitation Center for Aged Vulnerability Index. HSF= Health Status Form. IFQ= Inter-Frail Questionnaire. MFS= Modified Frailty Score. MPHF= Modified Phenotype of Frailty. NLTCS= Long Term Care Survey Frailty Index. PFI= Physical Frailty Index. PHF= Phenotype of Frailty. SDFI=, Static/Dynamic Frailty Index. SHCFS= Canadian Study of Health and Aging Clinical Frailty Scale·. SI= Screening Instrument. SOF= Study of Osteoporotic Fractures. SPPB= Short Physical Performance Battery. SPQ= Sherbrooke Postal Questionnaire. TFI= Tilburg Frailty Indicator. VES13= Vulnerable Elders Survey. WHRH= WHOAFC & self-reported health. ZED1= ZutPhen Elderly Study (Physical Activity & Low Energy). ZED2= ZutPhen Elderly Study (Physical Activity & Weight Loss). ZED3= ZutPhen Elderly Study (Physical Activity & Low BMI).
