# Supplementary material for: Comparative analysis of the association between 35 frailty scores and cardiovascular events, cancer, and total mortality in an elderly general population in England: An observational study
Source: PLoS Med. 2018 Mar 27;15(3):e1002543. doi: 10.1371/journal.pmed.1002543 (PMC5870943; doi:10.1371/journal.pmed.1002543)
Supplement: S8 Table — (DOCX) [file pmed.1002543.s009.docx]

**S8 Table.** Cancer hazard ratios of frailty scores assessed in intervals from1 to 7 years1: age-adjusted model and continuous analysis

| Scores | HR 1 (LCI; UCI) | HR 2 (LCI; UCI) | HR 2.5 (LCI; UCI) | HR 3 (LCI; UCI) | HR 4 (LCI; UCI) | HR 5 (LCI; UCI) | HR 6 (LCI; UCI) | HR 7 (LCI; UCI) |
| --- | --- | --- | --- | --- | --- | --- | --- | --- |
| BDE | 0.6 (0.3; 1.0) | 0.7 (0.4; 1.2) | 0.7 (0.4; 1.2) | 0.7 (0.4; 1.3) | 0.7 (0.4; 1.1) | 0.8 (0.5; 1.4) | 0.9 (0.5; 1.5) | 0.9 (0.5; 1.5) |
| BFI | 0.5 (0.3; 0.9) | 0.7 (0.4; 1.2) | 0.8 (0.5; 1.4) | 0.8 (0.5; 1.5) | 0.7 (0.4; 1.2) | 1.1 (0.6; 1.8) | 1.1 (0.7; 2.0) | 1.2 (0.7; 2.1) |
| CGA | 0.4 (0.2; 1.3) | 0.8 (0.3; 2.3) | 1.0 (0.4; 2.9) | 1.2 (0.4; 3.4) | 1.0 (0.4; 3.0) | 1.9 (0.7; 5.3) | 2.2 (0.8; 6.2) | 2.5 (0.9; 7.1) |
| CGAST | 0.9 (0.4; 1.8) | 1.3 (0.7; 2.8) | 1.5 (0.7; 3.2) | 1.7 (0.8; 3.6) | 1.5 (0.7; 3.0) | 2.4 (1.2; 4.9) | 2.7 (0.3; 5.5) | 2.9 (1.4; 6.1) |
| CSBA | 1.5 (0.7; 3.3) | 2.1 (1.0; 4.6) | 2.4 (1.1; 5.2) | 2.6 (1.2; 5.7) | 2.2 (1.0; 4.8) | 3.3 (1.5; 7.3) | 3.7 (1.7; 8.0) | 3.9 (1.8; 8.6) |
| EFIP | 0.6 (0.3; 1.4) | 1.0 (0.4; 2.3) | 1.2 (0.5; 2.8) | 1.4 (0.6; 3.2) | 1.2 (0.5; 2.7) | 2.0 (0.9; 4.7) | 2.3 (0.0; 5.4) | 2.6 (1.1; 6.1) |
| EFS | 0.8 (0.3; 2.0) | 1.6 (0.7; 3.9) | 2.0 (0.8; 4.8) | 2.4 (1.0; 5.7) | 2.1 (0.9; 5.1) | 3.8 (1.6; 9.3) | 4.5 (0.9; 11.1) | 5.3 (2.2; 12.8) |
| FI40 | 0.6 (0.2; 1.3) | 1.0 (0.4; 2.4) | 1.2 (0.5; 2.8) | 1.4 (0.6; 3.3) | 1.2 (0.5; 2.9) | 2.1 (0.9; 5.0) | 2.4 (0.0; 5.9) | 2.8 (1.1; 6.7) |
| FI70 | 0.6 (0.2; 1.3) | 1.0 (0.4; 2.3) | 1.2 (0.5; 2.7) | 1.3 (0.6; 3.1) | 1.2 (0.5; 2.7) | 2.0 (0.9; 4.6) | 2.3 (0.0; 5.3) | 2.6 (1.1; 6.0) |
| FIBLSA | 0.5 (0.2; 1.3) | 1.0 (0.4; 2.5) | 1.2 (0.4; 3.1) | 1.4 (0.5; 3.7) | 1.3 (0.5; 3.3) | 2.3 (0.9; 6.0) | 2.7 (0.0; 7.1) | 3.1 (1.2; 8.2) |
| FiND | 0.9 (0.5; 1.5) | 1.2 (0.7; 2.0) | 1.3 (0.8; 2.2) | 1.4 (0.8; 2.4) | 1.2 (0.7; 2.0) | 1.7 (1.0; 3.0) | 1.9 (0.1; 3.2) | 2.0 (1.2; 3.4) |
| FS | 0.8 (0.5; 1.6) | 1.1 (0.6; 2.1) | 1.3 (0.7; 2.3) | 1.4 (0.7; 2.5) | 1.2 (0.6; 2.1) | 1.7 (0.9; 3.1) | 1.8 (0.0; 3.4) | 2.0 (1.1; 3.6) |
| FSS | 0.8 (0.5; 1.4) | 1.1 (0.7; 2.0) | 1.3 (0.7; 2.2) | 1.4 (0.8; 2.3) | 1.2 (0.7; 2.0) | 1.7 (1.0; 2.9) | 1.8 (0.1; 3.1) | 2.0 (1.2; 3.4) |
| G8 | 1.3 (0.6; 3.1) | 1.7 (0.7; 4.0) | 1.9 (0.8; 4.3) | 2.0 (0.9; 4.6) | 1.7 (0.7; 3.9) | 2.4 (1.0; 5.5) | 2.5 (1.1; 5.8) | 2.7 (1.2; 6.2) |
| GFI | 0.9 (0.4; 1.9) | 1.3 (0.6; 2.8) | 1.5 (0.7; 3.2) | 1.7 (0.8; 3.5) | 1.4 (0.7; 3.0) | 2.3 (1.1; 4.8) | 2.6 (0.2; 5.3) | 2.8 (1.3; 5.8) |
| HRCA | 0.6 (0.3; 1.3) | 1.0 (0.5; 2.1) | 1.2 (0.6; 2.4) | 1.3 (0.6; 2.8) | 1.1 (0.5; 2.4) | 1.9 (0.9; 3.9) | 2.2 (0.0; 4.5) | 2.4 (1.2; 5.0) |
| HSF | 0.6 (0.3; 1.3) | 0.9 (0.5; 1.9) | 1.1 (0.5; 2.1) | 1.2 (0.6; 2.4) | 1.0 (0.5; 2.0) | 1.6 (0.8; 3.1) | 1.7 (0.9; 3.5) | 1.9 (0.9; 3.8) |
| IFQ | 0.7 (0.3; 1.5) | 1.1 (0.5; 2.4) | 1.3 (0.6; 2.8) | 1.5 (0.7; 3.1) | 1.3 (0.6; 2.7) | 2.1 (1.0; 4.4) | 2.4 (0.1; 5.0) | 2.6 (1.3; 5.5) |
| MFS | 0.9 (0.5; 1.8) | 1.1 (0.6; 2.0) | 1.1 (0.6; 2.1) | 1.2 (0.6; 2.1) | 1.1 (0.6; 1.9) | 1.3 (0.7; 2.4) | 1.3 (0.7; 2.4) | 1.3 (0.7; 2.5) |
| MPHF | 0.9 (0.5; 1.5) | 1.2 (0.7; 2.0) | 1.3 (0.8; 2.2) | 1.4 (0.9; 2.4) | 1.2 (0.7; 2.0) | 1.8 (1.1; 3.0) | 1.9 (0.2; 3.2) | 2.1 (1.2; 3.5) |
| NLTCS | 0.5 (0.1; 1.5) | 1.0 (0.3; 3.4) | 1.4 (0.4; 4.4) | 1.7 (0.5; 5.4) | 1.6 (0.5; 5.1) | 3.0 (0.9; 9.7) | 3.7 (0.1; 12.0) | 4.4 (1.4; 14.3) |
| PFI | 0.7 (0.4; 1.3) | 1.1 (0.6; 1.8) | 1.2 (0.7; 2.0) | 1.3 (0.8; 2.2) | 1.1 (0.7; 1.9) | 1.7 (1.0; 2.9) | 1.9 (0.1; 3.2) | 2.1 (1.2; 3.5) |
| PHF | 1.3 (0.7; 2.2) | 1.6 (0.9; 2.7) | 1.7 (1.0; 2.9) | 1.8 (1.0; 3.0) | 1.5 (0.9; 2.6) | 2.1 (1.2; 3.5) | 2.2 (1.3; 3.7) | 2.3 (1.3; 3.9) |
| SDFI | 0.6 (0.3; 1.2) | 0.8 (0.4; 1.6) | 0.9 (0.5; 1.8) | 1.0 (0.5; 2.0) | 0.9 (0.4; 1.7) | 1.3 (0.7; 2.6) | 1.4 (0.7; 2.9) | 1.5 (0.8; 3.1) |
| SHCFS | 0.8 (0.5; 1.4) | 1.1 (0.6; 1.8) | 1.2 (0.7; 2.0) | 1.2 (0.7; 2.1) | 1.1 (0.6; 1.8) | 1.5 (0.9; 2.6) | 1.6 (0.0; 2.8) | 1.7 (1.0; 2.9) |
| SI | 0.4 (0.2; 1.0) | 0.8 (0.4; 1.8) | 1.0 (0.5; 2.1) | 1.2 (0.5; 2.5) | 1.0 (0.5; 2.2) | 1.8 (0.8; 3.9) | 2.1 (0.0; 4.6) | 2.4 (1.1; 5.2) |
| SOF | 0.9 (0.5; 1.7) | 1.4 (0.8; 2.4) | 1.5 (0.9; 2.7) | 1.7 (0.9; 3.0) | 1.4 (0.8; 2.5) | 2.2 (1.2; 3.9) | 2.4 (0.4; 4.3) | 2.6 (1.5; 4.7) |
| SPPB | 1.3 (0.6; 2.8) | 1.6 (0.7; 3.6) | 1.8 (0.8; 3.9) | 1.9 (0.8; 4.1) | 1.6 (0.7; 3.6) | 2.2 (1.0; 5.0) | 2.4 (1.1; 5.3) | 2.5 (1.1; 5.6) |
| SPQ | 0.7 (0.3; 1.4) | 1.0 (0.5; 2.1) | 1.2 (0.6; 2.4) | 1.3 (0.7; 2.7) | 1.1 (0.6; 2.3) | 1.8 (0.9; 3.8) | 2.1 (0.0; 4.2) | 2.3 (1.1; 4.7) |
| TFI | 0.8 (0.4; 1.5) | 1.1 (0.6; 2.1) | 1.2 (0.6; 2.4) | 1.4 (0.7; 2.6) | 1.1 (0.6; 2.2) | 1.7 (0.9; 3.3) | 1.9 (0.0; 3.7) | 2.1 (1.1; 4.0) |
| VES13 | 0.7 (0.4; 1.3) | 1.1 (0.5; 2.0) | 1.2 (0.6; 2.3) | 1.3 (0.7; 2.6) | 1.1 (0.6; 2.2) | 1.8 (0.9; 3.6) | 2.0 (0.1; 4.0) | 2.2 (1.2; 4.4) |
| WHRH | 0.8 (0.4; 1.5) | 1.1 (0.6; 2.1) | 1.3 (0.7; 2.4) | 1.4 (0.7; 2.6) | 1.2 (0.6; 2.2) | 1.8 (1.0; 3.5) | 2.0 (0.1; 3.8) | 2.2 (1.2; 4.1) |
| ZED1 | 0.7 (0.4; 1.1) | 1.0 (0.6; 1.5) | 1.1 (0.7; 1.7) | 1.2 (0.8; 1.9) | 1.0 (0.6; 1.6) | 1.6 (1.0; 2.5) | 1.7 (0.1; 2.8) | 1.9 (1.2; 3.0) |
| ZED2 | 1.1 (0.7; 1.7) | 1.4 (0.9; 2.1) | 1.5 (0.9; 2.3) | 1.5 (1.0; 2.5) | 1.3 (0.8; 2.1) | 1.8 (1.1; 2.9) | 1.9 (1.2; 3.1) | 2.0 (1.3; 3.2) |
| ZED3 | 0.6 (0.4; 1.1) | 0.9 (0.5; 1.6) | 1.0 (0.6; 1.7) | 1.1 (0.6; 1.9) | 0.9 (0.5; 1.6) | 1.4 (0.8; 2.4) | 1.5 (0.8; 2.7) | 1.6 (0.9; 2.9) |

^1^Hazard ratios calculated from age at baseline to age at the end of the interval.

BDE= Beaver Dam Eye Study Index. BFI= Brief Frailty Index. CGA= Comprehensive Geriatric Assessment. CGAST= Comprehensive Geriatric Assessment Screening Tests. CSBA= Conselice Study of Brain Aging Score. EFIP= Evaluative Frailty Index for Physical Activity. EFS= Edmonton Frail Scale. FI40= 40-item Frailty Index. FI70= 70-item Frailty Index. FIBLSA= Frailty Index Beijing Longitudinal Study of Ageing. FIND= Frail Non-Disabled Questionnaire. FS= Frail Scale. FSS= Frailty Staging System. G8= G-8 Geriatric Screening Tool. GFI= Groningen Frailty Indicator. HRCA= Hebrew Rehabilitation Center for Aged Vulnerability Index. HSF= Health Status Form. IFQ= Inter-Frail Questionnaire. MFS= Modified Frailty Score. MPHF= Modified Phenotype of Frailty. NLTCS= Long Term Care Survey Frailty Index. PFI= Physical Frailty Index. PHF= Phenotype of Frailty. SDF=, Static/Dynamic Frailty Index. SHCFS= Canadian Study of Health and Aging Clinical Frailty Scale·. SI= Screening Instrument. SOF= Study of Osteoporotic Fractures. SPPB= Short Physical Performance Battery. SPQ= Sherbrooke Postal Questionnaire. TFI= Tilburg Frailty Indicator. VES13= Vulnerable Elders Survey. WHRH= WHOAFC & self-reported health. ZED1= ZutPhen Elderly Study (Physical Activity & Low Energy). ZED2= ZutPhen Elderly Study (Physical Activity & Weight Loss). ZED3= ZutPhen Elderly Study (Physical Activity & Low BMI).
