# Supplementary material for: Comparative analysis of the association between 35 frailty scores and cardiovascular events, cancer, and total mortality in an elderly general population in England: An observational study
Source: PLoS Med. 2018 Mar 27;15(3):e1002543. doi: 10.1371/journal.pmed.1002543 (PMC5870943; doi:10.1371/journal.pmed.1002543)
Supplement: S11 Table — (DOCX) [file pmed.1002543.s012.docx]

**S11 Table.** Discriminative assessment of cancer models using Harrell's C statistic (n=4792)

| **Continuous analysis** | | | |  | **Cut-off analysis** | | | |
| --- | --- | --- | --- | --- | --- | --- | --- | --- |
| **Frailty Score** | **Delta (*100) LCI; UCI with 95%CI^1^** | **Delta (*100) LCI; UCI with 95%CI^1^** | **Delta (*100) LCI; UCI with 95%CI^1^** |  | **Frailty Score** | **Delta (*100) LCI; UCI with 95%CI^1^** | **Delta (*100) LCI; UCI with 95%CI^1^** | **Delta (*100) LCI; UCI with 95%CI^1^** |
|  | **Model 1** | **Model 2** | **Model 3** |  |  | **Model 1** | **Model 2** | **Model 3** |
| **Basic models** | **55.7 (51.7; 59.6)^2^** | **57.1 (52.8; 61.3)^2^** | **59.4 (55.3; 63.4)^2^** |  | **Basic models** | **55.7 (51.7; 59.6)^2^** | **57.1 (52.8; 61.3)^2^** | **59.4 (55.3; 63.4)^2^** |
| **Phenotype of frailty approach** | | | | | | | | |
| FS | 0.1 (-0.5; 0.7) | -0.1 (-0.7; 0.5) | 0.0 (-0.3; 0.4) |  | ZED2 frail | 0.6 (-0.4; 1.6) | 0.5 (-0.5; 1.0) | 0.4 (-0.5; 1.3) |
| FiND | 0.1 (-0.5; 0.7) | 0.2 (-0.6; 1.0) | 0.0 (-0.2; 0.2) |  | PFI frail | 0.0 (-0.3; 0.4) | 0.1 (-0.3; 1.0) | 0.1 (-0.4; 0.5) |
| SOF | 0.1 (-0.7; 0.8) | -0.1 (-0.6; 0.3) | 0.0 (-0.6; 0.5) |  | PFI pre frail | 0.2 (-0.4; 0.9) | 0.1 (-0.4; 1.0) | 0.0 (-0.3; 0.4) |
| ZED3 | 0.1 (-0.4; 0.5) | 0.0 (-0.3; 0.3) | 0.0 (-0.2; 0.3) |  | SOF frail | 0.0 (-0.5; 0.5) | -0.1 (-0.5; 1.0) | 0.0 (-0.3; 0.2) |
| PFI | 0.0 (-0.3; 0.4) | 0.1 (-0.6; 0.7) | 0.0 (-0.2; 0.2) |  | SOF pre-frail | 0.1 (-0.7; 1.0) | 0.1 (-0.5; 1.0) | 0.0 (-0.3; 0.2) |
| PHF | 0.0 (-0.9; 1.0) | -0.1 (-0.6; 0.5) | 0.0 (-0.6; 0.7) |  | ZED1 frail | 0.1 (-0.5; 0.7) | 0.0 (-0.4; 1.0) | 0.0 (-0.2; 0.2) |
| MPHF | 0.0 (-0.6; 0.6) | 0.0 (-0.4; 0.4) | 0.0 (-0.3; 0.3) |  | FS frail | 0.0 (-0.4; 0.5) | 0.0 (-0.3; 1.0) | 0.0 (-0.3; 0.3) |
| ZED1 | -0.1 (-0.6; 0.5) | -0.1 (-0.4; 0.3) | 0.0 (-0.3; 0.3) |  | FS pre- frail | 0.1 (-0.4; 0.5) | 0.0 (-0.4; 1.0) | 0.0 (-0.4; 0.4) |
| BDE | -0.1 (-0.6; 0.4) | -0.1 (-0.9; 0.8) | 0.1 (-0.5; 0.6) |  | PHF frail | 0.0 (-0.4; 0.4) | -0.1 (-0.4; 1.0) | 0.0 (-0.3; 0.2) |
| SPPB | -0.2 (-0.9; 0.5) | -0.1 (-0.4; 0.3) | 0.0 (-0.5; 0.4) |  | PHF pre-frail | 0.0 (-0.4; 0.3) | 0.0 (-0.4; 1.0) | 0.0 (-0.3; 0.2) |
| ZED2 | -0.3 (-1.1; 0.6) | 0.0 (-0.4; 0.3) | -0.3 (-0.1; 0.5) |  | ZED3 frail | 0.0 (-0.5; 0.5) | -0.1 (-0.9; 1.0) | -0.1 (-0.4; 0.2) |
|  |  |  |  |  | FiND frail | -0.1 (-1.1; 0.9) | -0.2 (-0.9; 1.0) | -0.1 (-0.8; 0.7) |
|  |  |  |  |  | SPPB frail | -0.5 (-1.4; 0.3) | -0.5 (-1.3; 1.0) | -0.3 (-0.8; 0.3) |
| **Multidimensional approach** | | | | | | | | |
| FSS | 0.2 (-0.5; 1.0) | 0.0 (-0.3; 0.3) | 0.0 (-0.3; 0.3) |  | BFI frail | 0.5 (-1.1; 2.0) | 0.7 (-0.7; 2.0) | 0.5 (-0.8; 1.8) |
| CGAST | 0.1 (-0.7; 1.0) | -0.1 (-0.3; 0.2) | 0.1 (-0.4; 0.5) |  | CGAST frail | 0.3 (-0.9; 1.5) | 0.1 (-0.8; 2.0) | 0.1 (-0.4; 0.6) |
| SDFI | 0.1 (-0.5; 0.8) | -0.1 (-0.5; 0.4) | -0.1 (-0.5; 0.3) |  | CGAST pre frail | 0.2 (-0.5; 0.9) | 0.0 (-0.5; 2.0) | 0.0 (-0.3; 0.2) |
| BFI | 0.1 (-0.3; 0.6) | -0.1 (-0.5; 0.4) | 0.1 (-0.8; 0.9) |  | G8 frail | 0.2 (-0.8; 1.2) | 0.1 (-0.4; 2.0) | -0.1 (-1.3; 1.1) |
| EFS | 0.1 (-0.9; 1.1) | 0.0 (-0.5; 0.6) | 0.0 (-0.3; 0.2) |  | FSS frail | 0.2 (-0.5; 1.0) | 0.0 (-0.4; 2.0) | 0.0 (-0.2; 0.2) |
| SPQ | 0.1 (-0.7; 0.8) | -0.2 (-0.7; 0.3) | 0.0 (-0.4; 0.4) |  | FSS pre frail | 0.2 (-0.6; 1.0) | 0.2 (-0.5; 2.0) | 0.1 (-0.6; 0.7) |
| IFQ | 0.1 (-0.6; 0.7) | -0.1 (-0.7; 0.5) | 0.0 (-0.2; 0.2) |  | SPQ frail | 0.2 (-0.6; 1.0) | 0.0 (-0.6; 2.0) | 0.1 (-0.4; 0.6) |
| HSF | 0.0 (-0.3; 0.3) | -0.1 (-0.5; 0.3) | 0.2 (-0.4; 0.7) |  | EFS frail | 0.1 (-0.5; 0.7) | 0.0 (-0.3; 2.0) | 0.0 (-0.1; 0.2) |
| GFI | 0.0 (-0.7; 0.7) | 0.0 (-0.3; 0.3) | 0.0 (-0.2; 0.2) |  | GFI frail | 0.1 (-0.6; 0.8) | 0.0 (-0.7; 2.0) | 0.0 (-0.2; 0.3) |
| TFI | 0.0 (-0.5; 0.5) | 0.0 (-0.4; 0.4) | 0.1 (-0.3; 0.5) |  | SDFI frail | 0.0 (-0.4; 0.5) | 0.1 (-0.5; 2.0) | -0.1 (-0.8; 0.5) |
| SI | 0.0 (-0.5; 0.4) | -0.1 (-0.7; 0.5) | 0.0 (-0.3; 0.3) |  | TFI frail | 0.0 (-0.4; 0.4) | 0.0 (-0.3; 2.0) | 0.1 (-0.6; 0.8) |
| CSBA | -0.1 (-0.7; 0.5) | -0.1 (-0.6; 0.3) | 0.2 (-0.5; 0.9) |  | SI frail | 0.0 (-0.3; 0.3) | 0.0 (-0.3; 2.0) | -0.1 (-0.3; 0.2) |
| G8 | -0.1 (-0.7; 0.4) | 0.0 (-0.3; 0.3) | -0.5 (-0.7; 0.7) |  | MFS frail | -0.1 (-0.6; 0.4) | 0.0 (-0.4; 2.0) | 0.0 (-0.4; 0.4) |
| MFS | -0.2 (-0.7; 0.3) | -0.2 (-0.6; 0.3) | 0.0 (-0.4; 0.5) |  | MFS pre-frail | 0.0 (-0.4; 0.4) | 0.0 (-0.4; 2.0) | 0.0 (-0.3; 0.3) |
|  |  |  |  |  | IFQ frail | 0.0 (-0.4; 0.3) | -0.1 (-0.4; 2.0) | 0.0 (-0.2; 0.2) |
|  |  |  |  |  | CSBA frail | -0.3 (-1.0; 0.3) | -0.2 (-0.8; 2.0) | 0.1 (-0.4; 0.6) |
| **Accumulation of deficits approach** | | | | | | | | |
| FI40 | 0.1 (-0.5; 0.6) | 0.0 (-0.4; 0.4) | 0.2 (-0.4; 0.8) |  | FI40 frail | 0.3 (-0.7; 1.3) | 0.1 (-0.5; 3.0) | 0.0 (-0.3; 0.3) |
| FI70 | 0.1 (-0.6; 0.7) | -0.1 (-0.8; 0.6) | 0.1 (-0.4; 0.7) |  | CGA frail | 0.1 (-0.5; 0.8) | 0.0 (-0.4; 3.0) | 0.0 (-0.4; 0.4) |
| NLTCS | 0.1 (-0.4; 0.6) | -0.3 (-0.9; 0.2) | 0.0 (-0.3; 0.2) |  | CGA pre-frail | 0.0 (-0.3; 0.3) | 0.0 (-0.4; 3.0) | 0.0 (-0.3; 0.4) |
| CGA | 0.1 (-0.5; 0.7) | 0.0 (-0.5; 0.5) | -0.1 (-0.7; 0.6) |  | FI70 frail | 0.0 (-0.4; 0.4) | -0.1 (-0.6; 3.0) | 0.3 (-0.6; 1.1) |
| FIBLSA | 0.1 (-0.5; 0.6) | 0.0 (-0.5; 0.5) | -0.1 (-0.8; 0.6) |  |  |  |  |  |
| EFIP | 0.0 (-0.5; 0.4) | -0.1 (-0.5; 0.3) | 0.0 (-0.5; 0.6) |  |  |  |  |  |
| **Disability approach** | | | | | | | | |
| WHRH | 0.2 (-0.6; 0.9) | -0.1 (-0.5; 0.4) | -0.1 (-0.2; 1.0) |  | WHRH frail | 0.2 (-0.4; 0.8) | 0.0 (-0.4; 4.0) | -0.1 (-1.2; 0.9) |
| HRCA | 0.0 (-0.5; 0.5) | -0.1 (-0.5; 0.3) | 0.0 (-0.3; 0.4) |  | VES13 frail | 0.1 (-0.5; 0.7) | 0.0 (-0.5; 4.0) | 0.0 (-0.2; 0.3) |
| VES13 | -0.1 (-0.7; 0.6) | -0.4 (-0.1; 0.4) | 0.0 (-0.2; 0.2) |  | HRCA frail | 0.0 (-0.5; 0.6) | -0.1 (-0.5; 4.0) | 0.0 (-0.2; 0.3) |
| SHCFS | -0.2 (-0.6; 0.3) | 0.0 (-0.3; 0.2) | 0.0 (-0.4; 0.3) |  | SHCFS frail | -0.1 (-0.5; 0.3) | -0.1 (-0.4; 4.0) | 0.0 (-0.3; 0.3) |

Model 1 = age and sex. Model 2 = model 1 + smoking status and maximum alcohol consumption. Model 3= Model 2 + physical activity, BMI, diabetes, hypertension, cardiovascular disease, anemia, COPD, arthritis, neuropsychiatric, depression, cognition, self-rated health & quality of life

^1^Delta = percent of improvement adding the frailty score to model. ^2^Harrel's C statistic of each model (lower confidence interval; upper confidence interval)*100.

Abbreviations frailty scores: BDE= Beaver Dam Eye Study Index. BFI= Brief Frailty Index. CGA= Comprehensive Geriatric Assessment. CGAST= Comprehensive Geriatric Assessment Screening Tests. CSBA= Conselice Study of Brain Aging Score. EFIP= Evaluative Frailty Index for Physical Activity. EFS= Edmonton Frail Scale. FI40= 40-item Frailty Index. FI70= 70-item Frailty Index (SHARE). FIBLSA= Frailty Index Beijing Longitudinal Study of Ageing. FiND= Frail Non-Disabled Questionnaire. FS= Frail Scale. FSS= Frailty Staging System. G8= G-8 Geriatric Screening Tool. GFI= Groningen Frailty Indicator. HRCA= Hebrew Rehabilitation Center for Aged Vulnerability Index. HSF= Health Status Form. IFQ= Inter-Frail Questionnaire. MFS= Modified Frailty Score. MPHF= Modified Phenotype of Frailty. NLTCS= Long Term Care Survey Frailty Index. PFI= Physical Frailty Index. PHF= Phenotype of Frailty. SDFI=, Static/Dynamic Frailty Index. SHCFS= Canadian Study of Health and Aging Clinical Frailty Scale·. SI= Screening Instrument. SOF= Study of Osteoporotic Fractures. SPPB= Short Physical Performance Battery. SPQ= Sherbrooke Postal Questionnaire. TFI= Tilburg Frailty Indicator. VES13= Vulnerable Elders Survey. WHRH= WHOAFC & self-reported health. ZED1= ZutPhen Elderly Study (Physical Activity & Low Energy). ZED2= ZutPhen Elderly Study (Physical Activity & Weight Loss). ZED3= ZutPhen Elderly Study (Physical Activity & Low BMI).
