# Supplementary material for: Comparative analysis of the association between 35 frailty scores and cardiovascular events, cancer, and total mortality in an elderly general population in England: An observational study
Source: PLoS Med. 2018 Mar 27;15(3):e1002543. doi: 10.1371/journal.pmed.1002543 (PMC5870943; doi:10.1371/journal.pmed.1002543)
Supplement: S12 Table — (DOCX) [file pmed.1002543.s013.docx]

**S12 Table.** Sensitivity analysis^1^: mortality hazard ratios of frailty scores (n=5253)

| **Continuous analysis** | | | | | **Cut-off analysis** | | | | |
| --- | --- | --- | --- | --- | --- | --- | --- | --- | --- |
|  | **HR (95% CI)** | **HR (95% CI)** | **HR (95% CI)** | **HR (95% CI)** |  | **HR (95% CI)** | **HR (95% CI)** | **HR (95% CI)** | **HR (95% CI)** |
| **Frailty Score** | **Model 0^2^** | **Model 1^3^** | **Model 2^4^** | **Model 3^5^** | **Frailty Score** | **Model 0^2^** | **Model 1^3^** | **Model 2^4^** | **Model 3^5^** |
| **Phenotype of frailty approach** | | | | | | | | | |
| MPHF | 4.5 (1.0; 6.7) | 5.1 (3.4; 7.6) | 4.4 (2.9; 6.5) | 2.5 (1.6; 4.0) | PFI frail | 1.8 (0.6; 4.9) | 2.0 (0.7; 5.3) | 1.9 (0.7; 5.1) | 1.3 (0.4; 3.6) |
| SPPB | 4.1 (1.0; 6.4) | 4.9 (3.1; 7.8) | 4.5 (2.8; 7.1) | 2.2 (1.3; 3.7) | PFI pre frail | 2.8 (1.7; 4.7) | 3.0 (1.8; 5.0) | 2.8 (1.8; 4.7) | 2.3 (1.3; 3.9) |
| PHF | 4.1 (1.0; 6.2) | 4.6 (3.1; 6.9) | 4.1 (2.7; 6.1) | 2.4 (1.5; 3.8) | FS frail | 2.2 (1.0; 4.8) | 2.4 (1.1; 5.2) | 2.2 (1.1; 4.8) | 1.4 (0.6; 3.2) |
| FS | 3.5 (1.0; 5.8) | 4.0 (2.4; 6.7) | 3.6 (2.1; 5.9) | 1.7 (1.0; 3.0) | FS pre- frail | 2.7 (1.8; 4.2) | 2.9 (1.9; 4.5) | 2.7 (1.9; 4.2) | 2.2 (1.4; 3.5) |
| FiND | 3.3 (1.0; 5.0) | 3.8 (2.5; 5.8) | 3.4 (2.2; 5.1) | 1.9 (1.2; 3.1) | SOF frail | 2.1 (1.0; 4.6) | 2.3 (1.1; 4.9) | 2.2 (1.1; 4.6) | 1.6 (0.7; 3.7) |
| SOF | 3.0 (1.0; 4.8) | 3.3 (2.1; 5.3) | 3.1 (1.9; 4.9) | 2.1 (1.3; 3.5) | SOF pre-frail | 2.7 (1.8; 4.2) | 2.9 (1.9; 4.5) | 2.7 (1.8; 4.2) | 2.4 (1.5; 3.9) |
| ZED2 | 2.8 (1.0; 4.0) | 3.1 (2.1; 4.4) | 2.9 (2.0; 4.1) | 2.0 (1.3; 2.9) | PHF frail | 2.6 (1.0; 6.6) | 2.9 (1.1; 7.3) | 2.5 (1.1; 6.5) | 1.5 (0.6; 4.1) |
| ZED3 | 2.1 (1.0; 3.3) | 2.5 (1.5; 4.0) | 2.2 (1.4; 3.6) | 1.5 (0.9; 2.6) | PHF pre-frail | 2.6 (1.2; 5.7) | 2.8 (1.3; 6.2) | 2.6 (1.3; 5.7) | 2.2 (1.0; 4.9) |
| ZED1 | 2.2 (1.0; 3.1) | 2.5 (1.7; 3.6) | 2.2 (1.5; 3.2) | 1.3 (0.9; 2.0) | ZED3 frail | 2.1 (0.2; 20.5) | 2.2 (0.2; 21.2) | 2.1 (1.4; 3.0) | 1.6 (0.2; 14.8) |
| PFI | 1.9 (1.0; 2.9) | 2.1 (1.4; 3.2) | 2.0 (1.3; 3.0) | 1.3 (0.8; 2.0) | ZED2 frail | 1.9 (0.6; 6.2) | 2.0 (0.6; 6.5) | 2.0 (0.6; 6.4) | 1.4 (0.4; 4.7) |
| BDE | 1.4 (1.0; 2.0) | 1.5 (1.1; 2.1) | 1.3 (0.9; 1.9) | 1.8 (1.2; 2.8) | ZED1 frail | 1.7 (0.6; 5.0) | 1.8 (0.6; 5.3) | 1.8 (0.6; 5.3) | 1.2 (0.4; 3.6) |
|  |  |  |  |  | SPPB frail | 1.6 (0.9; 2.9) | 1.7 (0.9; 3.0) | 1.6 (0.9; 2.9) | 1.2 (0.7; 2.3) |
|  |  |  |  |  | FiND frail | 1.5 (0.7; 2.9) | 1.6 (0.8; 3.1) | 1.5 (0.8; 2.9) | 1.1 (0.5; 2.3) |
| **Multidimensional approach** | | | | | | | | | |
| CSBA | 23.0 (2.0; 40.5) | 18.1 (10.1; 32.4) | 14.0 (7.7; 25.2) | 2.6 (1.2; 5.2) | FSS frail | 1.8 (0.9; 3.5) | 1.9 (0.9; 3.8) | 1.7 (0.9; 3.5) | 1.1 (0.5; 2.4) |
| G8 | 9.9 (2.0; 18.0) | 13.2 (7.3; 24.1) | 10.7 (5.8; 19.5) | 3.5 (1.8; 7.0) | FSS pre frail | 3.1 (2.0; 4.8) | 3.2 (2.1; 4.9) | 3.0 (2.1; 4.7) | 2.5 (1.6; 4.0) |
| EFS | 9.8 (2.0; 19.4) | 12.5 (6.3; 24.8) | 10.1 (5.1; 20.2) | 6.0 (2.7; 13.2) | CGAST frail | 2.8 (1.3; 6.0) | 3.0 (1.4; 6.6) | 2.8 (1.4; 6.0) | 1.8 (0.8; 4.1) |
| CGAST | 6.4 (2.0; 11.7) | 8.1 (4.5; 14.9) | 6.9 (3.8; 12.6) | 2.5 (1.2; 4.9) | CGAST pre frail | 3.1 (1.6; 6.1) | 3.2 (1.6; 6.3) | 3.1 (1.6; 6.0) | 2.8 (1.4; 5.6) |
| TFI | 5.2 (2.0; 8.5) | 7.3 (4.5; 12.0) | 5.9 (3.6; 9.7) | 3.2 (1.7; 5.8) | MFS frail | 1.3 (0.4; 3.7) | 2.6 (0.9; 7.9) | 2.3 (0.9; 6.9) | 1.7 (0.6; 5.1) |
| GFI | 5.1 (2.0; 9.0) | 6.4 (3.6; 11.4) | 5.3 (3.0; 9.5) | 1.6 (0.8; 3.2) | MFS pre-frail | 1.3 (0.5; 3.5) | 2.8 (1.0; 7.6) | 2.5 (1.0; 7.0) | 2.3 (0.8; 6.4) |
| SDFI | 3.7 (2.0; 6.2) | 6.4 (3.8; 10.8) | 5.0 (3.0; 8.5) | 1.5 (0.8; 2.7) | G8 frail | 2.1 (1.2; 3.7) | 2.2 (1.2; 3.9) | 2.0 (1.2; 3.6) | 1.4 (0.7; 2.6) |
| IFQ | 4.4 (2.0; 7.9) | 5.8 (3.3; 10.4) | 4.8 (2.7; 8.7) | 1.9 (1.0; 3.5) | CSBA frail | 2.1 (1.2; 3.6) | 1.9 (1.1; 3.3) | 1.7 (1.1; 3.0) | 1.2 (0.7; 2.1) |
| MFS | 5.2 (2.0; 8.0) | 5.8 (3.8; 8.9) | 5.0 (3.2; 7.7) | 3.1 (2.0; 4.8) | IFQ frail | 1.8 (0.5; 6.7) | 1.9 (0.5; 7.3) | 1.8 (0.5; 6.9) | 1.3 (0.4; 5.1) |
| HSF | 3.9 (2.0; 6.5) | 4.2 (2.5; 7.1) | 3.7 (2.2; 6.2) | 1.3 (0.7; 2.3) | EFS frail | 1.8 (0.6; 5.2) | 1.9 (0.6; 5.6) | 1.8 (0.6; 5.3) | 1.3 (0.4; 4.0) |
| BFI | 2.2 (2.0; 3.5) | 3.0 (1.9; 4.8) | 2.5 (1.6; 4.0) | 1.3 (0.8; 2.1) | TFI frail | 1.7 (1.0; 2.9) | 1.9 (1.1; 3.2) | 1.7 (1.1; 3.0) | 1.4 (0.8; 2.5) |
| FSS | 2.5 (2.0; 3.9) | 2.8 (1.8; 4.3) | 2.5 (1.6; 3.8) | 1.1 (0.7; 1.8) | SDFI frail | 1.5 (0.9; 2.7) | 1.8 (1.0; 3.1) | 1.6 (1.0; 2.8) | 1.2 (0.7; 2.2) |
| SI | 2.1 (2.0; 4.1) | 2.7 (1.4; 5.2) | 2.4 (1.2; 4.6) | 0.8 (0.4; 1.6) | GFI frail | 1.5 (0.9; 2.6) | 1.6 (0.9; 2.7) | 1.5 (0.9; 2.6) | 1.0 (0.6; 1.9) |
| SPQ | 1.8 (2.0; 3.2) | 2.3 (1.3; 4.1) | 2.0 (1.1; 3.6) | 0.9 (0.5; 1.7) | BFI frail | 1.2 (0.5; 2.8) | 1.4 (0.6; 3.1) | 1.3 (0.6; 2.9) | 1.0 (0.4; 2.3) |
|  |  |  |  |  | SI frail | 1.2 (0.4; 4.0) | 1.3 (0.4; 4.3) | 1.3 (1.1; 1.6) | 0.9 (0.3; 2.8) |
|  |  |  |  |  | SPQ frail | 1.1 (0.6; 2.0) | 1.2 (0.7; 2.2) | 1.2 (1.0; 1.4) | 0.9 (0.5; 1.7) |
| **Accumulation of deficits approach** | | | | | | | | | |
| FI40 | 7.9 (3.0; 15.3) | 11.0 (5.7; 21.2) | 9.1 (4.6; 17.7) | 8.2 (4.7; 14.1) | CGA frail | 1.9 (1.0; 3.9) | 2.2 (1.1; 4.5) | 2.1 (1.1; 4.1) | 1.6 (0.7; 3.4) |
| CGA | 6.9 (3.0; 15.3) | 10.8 (4.9; 24.2) | 8.8 (3.9; 19.8) | 3.8 (1.5; 9.6) | CGA pre-frail | 2.8 (1.8; 4.4) | 3.1 (2.0; 4.8) | 2.9 (2.0; 4.5) | 2.7 (1.7; 4.3) |
| FI70 | 6.5 (3.0; 12.3) | 9.6 (5.1; 18.4) | 7.9 (4.1; 15.2) | 5.4 (2.5; 11.6) | FI70 frail | 1.7 (1.0; 3.0) | 1.9 (1.1; 3.3) | 1.8 (1.1; 3.1) | 1.6 (0.9; 2.8) |
| EFIP | 5.7 (3.0; 10.9) | 7.4 (3.9; 14.2) | 6.1 (3.1; 11.7) | 3.3 (1.5; 7.3) | FI40 frail | 1.7 (1.0; 2.9) | 1.8 (1.1; 3.1) | 1.7 (1.1; 3.0) | 1.7 (1.1; 2.4) |
| NLTCS | 6.1 (3.0; 15.3) | 7.1 (2.8; 17.8) | 5.9 (2.4; 14.9) | 1.0 (0.4; 2.9) |  |  |  |  |  |
| FIBLSA | 4.6 (3.0; 9.6) | 5.8 (2.8; 12.1) | 4.9 (2.4; 10.3) | 1.2 (0.5; 2.7) |  |  |  |  |  |
| **Disability approach** | | | | | | | | | |
| VES13 | 3.6 (4.0; 6.0) | 4.6 (2.8; 7.6) | 4.0 (2.4; 6.6) | 2.1 (1.2; 3.7) | HRCA frail | 1.6 (0.9; 2.7) | 1.8 (1.1; 3.1) | 1.7 (1.1; 2.9) | 1.2 (0.7; 2.2) |
| HRCA | 3.1 (4.0; 5.7) | 4.0 (2.2; 7.2) | 3.5 (1.9; 6.3) | 1.3 (0.7; 2.6) | VES13 frail | 1.5 (0.9; 2.7) | 1.7 (1.0; 2.9) | 1.6 (0.9; 2.8) | 1.3 (0.7; 2.3) |
| WHRH | 2.9 (4.0; 4.9) | 3.4 (2.0; 5.8) | 3.1 (1.8; 5.2) | 1.9 (1.0; 3.5) | SHCFS frail | 1.6 (0.8; 3.1) | 1.7 (0.8; 3.3) | 1.6 (0.8; 3.1) | 1.1 (0.5; 2.2) |
| SHCFS | 2.6 (4.0; 3.9) | 2.9 (1.9; 4.4) | 2.6 (1.7; 4.0) | 1.1 (0.7; 1.8) | WHRH frail | 1.5 (0.8; 2.8) | 1.6 (0.9; 3.1) | 1.6 (0.9; 2.9) | 1.0 (0.5; 1.9) |

^1^Sensitivity analysis: excluding participants with events the first year of follow-up; ^2^Model 0= Crude models. ^3^Model 1= HR adjusted by sex. ^4^Model 2= Model 1 + smoking status and alcohol consumption. ^5^Model 3= Model 2 + physical activity, BMI, diabetes, hypertension, cardiovascular, cancer, anemia, COPD, arthritis, neuropsychiatric, depression, cognition, self-rated health & quality of life.

Abbreviations frailty scores: BDE= Beaver Dam Eye Study Index. BFI= Brief Frailty Index. CGA= Comprehensive Geriatric Assessment. CGAST= Comprehensive Geriatric Assessment Screening Tests. CSBA= Conselice Study of Brain Aging Score. EFIP= Evaluative Frailty Index for Physical Activity. EFS= Edmonton Frail Scale. FI40= 40-item Frailty Index. FI70= 70-item Frailty Index (SHARE). FIBLSA= Frailty Index Beijing Longitudinal Study of Ageing. FiND= Frail Non-Disabled Questionnaire. FS= Frail Scale. FSS= Frailty Staging System. G8= G-8 Geriatric Screening Tool. GFI= Groningen Frailty Indicator. HRCA= Hebrew Rehabilitation Center for Aged Vulnerability Index. HSF= Health Status Form. IFQ= Inter-Frail Questionnaire. MFS= Modified Frailty Score. MPHF= Modified Phenotype of Frailty. NLTCS= Long Term Care Survey Frailty Index. PFI= Physical Frailty Index. PHF= Phenotype of Frailty. SDFI=, Static/Dynamic Frailty Index. SHCFS= Canadian Study of Health and Aging Clinical Frailty Scale·. SI= Screening Instrument. SOF= Study of Osteoporotic Fractures. SPPB= Short Physical Performance Battery. SPQ= Sherbrooke Postal Questionnaire. TFI= Tilburg Frailty Indicator. VES13= Vulnerable Elders Survey. WHRH= WHOAFC & self-reported health. ZED1= ZutPhen Elderly Study (Physical Activity & Low Energy). ZED2= ZutPhen Elderly Study (Physical Activity & Weight Loss). ZED3= ZutPhen Elderly Study (Physical Activity & Low BMI).
