# Supplementary material for: Comparative analysis of the association between 35 frailty scores and cardiovascular events, cancer, and total mortality in an elderly general population in England: An observational study
Source: PLoS Med. 2018 Mar 27;15(3):e1002543. doi: 10.1371/journal.pmed.1002543 (PMC5870943; doi:10.1371/journal.pmed.1002543)
Supplement: S13 Table — (DOCX) [file pmed.1002543.s014.docx]

**S13 Table.** Mortality hazard ratios of frailty scores in men (n=2377) calculated at median time follow-up (3.5 years)

| **Continuous analysis** | | | | **Cut-off analysis** | | | |
| --- | --- | --- | --- | --- | --- | --- | --- |
|  | **HR (95% CI)** | **HR (95% CI)** | **HR (95% CI)** |  | **HR (95% CI)** | **HR (95% CI)** | **HR (95% CI)** |
| **Frailty Score** | **Model 0^1^** | **Model 2^2^** | **Model 3^3^** | **Frailty Score** | **Model 0^1^** | **Model 2^2^** | **Model 3^3^** |
| **Phenotype of frailty approach** | | | | | | | |
| MPHF | 7.7 (5.0; 12.0) | 6.6 (4.2; 10.3) | 3.6 (2.3; 6.1) | SOF frail | 3.2 (1.5; 7.2) | 3.0 (1.4; 6.7) | 2.1 (0.9; 5.3) |
| FS | 7.0 (4.2; 11.7) | 6.2 (3.7; 10.3) | 2.8 (1.2; 5.0) | SOF pre-frail | 2.7 (1.6; 4.4) | 2.6 (1.6; 4.2) | 2.2 (1.2; 3.8) |
| SPPB | 6.7 (3.9; 11.5) | 6.1 (3.5; 10.6) | 2.3 (1.2; 4.4) | PHF frail | 3.4 (1.2; 9.2) | 3.0 (1.1; 8.3) | 1.6 (0.5; 4.8) |
| PHF | 6.5 (4.2; 10.0) | 5.5 (3.6; 8.6) | 2.7 (1.2; 4.7) | ZED3 frail | 3.3 (1.9; 5.7) | 2.6 (1.5; 4.5) | 1.7 (0.2; 13.4) |
| FiND | 6.0 (3.9; 9.2) | 5.3 (3.4; 8.1) | 2.6 (1.2; 4.5) | ZED1 frail | 2.6 (2.0; 3.5) | 2.6 (1.9; 3.4) | 1.7 (0.6; 4.4) |
| SOF | 5.4 (3.4; 8.7) | 4.9 (3.1; 8.0) | 2.9 (1.2; 5.2) | PFI frail | 2.3 (0.7; 7.2) | 2.3 (0.7; 7.2) | 1.3 (0.4; 4.5) |
| ZED2 | 4.4 (2.9; 6.5) | 3.9 (2.6; 5.8) | 2.5 (1.2; 3.9) | PFI pre frail | 2.3 (1.3; 4.2) | 2.2 (1.2; 4.0) | 1.7 (0.9; 3.4) |
| ZED3 | 4.4 (2.7; 7.4) | 3.7 (2.2; 6.1) | 2.2 (1.2; 3.8) | ZED2 frail | 2.3 (1.6; 3.3) | 2.1 (1.5; 3.1) | 1.5 (0.4; 4.9) |
| ZED1 | 4.0 (2.7; 5.7) | 3.6 (2.5; 5.2) | 1.8 (1.1; 2.9) | FS frail | 2.1 (1.0; 4.4) | 1.9 (0.9; 4.1) | 1.9 (0.8; 4.5) |
| PFI | 2.5 (1.6; 3.9) | 2.4 (1.5; 3.8) | 1.4 (0.1; 2.2) | FS pre- frail | 2.3 (1.4; 3.8) | 2.2 (1.3; 3.7) | 1.7 (1.0; 2.9) |
| BDE | 2.3 (1.5; 3.7) | 2.0 (1.3; 3.3) | 2.0 (1.2; 3.2) | PHF pre-frail | 2.0 (0.8; 4.7) | 1.9 (0.8; 4.4) | 1.5 (0.6; 3.7) |
|  |  |  |  | SPPB frail | 1.7 (0.9; 3.1) | 1.6 (0.8; 3.0) | 1.2 (0.6; 2.3) |
|  |  |  |  | FiND frail | 1.5 (0.7; 3.1) | 1.4 (0.6; 2.9) | 1.0 (0.5; 2.2) |
| **Multidimensional approach** | | | | | | | |
| EFS | 30.5 (14.6; 63.8) | 26.1 (12.3; 55.5) | 13.8 (5.13; 33.9) | FSS frail | 3.4 (1.6; 7.4) | 3.2 (1.5; 6.9) | 1.0 (0.4; 2.4) |
| G8 | 30.1 (15.0; 60.6) | 22.7 (11.1; 46.1) | 5.3 (2.5; 12.5) | FSS pre frail | 2.3 (1.4; 3.8) | 2.2 (1.3; 3.6) | 1.7 (1.0; 2.9) |
| CSBA | 28.2 (13.9; 57.2) | 20.5 (10.0; 42.1) | 2.4 (1.2; 6.0) | EFS frail | 2.7 (2.0; 3.6) | 2.5 (1.9; 3.4) | 1.8 (0.6; 5.3) |
| CGAST | 15.5 (7.9; 30.4) | 12.9 (6.5; 25.5) | 4.3 (1.4; 9.8) | MFS frail | 1.8 (0.5; 6.3) | 2.6 (0.8; 9.0) | 1.8 (0.5; 6.4) |
| TFI | 16.1 (9.0; 28.7) | 12.8 (7.0; 23.1) | 7.5 (3.7; 15.7) | MFS pre-frail | 1.3 (0.4; 4.3) | 2.0 (0.6; 6.3) | 1.8 (0.6; 5.8) |
| GFI | 13.4 (7.0; 25.7) | 11.1 (5.7; 21.6) | 3.0 (1.3; 6.8) | CGAST frail | 2.8 (1.3; 5.7) | 2.6 (1.2; 5.4) | 1.8 (0.7; 4.7) |
| SDFI | 13.3 (7.2; 24.5) | 10.4 (5.5; 19.4) | 2.4 (1.2; 5.1) | CGAST pre frail | 2.3 (1.4; 3.8) | 2.2 (1.3; 3.6) | 2.0 (0.9; 4.6) |
| IFQ | 11.1 (5.8; 21.1) | 8.8 (4.6; 17.0) | 3.2 (1.3; 6.6) | G8 frail | 2.4 (1.2; 4.5) | 2.2 (1.1; 4.1) | 1.3 (0.6; 2.8) |
| MFS | 7.8 (4.7; 12.8) | 6.5 (3.9; 10.9) | 3.7 (2.3; 6.3) | SPQ frail | 1.2 (1.0; 1.4) | 1.1 (0.9; 2.4) | 0.9 (0.5; 1.8) |
| HSF | 6.3 (3.6; 11.0) | 5.7 (3.2; 10.1) | 1.4 (0.1; 2.9) | TFI frail | 2.2 (1.2; 3.8) | 2.0 (1.2; 3.5) | 1.6 (0.9; 3.0) |
| SI | 5.8 (2.9; 11.6) | 5.2 (2.6; 10.4) | 1.5 (0.1; 3.4) | CSBA frail | 2.0 (1.1; 3.7) | 1.9 (1.0; 3.4) | 1.1 (0.6; 2.3) |
| BFI | 5.2 (3.1; 8.9) | 4.4 (2.5; 7.5) | 2.2 (1.2; 3.9) | SDFI frail | 2.0 (1.1; 3.6) | 1.8 (1.0; 3.3) | 1.3 (0.7; 2.4) |
| FSS | 3.8 (2.3; 6.3) | 3.4 (2.0; 5.5) | 1.1 (0.1; 1.9) | SI frail | 1.9 (0.5; 7.6) | 1.8 (0.4; 7.2) | 1.0 (0.2; 4.3) |
| SPQ | 2.8 (1.4; 5.5) | 2.3 (1.2; 4.6) | 0.9 (0.0; 1.8) | BFI frail | 1.9 (0.8; 4.7) | 1.8 (0.7; 4.4) | 1.3 (0.5; 3.3) |
|  |  |  |  | GFI frail | 1.9 (1.0; 3.3) | 1.8 (1.0; 3.1) | 1.2 (0.6; 2.2) |
|  |  |  |  | IFQ frail | 1.3 (0.7; 2.4) | 1.2 (0.6; 2.3) | 1.7 (0.4; 7.2) |
| **Accumulation of deficits approach** | | | | | | | |
| CGA | 27.2 (11.0; 67.1) | 22.4 (8.9; 56.3) | 8.0 (2.8; 23.5) | CGA frail | 3.3 (1.4; 7.8) | 3.0 (1.3; 7.3) | 1.9 (0.8; 4.2) |
| FI40 | 23.2 (11.1; 48.3) | 19.7 (9.3; 41.7) | 11.2 (5.11; 23.3) | CGA pre-frail | 2.4 (1.1; 5.4) | 2.3 (1.0; 5.2) | 2.0 (1.1; 3.4) |
| FI70 | 21.0 (10.2; 42.9) | 17.8 (8.5; 37.1) | 13.2 (5.13; 32.2) | FI40 frail | 2.0 (1.2; 3.5) | 1.9 (1.1; 3.4) | 1.6 (1.0; 2.5) |
| EFIP | 15.8 (7.7; 32.1) | 13.1 (6.4; 27.1) | 6.7 (2.6; 16.2) | FI70 frail | 2.0 (1.2; 3.5) | 1.9 (1.1; 3.3) | 1.6 (0.8; 3.0) |
| NLTCS | 12.1 (4.5; 32.9) | 10.2 (3.7; 28.1) | 1.0 (0.1; 3.3) |  |  |  |  |
| FIBLSA | 10.1 (4.5; 22.7) | 8.7 (3.8; 19.8) | 1.2 (0.1; 3.2) |  |  |  |  |
| **Disability approach** | | | | | | | |
| VES13 | 7.5 (4.3; 13.1) | 6.5 (3.7; 11.4) | 2.6 (1.2; 5.2) | HRCA frail | 2.1 (1.2; 3.7) | 2.0 (1.1; 3.4) | 1.2 (0.6; 2.4) |
| HRCA | 6.7 (3.6; 12.6) | 6.0 (3.2; 11.4) | 1.6 (0.1; 3.5) | WHRH frail | 2.0 (1.1; 3.7) | 1.9 (1.0; 3.6) | 1.0 (0.5; 2.2) |
| WHRH | 5.1 (3.0; 8.7) | 4.7 (2.7; 8.0) | 2.5 (1.2; 4.8) | SHCFS frail | 2.0 (1.1; 3.8) | 1.8 (1.5; 2.2) | 1.2 (0.6; 2.4) |
| SHCFS | 4.4 (2.9; 6.8) | 4.0 (2.6; 6.2) | 1.5 (0.1; 2.6) | VES13 frail | 2.0 (1.1; 3.5) | 1.9 (1.0; 3.3) | 1.4 (0.7; 2.6) |

^1^Model 0= Crude models. ^2^Model 2= Model 1 + smoking status and alcohol consumption. ^3^Model 3= Model 2 + physical activity, BMI, diabetes, hypertension, cardiovascular, cancer, anemia, COPD, arthritis, neuropsychiatric, depression, cognition, self-rated health & quality of life.

Models were fitted using age as time scale, with time 0 = age at entry of study and time 1 =age at event or censoring date.

Abbreviations frailty scores: BDE= Beaver Dam Eye Study Index. BFI= Brief Frailty Index. CGA= Comprehensive Geriatric Assessment. CGAST= Comprehensive Geriatric Assessment Screening Tests. CSBA= Conselice Study of Brain Aging Score. EFIP= Evaluative Frailty Index for Physical Activity. EFS= Edmonton Frail Scale. FI40= 40-item Frailty Index. FI70= 70-item Frailty Index (SHARE). FIBLSA= Frailty Index Beijing Longitudinal Study of Ageing. FiND= Frail Non-Disabled Questionnaire. FS= Frail Scale. FSS= Frailty Staging System. G8= G-8 Geriatric Screening Tool. GFI= Groningen Frailty Indicator. HRCA= Hebrew Rehabilitation Center for Aged Vulnerability Index. HSF= Health Status Form. IFQ= Inter-Frail Questionnaire. MFS= Modified Frailty Score. MPHF= Modified Phenotype of Frailty. NLTCS= Long Term Care Survey Frailty Index. PFI= Physical Frailty Index. PHF= Phenotype of Frailty. SDFI=, Static/Dynamic Frailty Index. SHCFS= Canadian Study of Health and Aging Clinical Frailty Scale·. SI= Screening Instrument. SOF= Study of Osteoporotic Fractures. SPPB= Short Physical Performance Battery. SPQ= Sherbrooke Postal Questionnaire. TFI= Tilburg Frailty Indicator. VES13= Vulnerable Elders Survey. WHRH= WHOAFC & self-reported health. ZED1= ZutPhen Elderly Study (Physical Activity & Low Energy). ZED2= ZutPhen Elderly Study (Physical Activity & Weight Loss). ZED3= ZutPhen Elderly Study (Physical Activity & Low BMI).
