# Supplementary material for: Comparative analysis of the association between 35 frailty scores and cardiovascular events, cancer, and total mortality in an elderly general population in England: An observational study
Source: PLoS Med. 2018 Mar 27;15(3):e1002543. doi: 10.1371/journal.pmed.1002543 (PMC5870943; doi:10.1371/journal.pmed.1002543)
Supplement: S14 Table — (DOCX) [file pmed.1002543.s015.docx]

**S14 Table.** Mortality hazard ratios of frailty scores in women (n=2917) calculated at median time follow-up (3.5 years)

| **Continuous analysis** | | | | **Cut-off analysis** | | | |
| --- | --- | --- | --- | --- | --- | --- | --- |
|  | **HR (95% CI)** | **HR (95% CI)** | **HR (95% CI)** |  | **HR (95% CI)** | **HR (95% CI)** | **HR (95% CI)** |
| **Frailty Score** | **Model 0^1^** | **Model 2^2^** | **Model 3^3^** | **Frailty Score** | **Model 0^1^** | **Model 2^2^** | **Model 3^3^** |
| **Phenotype of frailty approach** | | | | | | | |
| MPHF | 4.7 (2.7; 8.0) | 6.6 (4.2; 10.3) | 2.3 (1.2; 4.3) | PHF frail | 4.3 (0.7; 25.9) | 3.8 (0.6; 23.2) | 2.5 (0.4; 15.9) |
| FS | 3.3 (1.7; 6.3) | 6.2 (3.7; 10.3) | 1.3 (0.6; 2.7) | PHF pre-frail | 4.2 (0.8; 22.1) | 3.8 (0.7; 20.0) | 3.1 (0.6; 17.0) |
| SPPB | 5.6 (3.1; 10.1) | 6.1 (3.5; 10.6) | 2.8 (1.4; 5.4) | PFI frail | 2.2 (0.7; 7.0) | 2.0 (0.6; 6.6) | 1.5 (0.4; 5.2) |
| PHF | 4.9 (2.9; 8.2) | 5.5 (3.6; 8.6) | 3.1 (1.7; 5.8) | PFI pre frail | 2.8 (1.5; 5.2) | 2.6 (1.4; 4.8) | 2.2 (1.1; 4.2) |
| FiND | 3.3 (1.9; 5.8) | 5.3 (3.4; 8.1) | 1.8 (0.9; 3.4) | FiND frail | 2.7 (0.7; 10.7) | 2.5 (0.6; 10.0) | 1.8 (0.4; 7.4) |
| SOF | 2.8 (1.6; 5.0) | 4.9 (3.1; 8.0) | 2.0 (1.0; 3.7) | FS frail | 2.1 (0.8; 5.8) | 2.0 (0.7; 5.4) | 1.1 (0.4; 3.4) |
| ZED2 | 3.2 (2.0; 5.0) | 3.9 (2.6; 5.8) | 2.2 (1.3; 3.5) | FS pre- frail | 2.6 (1.5; 4.8) | 2.4 (1.3; 4.4) | 2.0 (1.1; 3.7) |
| ZED3 | 2.2 (1.2; 4.1) | 3.7 (2.2; 6.1) | 1.7 (0.9; 3.2) | ZED2 frail | 2.1 (0.6; 7.2) | 2.2 (0.6; 7.3) | 1.7 (0.5; 5.9) |
| ZED1 | 2.0 (1.2; 3.2) | 3.6 (2.5; 5.2) | 1.2 (0.7; 2.2) | SPPB frail | 2.2 (0.9; 5.5) | 2.1 (0.9; 5.2) | 1.6 (0.6; 4.2) |
| PFI | 2.3 (1.4; 3.7) | 2.4 (1.5; 3.8) | 1.5 (0.9; 2.6) | SOF frail | 2.0 (0.8; 5.2) | 1.9 (0.7; 4.9) | 1.5 (0.5; 4.2) |
| BDE | 1.8 (1.1; 2.9) | 2.0 (1.3; 3.3) | 2.4 (1.4; 4.2) | SOF pre-frail | 2.2 (1.2; 4.0) | 2.1 (1.2; 3.7) | 2.0 (1.1; 3.7) |
|  |  |  |  | ZED3 frail | 1.8 (0.0; 68.3) | 1.9 (0.1; 69.9) | 1.6 (0.0; 51.5) |
|  |  |  |  | ZED1 frail | 1.5 (0.4; 5.7) | 1.6 (0.4; 5.9) | 1.1 (0.3; 4.4) |
| **Multidimensional approach** | | | | | | | |
| EFS | 9.9 (4.2; 23.6) | 26.1 (12.3; 55.5) | 4.6 (1.7; 12.8) | MFS frail | 1.9 (0.2; 17.6) | 3.2 (0.4; 29.8) | 2.4 (0.3; 22.4) |
| G8 | 11.3 (5.2; 24.7) | 22.7 (11.1; 46.1) | 4.1 (1.7; 9.9) | MFS pre-frail | 1.8 (0.2; 16.2) | 3.2 (0.4; 28.0) | 2.9 (0.3; 25.0) |
| CSBA | 23.1 (10.0; 53.1) | 20.5 (10.0; 42.1) | 4.2 (1.5; 11.7) | CGAST frail | 3.5 (1.0; 12.1) | 3.2 (0.9; 10.9) | 2.0 (0.5; 7.3) |
| CGAST | 6.7 (3.1; 14.8) | 12.9 (6.5; 25.5) | 1.7 (0.7; 4.2) | CGAST pre frail | 3.4 (1.1; 10.9) | 3.2 (1.0; 10.3) | 2.9 (0.9; 9.3) |
| TFI | 5.5 (2.9; 10.5) | 12.8 (7.0; 23.1) | 2.2 (1.0; 5.0) | FSS frail | 2.1 (0.8; 5.2) | 1.9 (0.8; 4.8) | 1.3 (0.5; 3.5) |
| GFI | 5.1 (2.4; 10.9) | 11.1 (5.7; 21.6) | 1.1 (0.4; 2.8) | FSS pre frail | 3.4 (1.8; 6.3) | 3.2 (1.7; 5.9) | 2.7 (1.4; 5.2) |
| SDFI | 4.7 (2.3; 9.5) | 10.4 (5.5; 19.4) | 1.1 (0.5; 2.5) | G8 frail | 2.6 (1.0; 6.5) | 2.4 (1.0; 6.0) | 1.7 (0.6; 4.5) |
| IFQ | 4.9 (2.3; 10.5) | 8.8 (4.6; 17.0) | 1.5 (0.6; 3.5) | CSBA frail | 2.1 (1.0; 4.6) | 1.9 (0.9; 4.2) | 1.4 (0.6; 3.1) |
| MFS | 6.8 (3.8; 12.2) | 6.5 (3.9; 10.9) | 3.9 (2.1; 7.3) | IFQ frail | 1.9 (0.5; 7.8) | 1.8 (0.4; 7.5) | 1.3 (0.3; 5.6) |
| HSF | 4.9 (2.5; 9.4) | 5.7 (3.2; 10.1) | 1.7 (0.8; 3.7) | TFI frail | 1.9 (0.9; 4.0) | 1.8 (0.9; 3.7) | 1.4 (0.7; 3.1) |
| SI | 1.9 (0.8; 4.4) | 5.2 (2.6; 10.4) | 0.5 (0.2; 1.3) | SDFI frail | 1.9 (0.9; 4.0) | 1.7 (0.8; 3.7) | 1.3 (0.6; 2.9) |
| BFI | 2.4 (1.3; 4.3) | 4.4 (2.5; 7.5) | 0.9 (0.5; 1.8) | EFS frail | 1.7 (0.6; 5.3) | 1.6 (0.5; 5.0) | 1.2 (0.4; 4.0) |
| FSS | 2.8 (1.6; 5.0) | 3.4 (2.0; 5.5) | 1.2 (0.6; 2.3) | GFI frail | 1.6 (0.8; 3.2) | 1.5 (0.7; 3.0) | 1.0 (0.5; 2.2) |
| SPQ | 2.7 (1.3; 5.7) | 2.3 (1.2; 4.6) | 1.2 (0.5; 2.7) | SPQ frail | 1.3 (0.6; 2.8) | 1.2 (0.6; 2.7) | 1.0 (0.5; 2.2) |
|  |  |  |  | BFI frail | 1.2 (0.4; 3.3) | 1.1 (0.4; 3.1) | 0.9 (0.3; 2.5) |
|  |  |  |  | SI frail | 1.2 (0.3; 4.6) | 1.1 (0.3; 4.5) | 0.8 (0.2; 3.3) |
| **Accumulation of deficits approach** | | | | | | | |
| CGA | 8.9 (3.2; 24.6) | 22.4 (8.9; 56.3) | 3.1 (0.9; 10.4) | CGAtot | 2.3 (0.9; 5.9) | 2.1 (0.9; 5.4) | 1.6 (0.6; 4.5) |
| FI40 | 9.6 (4.1; 22.3) | 19.7 (9.3; 41.7) | 6.4 (3.0; 13.8) | CGAtotpre | 3.1 (1.6; 6.0) | 2.9 (1.5; 5.6) | 2.7 (1.4; 5.2) |
| FI70 | 7.9 (3.4; 18.1) | 17.8 (8.5; 37.1) | 3.9 (1.4; 10.9) | FI70tot | 2.1 (1.0; 4.3) | 2.0 (1.0; 4.0) | 1.7 (0.8; 3.7) |
| EFIP | 6.3 (2.7; 14.6) | 13.1 (6.4; 27.1) | 2.9 (1.0; 8.0) | FI40tot | 2.0 (1.0; 3.9) | 1.8 (0.9; 3.7) | 1.7 (1.0; 3.0) |
| NLTCS | 8.8 (2.7; 27.9) | 10.2 (3.7; 28.1) | 2.0 (0.5; 7.6) |  |  |  |  |
| FIBLSA | 6.0 (2.4; 15.1) | 8.7 (3.8; 19.8) | 1.6 (0.6; 4.9) |  |  |  |  |
| **Disability approach** | | | | | | | |
| VES13 | 4.4 (2.3; 8.3) | 6.5 (3.7; 11.4) | 2.6 (1.2; 5.4) | HRCAtot | 1.8 (0.9; 3.6) | 1.7 (0.8; 3.4) | 1.3 (0.6; 2.7) |
| HRCA | 3.7 (1.8; 7.9) | 6.0 (3.2; 11.4) | 1.4 (0.6; 3.3) | VES13tot | 1.7 (0.8; 3.5) | 1.6 (0.8; 3.3) | 1.4 (0.6; 2.9) |
| WHRH | 3.4 (1.8; 6.6) | 4.7 (2.7; 8.0) | 2.1 (1.0; 4.5) | SHCFStot | 1.6 (0.7; 3.7) | 1.5 (0.7; 3.6) | 1.1 (0.4; 2.6) |
| SHCFS | 2.8 (1.6; 4.8) | 4.0 (2.6; 6.2) | 1.1 (0.6; 2.2) | DFStot | 1.6 (0.7; 3.4) | 1.5 (0.7; 3.2) | 1.2 (0.5; 2.8) |

^1^Model 0= Crude models. ^2^Model 2= Model 1 + smoking status and alcohol consumption. ^3^Model 3= Model 2 + physical activity, BMI, diabetes, hypertension, cardiovascular, cancer, anemia, COPD, arthritis, neuropsychiatric, depression, cognition, self-rated health & quality of life.

Models were fitted using age as time scale, with time 0 = age at entry of study and time 1 =age at event or censoring date.

Abbreviations frailty scores: BDE= Beaver Dam Eye Study Index. BFI= Brief Frailty Index. CGA= Comprehensive Geriatric Assessment. CGAST= Comprehensive Geriatric Assessment Screening Tests. CSBA= Conselice Study of Brain Aging Score. EFIP= Evaluative Frailty Index for Physical Activity. EFS= Edmonton Frail Scale. FI40= 40-item Frailty Index. FI70= 70-item Frailty Index (SHARE). FIBLSA= Frailty Index Beijing Longitudinal Study of Ageing. FiND= Frail Non-Disabled Questionnaire. FS= Frail Scale. FSS= Frailty Staging System. G8= G-8 Geriatric Screening Tool. GFI= Groningen Frailty Indicator. HRCA= Hebrew Rehabilitation Center for Aged Vulnerability Index. HSF= Health Status Form. IFQ= Inter-Frail Questionnaire. MFS= Modified Frailty Score. MPHF= Modified Phenotype of Frailty. NLTCS= Long Term Care Survey Frailty Index. PFI= Physical Frailty Index. PHF= Phenotype of Frailty. SDFI=, Static/Dynamic Frailty Index. SHCFS= Canadian Study of Health and Aging Clinical Frailty Scale·. SI= Screening Instrument. SOF= Study of Osteoporotic Fractures. SPPB= Short Physical Performance Battery. SPQ= Sherbrooke Postal Questionnaire. TFI= Tilburg Frailty Indicator. VES13= Vulnerable Elders Survey. WHRH= WHOAFC & self-reported health. ZED1= ZutPhen Elderly Study (Physical Activity & Low Energy). ZED2= ZutPhen Elderly Study (Physical Activity & Weight Loss). ZED3= ZutPhen Elderly Study (Physical Activity & Low BMI).
