# Supplementary material for: Comparative analysis of the association between 35 frailty scores and cardiovascular events, cancer, and total mortality in an elderly general population in England: An observational study
Source: PLoS Med. 2018 Mar 27;15(3):e1002543. doi: 10.1371/journal.pmed.1002543 (PMC5870943; doi:10.1371/journal.pmed.1002543)
Supplement: S15 Table — (DOCX) [file pmed.1002543.s016.docx]

**S15 Table.** Mortality hazard ratios of frailty scores in participants older than 70 years (n=2536) calculated at median time follow-up (3.5 years)

| **Continuous analysis** | | | | | **Cut-off analysis** | | | | |
| --- | --- | --- | --- | --- | --- | --- | --- | --- | --- |
|  | **HR (95% CI)** | **HR (95% CI)** | **HR (95% CI)** | **HR (95% CI)** |  | **HR (95% CI)** | **HR (95% CI)** | **HR (95% CI)** | **HR (95% CI)** |
| **Frailty Score** | **Model 0^1^** | **Model 1^2^** | **Model 2^3^** | **Model 3^4^** | **Frailty Score** | **Model 0^1^** | **Model 1^2^** | **Model 2^3^** | **Model 3^4^** |
| **Phenotype of frailty approach** | | | | | | | | | |
| SPPB | 4.1 (2.6; 6.3) | 5.1 (3.2; 8.0) | 4.8 (3.1; 7.6) | 2.7 (1.6; 4.5) | FS frail | 2.2 (1.1; 4.5) | 2.4 (1.2; 4.9) | 2.3 (0.1; 4.7) | 1.5 (0.7; 3.3) |
| MPHF | 4.3 (2.9; 6.3) | 4.9 (3.3; 7.2) | 4.3 (2.9; 6.4) | 2.6 (1.7; 4.1) | FS pre- frail | 2.4 (1.6; 3.6) | 2.5 (1.7; 3.9) | 2.4 (0.6; 3.7) | 2.0 (1.3; 3.2) |
| PHF | 4.1 (2.8; 5.9) | 4.5 (3.1; 6.6) | 4.1 (2.8; 6.0) | 2.6 (1.6; 4.0) | SOF frail | 2.2 (1.1; 4.3) | 2.4 (1.2; 4.6) | 2.2 (0.1; 4.4) | 1.8 (0.8; 3.7) |
| FS | 3.4 (2.1; 5.4) | 4.0 (2.5; 6.4) | 3.6 (2.3; 5.8) | 1.9 (1.1; 3.3) | SOF pre-frail | 2.3 (1.5; 3.6) | 2.5 (1.6; 3.8) | 2.4 (0.6; 3.7) | 2.2 (1.4; 3.4) |
| FiND | 3.2 (2.1; 4.7) | 3.7 (2.5; 5.5) | 3.4 (2.3; 5.0) | 2.1 (1.3; 3.3) | PFI frail | 1.9 (0.8; 4.4) | 2.1 (0.9; 4.8) | 2.0 (0.8; 4.7) | 1.4 (0.6; 3.5) |
| SOF | 3.0 (2.0; 4.5) | 3.4 (2.3; 5.1) | 3.2 (2.1; 4.8) | 2.3 (1.5; 3.7) | PFI pre frail | 2.2 (1.4; 3.5) | 2.4 (1.5; 3.8) | 2.3 (0.4; 3.7) | 1.9 (1.1; 3.1) |
| ZED2 | 2.8 (2.0; 4.0) | 3.1 (2.2; 4.4) | 3.0 (2.1; 4.2) | 2.1 (1.5; 3.1) | PHF frail | 2.1 (0.8; 5.9) | 2.4 (0.9; 6.6) | 2.2 (0.8; 6.0) | 1.4 (0.5; 4.1) |
| ZED3 | 2.2 (1.4; 3.4) | 2.7 (1.7; 4.1) | 2.4 (1.5; 3.8) | 1.8 (1.1; 2.9) | PHF pre-frail | 1.8 (0.7; 4.6) | 2.0 (0.8; 4.9) | 1.8 (0.7; 4.6) | 1.6 (0.6; 4.0) |
| ZED1 | 2.1 (1.5; 2.9) | 2.4 (1.7; 3.4) | 2.2 (1.6; 3.2) | 1.4 (0.9; 2.1) | ZED3 frail | 1.9 (0.2; 15.7) | 2.1 (0.3; 16.3) | 1.8 (1.2; 2.7) | 1.7 (0.2; 12.8) |
| PFI | 1.9 (1.3; 2.7) | 2.1 (1.5; 3.0) | 2.0 (1.4; 2.9) | 1.4 (1.0; 2.1) | ZED2 frail | 1.9 (0.8; 4.9) | 2.0 (0.8; 5.1) | 2.0 (0.8; 5.1) | 1.5 (0.6; 4.0) |
| BDE | 1.6 (1.1; 2.3) | 1.7 (1.2; 2.5) | 1.6 (1.1; 2.3) | 2.1 (1.4; 3.2) | ZED1 frail | 1.7 (0.7; 4.0) | 1.8 (0.7; 4.3) | 1.8 (0.8; 4.3) | 1.3 (0.5; 3.2) |
|  |  |  |  |  | SPPB frail | 1.7 (0.9; 3.2) | 1.8 (0.9; 3.4) | 1.7 (0.9; 3.3) | 1.3 (0.7; 2.6) |
|  |  |  |  |  | FiND frail | 1.3 (0.6; 2.9) | 1.5 (0.7; 3.2) | 1.4 (0.6; 3.0) | 1.1 (0.5; 2.4) |
| **Multidimensional approach** | | | | | | | | | |
| CSBA | 20.2 (11.2; 36.5) | 15.4 (8.4; 28.2) | 12.6 (6.8; 23.3) | 2.5 (1.2; 5.4) | MFS frail | 1.9 (0.2; 20.6) | 3.9 (0.4; 41.6) | 3.5 (0.3; 37.8) | 2.5 (0.2; 27.5) |
| G8 | 9.2 (5.1; 16.3) | 12.6 (7.1; 22.5) | 10.6 (5.9; 19.1) | 4.0 (2.0; 7.8) | MFS pre-frail | 1.8 (0.2; 19.4) | 3.5 (0.3; 36.9) | 3.3 (0.3; 34.8) | 2.9 (0.3; 30.8) |
| EFS | 8.4 (4.4; 16.0) | 11.2 (5.8; 21.4) | 9.5 (4.9; 18.4) | 5.6 (2.6; 12.3) | CGAST frail | 3.2 (1.3; 8.0) | 3.5 (1.4; 8.8) | 3.2 (0.3; 8.1) | 2.2 (0.8; 5.9) |
| CGAST | 5.7 (3.2; 10.3) | 7.5 (4.2; 13.6) | 6.6 (3.6; 11.9) | 2.6 (1.3; 5.2) | CGAST pre frail | 3.4 (1.4; 8.0) | 3.5 (1.4; 8.2) | 3.3 (0.4; 7.9) | 3.1 (1.3; 7.5) |
| TFI | 4.7 (2.9; 7.7) | 7.1 (4.3; 11.5) | 5.9 (3.6; 9.7) | 3.3 (1.8; 6.0) | FSS frail | 1.8 (0.9; 3.4) | 1.9 (1.0; 3.6) | 1.8 (0.9; 3.4) | 1.2 (0.6; 2.5) |
| MFS | 5.8 (3.8; 8.9) | 6.6 (4.3; 10.1) | 5.8 (3.8; 9.0) | 3.7 (2.4; 5.8) | FSS pre frail | 2.5 (1.6; 4.0) | 2.6 (1.7; 4.1) | 2.5 (0.6; 3.9) | 2.2 (1.4; 3.5) |
| GFI | 4.4 (2.5; 7.7) | 5.8 (3.3; 10.2) | 5.1 (2.9; 9.0) | 1.6 (0.8; 3.3) | G8 frail | 1.9 (1.0; 3.7) | 2.1 (1.1; 4.0) | 1.9 (0.0; 3.7) | 1.3 (0.7; 2.7) |
| IFQ | 4.1 (2.3; 7.1) | 5.6 (3.2; 9.8) | 4.8 (2.8; 8.5) | 2.0 (1.1; 3.8) | IFQ frail | 1.8 (0.7; 5.1) | 2.0 (0.7; 5.6) | 1.9 (0.7; 5.4) | 1.5 (0.5; 4.1) |
| SDFI | 3.2 (1.9; 5.3) | 5.6 (3.3; 9.4) | 4.6 (2.7; 7.8) | 1.5 (0.8; 2.8) | TFI frail | 1.7 (1.0; 2.9) | 1.9 (1.1; 3.1) | 1.8 (0.1; 3.0) | 1.5 (0.8; 2.5) |
| HSF | 3.8 (2.4; 6.0) | 4.2 (2.6; 6.7) | 3.8 (2.4; 6.1) | 1.5 (0.9; 2.6) | CSBA frail | 2.1 (1.2; 3.5) | 1.9 (1.1; 3.3) | 1.8 (0.0; 3.0) | 1.3 (0.7; 2.3) |
| BFI | 1.9 (1.2; 3.1) | 2.8 (1.8; 4.5) | 2.4 (1.5; 3.9) | 1.3 (0.8; 2.2) | EFS frail | 1.6 (1.3; 2.0) | 1.7 (1.4; 2.1) | 1.6 (1.3; 2.1) | 1.3 (0.5; 3.1) |
| FSS | 2.4 (1.6; 3.7) | 2.7 (1.8; 4.2) | 2.5 (1.6; 3.8) | 1.2 (0.7; 2.0) | SDFI frail | 1.5 (0.9; 2.5) | 1.7 (1.0; 2.9) | 1.6 (0.9; 2.7) | 1.2 (0.7; 2.1) |
| SI | 2.1 (1.1; 3.8) | 2.7 (1.4; 4.9) | 2.4 (1.3; 4.5) | 0.9 (0.5; 1.8) | GFI frail | 1.4 (0.9; 2.4) | 1.5 (0.9; 2.6) | 1.5 (0.9; 2.5) | 1.1 (0.6; 1.9) |
| SPQ | 1.8 (1.1; 3.2) | 2.4 (1.4; 4.2) | 2.2 (1.3; 3.9) | 1.1 (0.6; 2.0) | BFI frail | 1.3 (0.6; 2.6) | 1.4 (0.7; 2.9) | 1.3 (0.7; 2.8) | 1.1 (0.5; 2.2) |
|  |  |  |  |  | SI frail | 1.3 (0.5; 3.4) | 1.3 (0.5; 3.7) | 1.3 (0.5; 3.7) | 0.9 (0.3; 2.7) |
|  |  |  |  |  | SPQ frail | 1.1 (0.7; 2.0) | 1.1 (0.6; 1.8) | 1.2 (0.7; 2.0) | 1.0 (0.6; 1.8) |
| **Accumulation of deficits approach** | | | | | | | | | |
| FI40 | 7.2 (3.8; 13.7) | 10.6 (5.6; 20.0) | 9.2 (4.8; 17.6) | 6.9 (3.8; 12.7) | CGA frail | 1.8 (0.9; 3.6) | 2.1 (1.1; 4.2) | 2.0 (0.0; 3.9) | 1.5 (0.7; 3.3) |
| CGA | 5.6 (2.6; 12.2) | 9.4 (4.3; 20.6) | 8.2 (3.7; 18.0) | 3.4 (1.3; 8.6) | CGA pre-frail | 2.3 (1.5; 3.7) | 2.6 (1.6; 4.0) | 2.4 (0.5; 3.9) | 2.2 (1.4; 3.6) |
| FI70 | 5.7 (3.0; 10.6) | 8.9 (4.8; 16.8) | 7.8 (4.1; 14.8) | 5.5 (2.5; 12.0) | FI40 frail | 1.7 (1.0; 2.8) | 1.9 (1.1; 3.1) | 1.8 (0.1; 2.9) | 1.6 (1.1; 2.4) |
| EFIP | 5.4 (2.9; 10.1) | 7.5 (4.0; 14.1) | 6.5 (3.5; 12.3) | 3.8 (1.8; 8.4) | FI70 frail | 1.7 (1.0; 2.8) | 1.9 (1.1; 3.1) | 1.8 (0.1; 2.9) | 1.5 (0.9; 2.7) |
| NLTCS | 5.7 (2.4; 13.6) | 6.8 (2.8; 16.1) | 6.1 (2.5; 14.6) | 1.4 (0.5; 3.8) |  |  |  |  |  |
| FIBLSA | 4.4 (2.2; 8.7) | 5.6 (2.8; 11.2) | 5.1 (2.6; 10.2) | 1.4 (0.6; 3.3) |  |  |  |  |  |
| **Disability approach** | | | | | | | | | |
| HRCA | 3.3 (1.9; 5.6) | 4.3 (2.5; 7.5) | 3.9 (2.3; 6.8) | 1.9 (1.0; 3.6) | HRCA frail | 1.5 (0.9; 2.6) | 1.8 (1.1; 3.0) | 1.7 (0.0; 2.9) | 1.3 (0.7; 2.3) |
| VES13 | 3.3 (2.1; 5.3) | 4.3 (2.7; 6.9) | 3.9 (2.4; 6.3) | 2.2 (1.3; 3.9) | VES13 frail | 1.5 (0.9; 2.5) | 1.7 (1.0; 2.8) | 1.6 (0.0; 2.7) | 1.4 (0.8; 2.4) |
| WHRH | 2.9 (1.8; 4.7) | 3.5 (2.2; 5.7) | 3.3 (2.0; 5.4) | 2.3 (1.3; 4.0) | WHRH frail | 1.5 (0.9; 2.6) | 1.6 (0.9; 2.9) | 1.6 (0.9; 2.8) | 1.1 (0.6; 2.0) |
| SHCFS | 2.5 (1.7; 3.7) | 2.8 (1.9; 4.2) | 2.7 (1.8; 4.0) | 1.3 (0.8; 2.0) | SHCFS frail | 1.5 (0.8; 2.8) | 1.6 (0.9; 3.0) | 1.6 (0.9; 2.9) | 1.1 (0.6; 2.2) |

^1^Model 0= Crude models. ^2^Model 1= HR adjusted by sex. ^3^Model 2= Model 1 + smoking status and alcohol consumption. ^4^Model 3= Model 2 + physical activity, BMI, diabetes, hypertension, cardiovascular, cancer, anemia, COPD, arthritis, neuropsychiatric, depression, cognition, self-rated health & quality of life. Models were fitted using age as time scale, with time 0 = age at entry of study and time 1 =age at event or censoring date.

Abbreviations frailty scores: BDE= Beaver Dam Eye Study Index. BFI= Brief Frailty Index. CGA= Comprehensive Geriatric Assessment. CGAST= Comprehensive Geriatric Assessment Screening Tests. CSBA= Conselice Study of Brain Aging Score. EFIP= Evaluative Frailty Index for Physical Activity. EFS= Edmonton Frail Scale. FI40= 40-item Frailty Index. FI70= 70-item Frailty Index (SHARE). FIBLSA= Frailty Index Beijing Longitudinal Study of Ageing. FiND= Frail Non-Disabled Questionnaire. FS= Frail Scale. FSS= Frailty Staging System. G8= G-8 Geriatric Screening Tool. GFI= Groningen Frailty Indicator. HRCA= Hebrew Rehabilitation Center for Aged Vulnerability Index. HSF= Health Status Form. IFQ= Inter-Frail Questionnaire. MFS= Modified Frailty Score. MPHF= Modified Phenotype of Frailty. NLTCS= Long Term Care Survey Frailty Index. PFI= Physical Frailty Index. PHF= Phenotype of Frailty. SDFI=, Static/Dynamic Frailty Index. SHCFS= Canadian Study of Health and Aging Clinical Frailty Scale·. SI= Screening Instrument. SOF= Study of Osteoporotic Fractures. SPPB= Short Physical Performance Battery. SPQ= Sherbrooke Postal Questionnaire. TFI= Tilburg Frailty Indicator. VES13= Vulnerable Elders Survey. WHRH= WHOAFC & self-reported health. ZED1= ZutPhen Elderly Study (Physical Activity & Low Energy). ZED2= ZutPhen Elderly Study (Physical Activity & Weight Loss). ZED3= ZutPhen Elderly Study (Physical Activity & Low BMI).
