# Supplementary material for: Comparative analysis of the association between 35 frailty scores and cardiovascular events, cancer, and total mortality in an elderly general population in England: An observational study
Source: PLoS Med. 2018 Mar 27;15(3):e1002543. doi: 10.1371/journal.pmed.1002543 (PMC5870943; doi:10.1371/journal.pmed.1002543)
Supplement: S16 Table — (DOCX) [file pmed.1002543.s017.docx]

**S16 Table.** Mortality hazard ratios of frailty scores in participants of 70 years and younger (n=2758) calculated at median time follow-up (3.5 years)

| **Continuous analysis** | | | | | **Cut-off analysis** | | | | |
| --- | --- | --- | --- | --- | --- | --- | --- | --- | --- |
|  | **HR (95% CI)** | **HR (95% CI)** | **HR (95% CI)** | **HR (95% CI)** |  | **HR (95% CI)** | **HR (95% CI)** | **HR (95% CI)** | **HR (95% CI)** |
| **Frailty Score** | **Model 0^1^** | **Model 1^2^** | **Model 2^3^** | **Model 3^4^** | **Frailty Score** | **Model 0^1^** | **Model 1^2^** | **Model 2^3^** | **Model 3^4^** |
| **Phenotype of frailty approach** | | | | | | | | | |
| MPHF | 12.8 (7.4; 22.2) | 13.8 (8.0; 23.8) | 10.6 (6.0; 18.9) | 3.9 (1.0; 10.1) | PHF frail | 6.6 (3.5; 12.5) | 7.1 (3.8; 13.5) | 5.6 (2.9; 10.7) | 2.2 (0.3; 14.7) |
| PHF | 10.8 (5.8; 20.4) | 12.7 (6.7; 23.8) | 9.8 (5.1; 19.0) | 4.8 (1.0; 12.1) | PHF pre-frail | 2.3 (1.3; 4.0) | 2.4 (1.4; 4.3) | 2.2 (1.2; 3.9) | 2.0 (0.5; 8.2) |
| SPPB | 8.9 (3.0; 26.9) | 12.6 (4.8; 32.7) | 6.6 (3.7; 11.7) | 2.3 (1.0; 7.2) | ZED3 frail | 7.4 (3.3; 16.7) | 6.5 (2.9; 14.7) | 4.3 (1.9; 9.8) | 1.7 (0.0; 104.1) |
| FS | 10.5 (6.1; 18.2) | 10.8 (6.3; 18.6) | 8.3 (4.7; 14.6) | 1.9 (1.0; 5.2) | FS frail | 4.5 (3.1; 6.6) | 4.5 (3.1; 6.6) | 1.7 (1.3; 2.3) | 1.5 (0.3; 6.6) |
| FiND | 9.2 (5.5; 15.1) | 9.5 (5.7; 15.6) | 7.2 (4.3; 12.2) | 3.0 (1.0; 7.5) | FS pre- frail | 1.8 (1.4; 2.4) | 1.9 (1.4; 2.5) | 3.6 (2.3; 5.6) | 1.3 (0.6; 3.2) |
| SOF | 7.5 (4.3; 13.1) | 8.0 (4.6; 14.0) | 6.4 (3.2; 12.9) | 2.7 (1.0; 6.7) | ZED1 frail | 5.0 (3.2; 7.9) | 4.8 (3.0; 7.6) | 4.0 (2.5; 6.3) | 1.6 (0.4; 6.9) |
| ZED2 | 6.9 (4.3; 11.2) | 7.3 (4.5; 11.8) | 6.3 (3.9; 10.3) | 3.3 (1.0; 6.6) | ZED2 frail | 5.1 (2.5; 10.3) | 5.0 (2.5; 10.2) | 3.9 (1.9; 8.0) | 1.5 (0.2; 9.7) |
| ZED3 | 5.5 (3.2; 9.5) | 6.4 (3.7; 11.2) | 4.9 (2.8; 8.7) | 2.7 (1.0; 6.7) | SOF frail | 4.0 (2.6; 6.2) | 4.1 (2.7; 6.4) | 1.9 (1.4; 2.5) | 1.9 (0.4; 8.8) |
| ZED1 | 5.6 (3.7; 8.5) | 5.9 (3.9; 9.0) | 4.7 (3.0; 7.3) | 2.2 (1.0; 4.9) | SOF pre-frail | 2.0 (1.5; 2.6) | 2.0 (1.5; 2.7) | 2.3 (1.8; 3.0) | 1.5 (0.6; 3.6) |
| PFI | 4.7 (2.9; 7.4) | 4.9 (3.1; 7.8) | 4.1 (2.5; 6.6) | 1.7 (1.0; 4.2) | PFI frail | 3.2 (1.5; 6.8) | 3.4 (1.6; 7.1) | 3.0 (1.4; 6.5) | 1.4 (0.1; 16.1) |
| BDE | 3.9 (1.7; 9.1) | 3.8 (1.6; 8.7) | 3.1 (1.3; 7.2) | 2.3 (1.0; 5.2) | PFI pre frail | 2.8 (2.0; 3.9) | 2.8 (2.0; 4.0) | 2.4 (1.7; 3.4) | 1.7 (0.5; 5.5) |
|  |  |  |  |  | FiND frail | 2.3 (0.7; 7.5) | 2.4 (0.7; 8.0) | 2.1 (0.6; 7.2) | 1.4 (0.4; 5.1) |
|  |  |  |  |  | SPPB frail | 1.9 (0.8; 4.6) | 1.9 (0.8; 4.7) | 1.8 (0.7; 4.4) | 1.3 (0.5; 3.4) |
| **Multidimensional approach** | | | | | | | | | |
| CSBA | 154.8 (51.9; 461.2) | 145.0 (46.1; 456.4) | 89.9 (27.8; 290.7) | 7.2 (2.0; 34.7) | EFS frail | 5.2 (3.3; 8.1) | 5.0 (3.2; 7.8) | 4.1 (2.6; 6.4) | 2.3 (0.5; 11.7) |
| EFS | 81.9 (36.3; 184.4) | 83.8 (37.5; 187.4) | 57.3 (24.5; 134.1) | 30.5 (2.0; 120.0) | IFQ frail | 3.5 (1.3; 9.5) | 3.4 (1.3; 9.2) | 2.4 (0.9; 6.5) | 1.6 (0.0; 229.5) |
| G8 | 57.5 (21.4; 154.7) | 68.2 (25.3; 183.6) | 40.5 (14.6; 112.2) | 8.1 (2.0; 37.0) | CGAST frail | 3.1 (2.0; 4.8) | 3.3 (2.2; 5.1) | 3.0 (2.0; 4.7) | 1.4 (0.4; 5.2) |
| GFI | 35.1 (16.0; 76.9) | 35.7 (16.4; 77.8) | 23.7 (10.5; 53.6) | 4.5 (2.0; 18.3) | CGAST pre frail | 1.3 (0.9; 2.1) | 1.4 (0.9; 2.2) | 1.4 (0.9; 2.2) | 1.3 (0.4; 3.7) |
| SDFI | 20.0 (8.9; 44.9) | 35.5 (12.5; 101.2) | 21.9 (7.5; 63.8) | 2.7 (2.0; 9.6) | MFS frail | 2.9 (1.7; 4.9) | 3.3 (0.8; 13.1) | 2.6 (1.5; 4.4) | 1.7 (0.4; 7.1) |
| CGAST | 30.6 (14.0; 67.2) | 31.6 (14.6; 68.5) | 23.2 (10.5; 51.2) | 3.1 (2.0; 11.6) | MFS pre-frail | 1.3 (0.8; 2.2) | 1.8 (0.5; 5.7) | 1.3 (0.8; 2.1) | 1.4 (0.4; 4.6) |
| TFI | 24.1 (11.6; 50.3) | 28.3 (11.0; 73.2) | 18.2 (8.4; 39.3) | 8.6 (2.0; 31.0) | G8 frail | 2.9 (1.2; 7.2) | 3.1 (1.2; 7.7) | 2.2 (1.5; 3.3) | 1.7 (0.6; 5.0) |
| IFQ | 24.7 (11.3; 54.0) | 25.5 (11.7; 55.4) | 16.8 (7.5; 37.8) | 3.8 (2.0; 14.0) | FSS frail | 2.6 (1.8; 3.9) | 2.6 (1.8; 3.8) | 2.3 (1.7; 3.0) | 1.1 (0.3; 4.7) |
| HSF | 22.0 (11.4; 42.5) | 21.2 (11.0; 40.6) | 15.6 (7.8; 31.0) | 2.3 (2.0; 9.2) | FSS pre frail | 2.3 (1.7; 3.0) | 2.4 (1.8; 3.2) | 3.9 (2.6; 5.7) | 1.8 (0.7; 4.2) |
| MFS | 6.4 (2.8; 14.9) | 10.4 (4.2; 25.3) | 5.2 (2.2; 12.1) | 3.2 (2.0; 8.2) | SDFI frail | 2.4 (1.8; 3.1) | 2.8 (1.0; 7.4) | 2.4 (0.9; 6.5) | 1.5 (0.5; 4.3) |
| SI | 7.3 (3.6; 14.7) | 9.3 (4.6; 18.5) | 6.8 (4.1; 11.3) | 0.8 (2.0; 3.1) | SPQ frail | 1.6 (1.2; 2.2) | 1.7 (1.3; 2.3) | 1.5 (1.1; 2.0) | 0.9 (0.3; 3.2) |
| BFI | 7.5 (3.2; 17.8) | 8.4 (3.6; 20.0) | 6.1 (2.5; 14.8) | 1.6 (2.0; 4.2) | TFI frail | 2.5 (1.9; 3.3) | 2.6 (2.0; 3.4) | 2.3 (1.8; 3.0) | 1.7 (0.6; 4.6) |
| FSS | 6.9 (3.8; 12.4) | 7.0 (3.9; 12.5) | 5.2 (2.9; 9.5) | 1.3 (2.0; 3.6) | CSBA frail | 2.8 (2.2; 3.7) | 2.6 (2.0; 3.4) | 2.3 (1.7; 3.1) | 1.1 (0.4; 3.2) |
| SPQ | 5.1 (2.2; 11.8) | 5.8 (1.7; 20.2) | 3.7 (1.6; 8.7) | 0.8 (2.0; 3.2) | GFI frail | 2.5 (1.9; 3.2) | 2.5 (1.9; 3.3) | 2.3 (1.7; 3.0) | 1.3 (0.5; 3.5) |
|  |  |  |  |  | SI frail | 1.8 (1.1; 3.0) | 2.0 (1.2; 3.3) | 1.6 (1.0; 2.6) | 0.6 (0.0; 10.3) |
|  |  |  |  |  | BFI frail | 1.9 (1.3; 2.7) | 2.0 (1.3; 2.9) | 1.6 (1.1; 2.4) | 0.8 (0.1; 5.6) |
| **Accumulation of deficits approach** | | | | | | | | | |
| CGA | 76.2 (29.2; 199.3) | 87.2 (33.8; 224.9) | 54.6 (20.1; 148.5) | 24.2 (3.0; 124.0) | CGA frail | 4.1 (2.9; 5.8) | 4.4 (3.1; 6.2) | 3.7 (2.6; 5.3) | 2.8 (0.8; 10.2) |
| NLTCS | 53.8 (19.6; 147.5) | 54.9 (20.0; 150.6) | 33.1 (11.5; 95.0) | 1.4 (3.0; 10.3) | CGA pre-frail | 2.1 (1.5; 2.8) | 2.3 (1.7; 3.1) | 2.1 (1.6; 2.9) | 2.1 (0.9; 4.8) |
| FI70 | 40.4 (18.4; 88.9) | 46.1 (21.2; 100.6) | 30.3 (13.3; 69.1) | 19.3 (3.0; 76.3) | FI70 frail | 2.9 (2.2; 3.8) | 3.1 (2.4; 4.0) | 2.7 (2.1; 3.5) | 2.2 (0.8; 5.8) |
| FI40 | 39.5 (18.5; 84.3) | 44.6 (21.0; 94.7) | 31.0 (13.8; 69.4) | 17.5 (3.0; 52.6) | FI40 frail | 2.5 (1.9; 3.2) | 2.6 (2.0; 3.4) | 1.8 (1.3; 2.3) | 1.8 (0.8; 3.9) |
| FIBLSA | 26.6 (11.7; 60.3) | 30.4 (8.2; 112.9) | 18.6 (7.9; 43.9) | 1.4 (3.0; 7.4) |  |  |  |  |  |
| EFIP | 26.8 (12.5; 57.5) | 27.1 (12.7; 57.7) | 17.4 (7.8; 38.9) | 8.5 (3.0; 33.6) |  |  |  |  |  |
| **Disability approach** | | | | | | | | | |
| VES13 | 16.7 (9.0; 31.2) | 17.9 (9.7; 33.1) | 13.1 (6.9; 25.1) | 5.4 (4.0; 17.5) | SHCFS frail | 3.0 (2.2; 4.1) | 3.0 (2.2; 4.1) | 2.5 (1.8; 3.5) | 1.2 (0.4; 3.8) |
| HRCA | 9.0 (4.8; 16.7) | 9.5 (5.1; 17.5) | 6.5 (3.4; 12.6) | 0.8 (4.0; 3.0) | VES13 frail | 2.7 (2.0; 3.5) | 2.8 (2.1; 3.6) | 2.4 (1.8; 3.2) | 1.6 (0.5; 4.6) |
| SHCFS | 8.8 (5.4; 14.4) | 8.9 (5.5; 14.4) | 19.4 (8.4; 45.0) | 2.0 (4.0; 4.7) | HRCA frail | 2.5 (1.9; 3.2) | 2.7 (2.1; 3.5) | 2.4 (1.8; 3.1) | 1.3 (0.4; 3.6) |
| WHRH | 7.3 (4.5; 11.9) | 7.8 (4.8; 12.6) | 6.1 (3.6; 10.1) | 2.7 (4.0; 6.8) | WHRH frail | 2.5 (1.9; 3.4) | 2.6 (1.9; 3.4) | 2.2 (1.6; 3.0) | 0.8 (0.2; 2.5) |

^1^Model 0= Crude models. ^2^Model 1= HR adjusted by sex. ^3^Model 2= Model 1 + smoking status and alcohol consumption. ^4^Model 3= Model 2 + physical activity, BMI, diabetes, hypertension, cardiovascular, cancer, anemia, COPD, arthritis, neuropsychiatric, depression, cognition, self-rated health & quality of life. Models were fitted using age as time scale, with time 0 = age at entry of study and time 1 =age at event or censoring date.

Abbreviations frailty scores: BDE= Beaver Dam Eye Study Index. BFI= Brief Frailty Index. CGA= Comprehensive Geriatric Assessment. CGAST= Comprehensive Geriatric Assessment Screening Tests. CSBA= Conselice Study of Brain Aging Score. EFIP= Evaluative Frailty Index for Physical Activity. EFS= Edmonton Frail Scale. FI40= 40-item Frailty Index. FI70= 70-item Frailty Index (SHARE). FIBLSA= Frailty Index Beijing Longitudinal Study of Ageing. FiND= Frail Non-Disabled Questionnaire. FS= Frail Scale. FSS= Frailty Staging System. G8= G-8 Geriatric Screening Tool. GFI= Groningen Frailty Indicator. HRCA= Hebrew Rehabilitation Center for Aged Vulnerability Index. HSF= Health Status Form. IFQ= Inter-Frail Questionnaire. MFS= Modified Frailty Score. MPHF= Modified Phenotype of Frailty. NLTCS= Long Term Care Survey Frailty Index. PFI= Physical Frailty Index. PHF= Phenotype of Frailty. SDFI=, Static/Dynamic Frailty Index. SHCFS= Canadian Study of Health and Aging Clinical Frailty Scale·. SI= Screening Instrument. SOF= Study of Osteoporotic Fractures. SPPB= Short Physical Performance Battery. SPQ= Sherbrooke Postal Questionnaire. TFI= Tilburg Frailty Indicator. VES13= Vulnerable Elders Survey. WHRH= WHOAFC & self-reported health. ZED1= ZutPhen Elderly Study (Physical Activity & Low Energy). ZED2= ZutPhen Elderly Study (Physical Activity & Weight Loss). ZED3= ZutPhen Elderly Study (Physical Activity & Low BMI).
